# Pro219 is an electrostatic color determinant in the light-driven sodium pump KR2

**DOI:** 10.1038/s42003-021-02684-z

**Published:** 2021-10-13

**Authors:** Yuta Nakajima, Laura Pedraza-González, Leonardo Barneschi, Keiichi Inoue, Massimo Olivucci, Hideki Kandori

**Affiliations:** 1 Department of Life Science and Applied Chemistry, Nagoya Institute of Technology, Showa-ku, Nagoya, 466-8555 Japan; 2 Dipartimento di Biotecnologie, Chimica e Farmacia, Università degli Studi di Siena, Via Aldo Moro, 2, I-53100 Siena, Italy; 3 The Institute for Solid State Physics, The University of Tokyo, 5-1-5 Kashiwanoha, Kashiwa, Chiba, 277-8581 Japan; 4 Department of Chemistry, Bowling Green State University, Bowling Green, OH 43403 USA; 5 OptoBioTechnology Research Center, Nagoya Institute of Technology, Showa-ku, Nagoya, 466-8555 Japan

**Keywords:** Computational biophysics, Biophysical chemistry

## Abstract

Color tuning in animal and microbial rhodopsins has attracted the interest of many researchers, as the color of their common retinal chromophores is modulated by the amino acid residues forming the chromophore cavity. Critical cavity amino acid residues are often called “color switches”, as the rhodopsin color is effectively tuned through their substitution. Well-known color switches are the L/Q and A/TS switches located in the C and G helices of the microbial rhodopsin structure respectively. Recently, we reported on a third G/P switch located in the F helix of the light-driven sodium pumps of KR2 and *Js*NaR causing substantial spectral red-shifts in the latter with respect to the former. In order to investigate the molecular-level mechanism driving such switching function, here we present an exhaustive mutation, spectroscopic and computational investigation of the P219X mutant set of KR2. To do so, we study the changes in the absorption band of the 19 possible mutants and construct, semi-automatically, the corresponding hybrid quantum mechanics/molecular mechanics models. We found that the P219X feature a red-shifted light absorption with the only exception of P219R. The analysis of the corresponding models indicate that the G/P switch induces red-shifting variations via electrostatic interactions, while replacement-induced chromophore geometrical (steric) distortions play a minor role. However, the same analysis indicates that the P219R blue-shifted variant has a more complex origin involving both electrostatic and steric changes accompanied by protonation state and hydrogen bond networks modifications. These results make it difficult to extract simple rules or formulate theories for predicting how a switch operates without considering the atomistic details and environmental consequences of the side chain replacement.

## Abstract (web summary)

Nakajima, Pedraza-González et al. provide a comprehensive investigation of amino acid mutations at position 219 of the sodium pump rhodopsin, KR2, and their role in the color tuning of the retinal chromophore. They prepared P219X (X= A, C, D, E, F, G, H, I, K, L, M, N, Q, R, S, T, V, W, Y) mutants of KR2, and find that all mutants are red-shifted, except for P219R, highlighting its role as a color determinant in the light-driven pump KR2.

## Introduction

Microbial or animal rhodopsins contain either an all-*trans* or 11-*cis* retinal chromophore respectively^1^. In both cases the chromophore is located inside the seven-helix (TM1 to TM7) transmembrane structure of diverse opsins where it binds, covalently, a lysine residue to form a Schiff base linkage^2^. In such an environment the chromophore spectroscopic and reactivity properties are modulated by its molecular environment as demonstrated by the variety of displayed colors and, therefore, variations in the wavelength (λ_max_) of the maximum of the absorption bands^3–9^. The mechanism allowing such “color tuning” effect is an important topic in rhodopsin research since the λ_max_ value of the light captured by a specific rhodopsin represents a biological functions^10–14^. While the color tuning mechanism^7–9^ is still not fully understood, it is apparent that it must be determined by the interactions between the chromophore and the surrounding amino acid residues featuring side-chains which may be charged, dipolar, aromatic and capable of hydrogen-bonding and steric contact effects. Accordingly, learning the precise “rules” for controlling such interaction appears to be of basic importance for the understanding of different facets of rhodopsin biology including their evolution, ecology, biophysics and the laboratory engineering required for optogenetic applications^15–17^.

In most cases, the all-*trans* retinal chromophore of microbial rhodopsins features a protonated, Schiff base linkage (rPSBAT) and such -CH = NH( + )- state is stabilized by a negatively charged -COO( − ) counterion, a glutamate or aspartate, placed in its vicinity. While the electrostatic interaction between chromophore and counter-ion is prominent in color tuning, other specific color determining residues have been reported, which are sometimes called “color switches”. In the case of microbial rhodopsin, a famous color switch is the “L/Q switch” in proteorhodopsin^10,18^. In fact, green-absorbing (GPR) and blue-absorbing (BPR) proteorhodopsins contain Leu and Gln at position 105 of C-helix (TM3), respectively. It has been proposed that the L/Q selection provides a mechanism for optimizing light absorption with respect to specific environmental light conditions^10,19^. In other words, the color regulation would enable bacteria living in shallow and deep ocean waters to use green and blue light respectively which is abundant in these respective habitats. Although Leu is hydrophobic and Gln is hydrophilic, comprehensive mutation study of L105 in GPR indicated that molecular volume (i.e., steric interactions), not hydrophobicity, is correlated with the λ_max_ value^20^.

Another color switch is the “A/T switch”^3^. The alanine residue at position of 215 in bacteriorhodopsin (BR) is known to partly contribute to the spectral difference between the BR and *Natronomonas pharaonis* sensory rhodopsin II (*Np*SRII, also *pharaonis* phoborhodopsin) as it is replaced with Thr in *Np*SRII^3^. This is considered to be related to the evolution from BR to *Np*SRII^21^. A previous study indicates that the S254 of a bacterial light-driven sodium pump from *Krokinobacter eikastus* (KR2) also plays a role similar to *Np*SRII and BR^22^. Hence, here we refer to it as the “A/TS switch”, as it appears to represent an additional example of naturally occurring color determining residue. This switch satisfies the general principle of color tuning, where introduction of a polar residue in the vicinity of the β-ionone ring or the Schiff base moiety of the chromophore causes spectral red and blue shift, respectively^14,23–25^. This observation is in line with the present theoretical understanding of color tuning based on the electronic structure and, therefore, opposite positive charge distribution, of the ground (S_0_) and first excited (S_1_) states of rPSBAT. In short, while in S_0_ the positive charge is mainly localized in the -CH = NH- moiety of the chromophore, in the S_1_ the positive charge is delocalized towards the β-ionone ring. Accordingly, negatively charge atoms located in the vicinity of the Schiff base moiety would stabilize S_0_ with respect to S_1_ leading to a blue-shift of the λ_max_ value. In contrast, if the negative atoms are located in the vicinity of the β-ionone ring they would stabilize S_1_ with respect to S_0_ leading to a red-shift. Of course, positively charged atoms will have an opposite effect^7^.

**Fig. 1:**
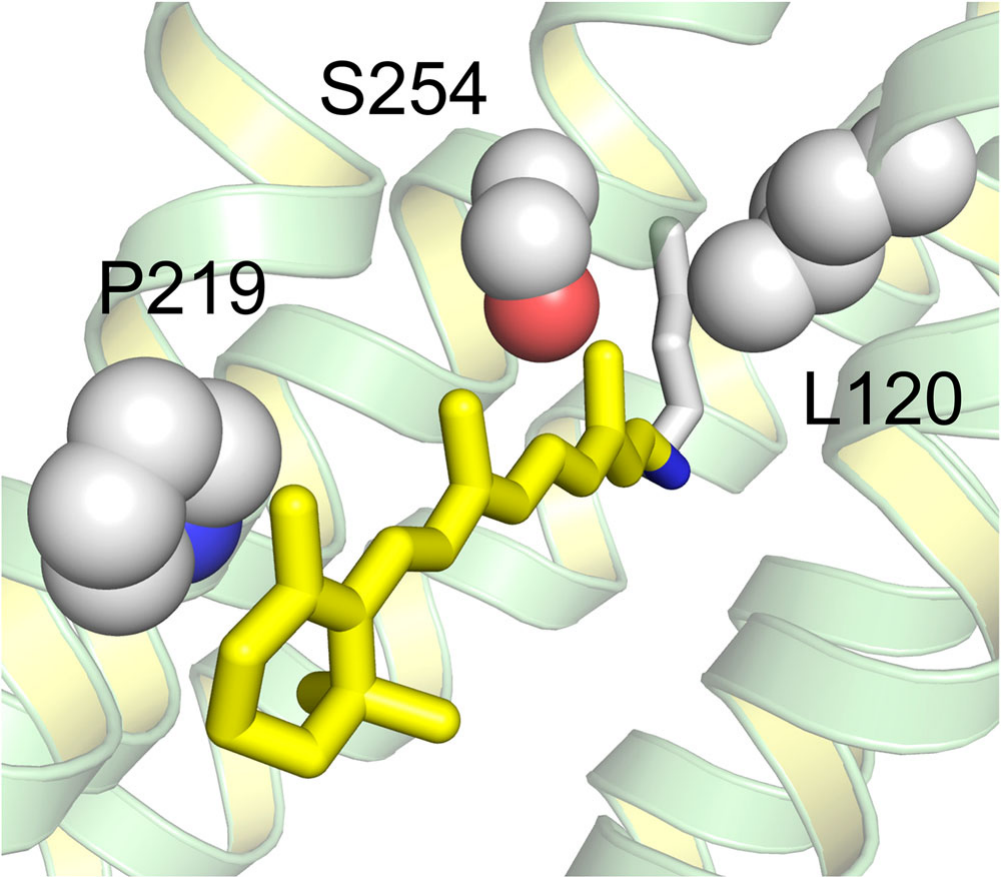
Color switches of microbial rhodopsins, shown in the structure of a light-driven sodium pump KR2. KR2 has L120, S254, and P219 in the L/Q switch, A/TS switch, and G/P switch, respectively.

Recently, a combined experimental and computational study carried out by some of the authors revealed that certain mutations at P219 in KR2 led to a spectral red-shift with no loss of its sodium pump function^22^. As far as color tuning is concerned, Pro is an unusual residue. In fact, this residue is highly conserved in most microbial rhodopsins, but two light-driven sodium pumping rhodopsins (NaRs) that do not conserve it were identified from *Parvularcula oceani*^26^ (*Po*NaR) and *Jannaschia seosinensis*^27^ (*Js*NaR). These rhodopsins display a Thr and Gly residue at the P219 position of KR2, respectively (see Fig. 1). Although the former was reported in the mention previous study, it was not expressed in *E. coli* cells^28^. In contrast, it was possible to express *Js*NaR, and a mutation study showed that it represents a third color switch that was named “G/P switch”^22^. This role of residue 219 is supported by the fact that the λ_max_ value of KR2 (525 nm) is red-shifted to 535 nm in its P219G mutant and the fact that the *Js*NaR λ_max_ value (550 nm) is blue-shifted to 523 nm in its G216P mutant. However, the exact mechanism that is at the basis of such color tuning effect is unknown.

We find that KR2 represents a suitable laboratory model for an in-depth investigation of the G/P switching mechanism. At the same time a molecular-level understanding of such a phenomenon must necessarily be based on the construction of a light-responsive computational model of the protein. Such a model has to incorporate a description of the electronic structure of rPSBAT. Accordingly, in this work we present an exhaustive investigation of the color tuning induced by point mutation at the P219 position of KR2. We prepare all possible P219X mutants where X stands for all alternative 19 natural amino acids. The resulting color variations, spanning a red-to-blue range going from 545 nm to 515 nm, provides a unique basis for molecular-level mechanistic studies performed using hybrid quantum mechanics/molecular mechanics (QM/MM) models generated with the Automatic Rhodopsin Modeling (*a*-ARM) protocol^9^, equipped with multiconfigurational second order perturbation theory (see “Materials and Methods” section). By systematically building and analyzing congruously built QM/MM models of the wild-type and mutants (20 models in total), we support the hypothesis that the 219 position induces color changes through, essentially, electrostatic effects while steric effects contribute to a single, blue-shifting P219R mutant. Thus, the putative P/X color switch would be, mostly, electrostatically driven when causing a red-shift but uses a more complex mechanism for a blue-shift. To the best of our knowledge, this manuscript represents the first study of site-saturation mutagenesis for a color switch that has been carried out both experimentally and computationally on the same mutant set.

## Results and discussion

### Absorption spectra of P219X mutants of KR2

We attempted to express the 19 different P219X mutants of KR2 in *E. coli* where all-*trans* retinal was added at the induction period to produce the rPSBAT chromophore. Supplementary Note 1 shows pictures of WT and 19 P219 mutants (see Supplementary Fig. 1). As is seen from the color, expression was much lower for basic amino acids such as His, Lys and Arg. The expression levels are quantitatively compared in Supplementary Fig. 2, where all the P219X mutants are enough expressed to test their absorption maxima.

**Fig. 2:**
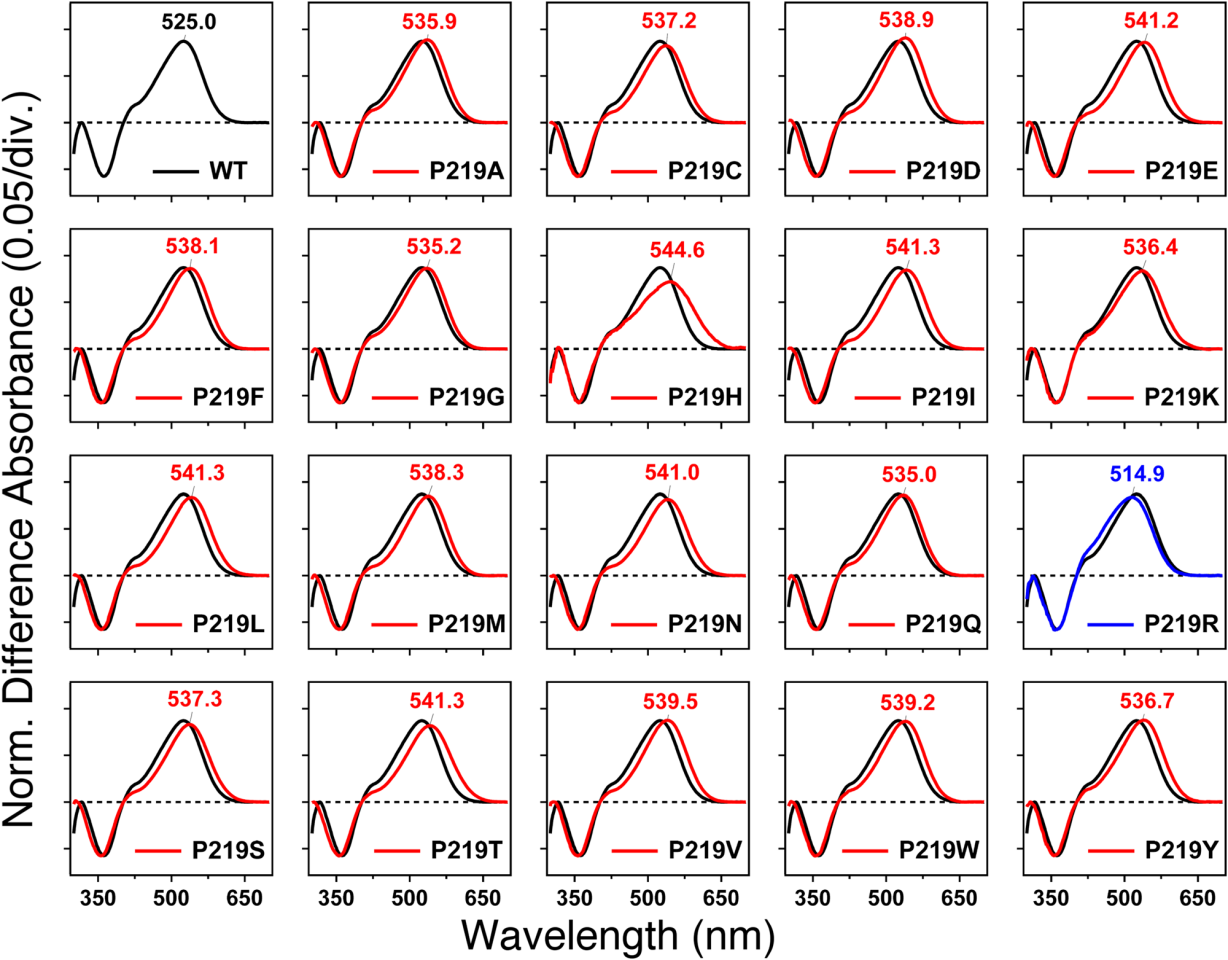
Light-induced difference absorption spectra of the WT-KR2 (black curves) and 19 P219X mutants (colored curves) of KR2 in the presence of 500 mM hydroxylamine. Positive and negative signals show the spectra before and after illumination corresponding to those of the rhodopsin and retinal oxime, respectively. Red and blue curves represent red- and blue-shifted mutants, respectively. Mutant and WT-KR2 spectra were normalized by use of a negative peak at 361 nm.

To quantify the absorption properties of each mutant, the sample was illuminated in the presence of hydroxylamine. This process converts protein-bound retinal chromophore into retinal oxime by light, so that one can easily obtain the λ_max_ of each protein without purification. Figure 2 shows the change in absorption (before-minus-after illumination) representing the photobleaching of WT-KR2 and 19 mutants in the presence of 500 mM hydroxylamine. In WT-KR2 (black curve), positive and negative peaks appeared at 525 and 361 nm, corresponding to the unphotolyzed protein and retinal oxime, respectively. The mutant spectra were normalized to the WT-KR2 spectrum by use of a negative peak at 361 nm.

**Fig. 3:**
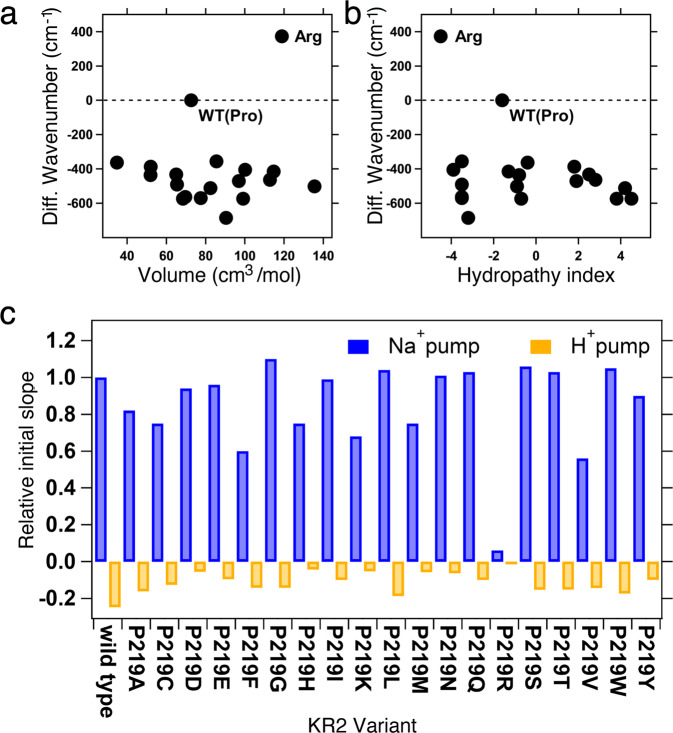
Experimental Data. Correlation between the absorption light energy and (**a**) the volume or (**b**) hydropathy index of the amino acid residue at position 219 in KR2. The y-axis represents the difference in wavenumber from that of the wild type (reciprocal *λ*_max_; cm^−1^), where positive and negative values correspond to the spectral blue- and red-shifts, respectively. (**c**) Quantitative comparison of pump activities of the WT-KR2 and mutant KR2. The numbers of protons taken in or released from the cells by the pump activity of KR2 and each mutant per one protein molecule in one second are shown. The values were estimated from the initial slope of light induced pH changes and the expression levels of the proteins. *E. coli* cells suspensions expressing KR2 mutants are illuminated at >520 nm light, and quantified proton release and uptake are measured in the solution containing 100 mM NaCl (blue bars) and CsCl (orange bars). Positive values indicate the numbers of protons which are taken into cells by one protein molecule per second, which originates from outward Na^+^ pump. Negative values indicate the numbers of protons which are released from cells by one protein molecule per second, which originates from outward proton pump.

Blue and red curves in Fig. 2 show light-induced difference absorption spectra (before-minus-after illumination) of mutants, which exhibit spectral blue and red shifts, respectively. While negative peaks due to retinal oxime were identical for WT-KR2 and mutants, all mutants showed spectral red-shifts, corresponding to λ_max_ in the 535–541 nm range. The only exception was P219R, whose λ_max_ is located at 515 nm. It is likely that Arg is positively charged even in the hydrophobic environment surrounding position 219. Consistently with the color tuning theory mentioned above, such positive charge, placed near the β-ionone ring of rPSBAT, would cause a spectral blue shift. On the other hand, in Fig. 3 we report the observed spectral shift in energy (in wavenumber) versus volume (Fig. 3a) and hydropathy (Fig. 3b) of amino acids. The figure shows that for 18 mutants the λ_max_ correlate with neither volume nor hydropathy, whereas the blue-shifted Arg is unique as it has both higher volume and lower hydropathy than Pro. The higher volume of Arg would suggest that the observed blue-shift is related to a steric effect. Below we show that, according to our computational analysis, this effect alone would not explain the observed P219R spectral change.

Figure 3c compares ion-transport activity using a pH electrode. The sodium and proton pump activities of the WT-KR2 is maintained except for P219R. This fact suggests that the addition of a positive charge at that specific position is responsible for the lack of ion transport. Both absorption spectra (Fig. 2) and transport activity (Fig. 3c) suggest that other amino acid replacements lead to neutral side-chain. In fact, it is reasonable for Asp, Glu, and His to be neutral in the hydrophobic environment. In addition, Lys may also be neutral in the P219K mutant. All these assigned protonation states are consistent with those of the constructed *a*-ARM QM/MM models, as described in next section.

### Construction of the WT model

**Fig. 4:**
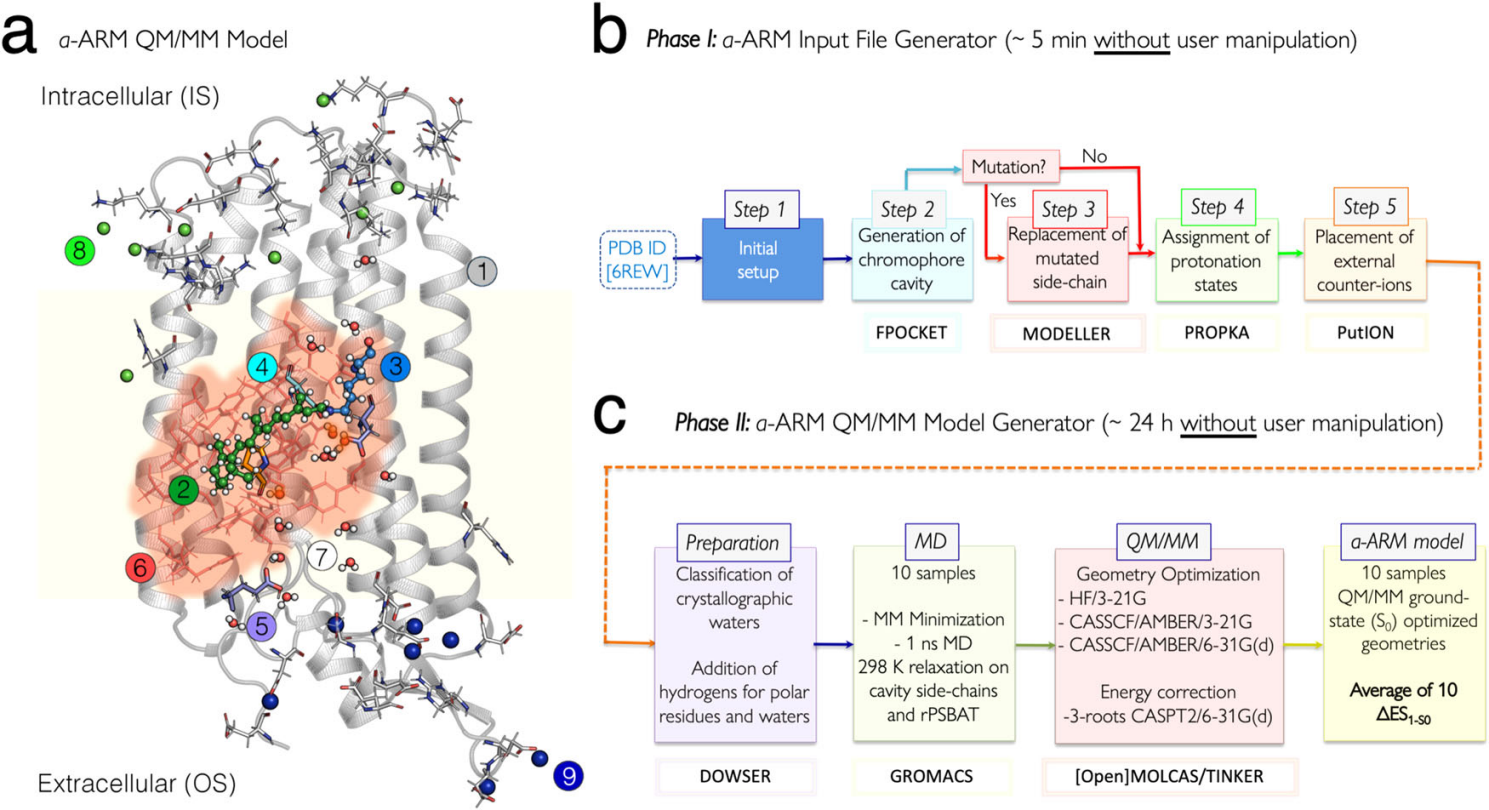
Structure of *a*-ARM protocol. (**a**) General scheme of a monomeric, gas-phase and globally uncharged QM/MM model for the WT-KR2 rhodopsin, generated by the *a*-ARM rhodopsin model building protocol. This is composed of: (1) environment subsystem (silver cartoon), (2) retinal chromophore (green tubes), (3) Lys side-chain covalently linked to the retinal chromophore (blue tubes), (4) main counter-ion MC (cyan tubes), (5) protonated residues, (6) residues of the chromophore cavity subsystem (red tubes), (7) water molecules, and external (8) Cl^−^ (green balls) and (9) Na^+^ (blue balls) counterions. Parts 2 and 3 form the Lys-QM subsystem which includes the H-link atom located along the only bond connecting blue and green atoms. Parts 4 and 6 form the cavity subsystem. The water molecules (Part 7) may be part of the environment or cavity subsystems. The external OS and IS charged residues are shown in frame representation. The residue P219 is presented as orange tubes. (right) General workflow of the *a*-ARM rhodopsin model building protocol for the generation of QM/MM models of wild-type and mutant rhodopsins. The *a*-ARM protocol comprises two phases: (**b**) input file generator phase and (**c**) QM/MM model generator phase. The different software used in each step are also specified.

The QM/MM models (see Fig. 4a) were generated by using the *a*-ARM protocol, whose workflow is illustrated in Fig. 4b, c and described in the “Materials and Methods” section (see Supplementary Note 2 and Supplementary Note 3). The *a*-ARM_default_ model for the WT-KR2 was automatically generated taking, as the only input, the X-ray crystallographic of the pentameric form resolved at 2.2 Å (PDB ID 6REW^29^). The following parameters were automatically selected by the Input file generator: Chain A of the X-ray crystallographic structure; rotamer N84 with occupancy number of 0.50 (step 1 in Fig. 4b); 24 chromophore cavity residues defined based on Voronoi tessellation and alpha spheres theory, and including the K255 residue covalently linked to the chromophore, plus the D116 main (MC), D251 secondary (SC) counter-ion residues and 3 molecules of water (step 2 in Fig. 4b); protonation states predicted at pH 5.2, as: neutral E160 (step 4 in Fig. 4b); and the inclusion of 8 Cl^−^ inner (IS) 7 Na^+^ outer (OS) external counter-ions, with positions optimized with respect to an electrostatic potential grid constructed around each charged target residue. The Na^+^ ion present in the X-ray structure was kept, for a total of 8 Na^+^ ions in the model (step 5 in Fig. 4b). Such parameters ultimately led to an *a*-ARM_default_ model producing a *λ*_max_ (in terms of the average vertical excitation energy of 10 replicas, see the “Materials and Methods” section) of about 127 nm (0.75 eV in terms of ΔE_S1–S0_) blue-shifted with respect to the experimental data. In fact, as documented elsewhere^9,30^, the default models do not always replicate the correct electrostatics of the chromophore counterion complex in ion-pumping rhodopsins^9,30^. For this reason, a customized model was produced (see Supplementary Fig. 3 and Supplementary Table 1). The model customization employs the *a*-ARM_customized_ approach of ref. ^9^. (see also Supplementary Note 2) that guarantees reproducibility. While the default model predicts that both aspartic acid residues forming the counterion complex of the rPSBAT, namely D116 and D251^31^, are negatively charged, we have observed that the presence of two negative charges in the counterion complex would outbalance the positive charge of the rPSBAT, generating a large, blue-shifting effect^9^ in the λ_max_ value. The *a*-ARM_customized_ approach allows to reassign the protonation states (step 4 of the input file generator in Fig. 4b) of D116 and D251. More specifically, as illustrated in Supplementary Fig. 3 and Supplementary Table 2, these protonation states are systematically scanned. It was found that a model featuring a protonated (i.e., neutral) secondary counterion (SC) D251 counterbalanced the charge in the vicinity of the rPSBAT by mitigating the overstabilization of the S_0_ positive charge of the chromophore Schiff base region yielding a smaller, blue-shifted error of about 14 nm (0.07 eV). Notice that in QM/MM modeling, it is a common practice to evaluate the protonation states of the rPSB counterion complex by looking, as a guidance, at the reproducibility of the experimental *λ*_max_ (see for instance refs. ^13,32,33^).

### Construction of the P219X mutant models

The customized protonation states of the WT-KR2 *a*-ARM_customized_ model were employed for the construction of the “default” P219X mutant models. Notice that, as in the WT-KR2 case, the initial structure provided to the Input file generator phase for each mutant was the X-ray structure. As a result,16 out of 19 models featured ΔE_S1–S0_ values with an error, with respect to the observed value and therefore lower than 3.0 kcal mol^−1^ (0.13 eV) threshold. Only 3 models, P219R, P219K, and P219H, were further customized. The default P219R model features a 67 nm (0.36 eV) blue-shifted ΔE_S1–S0_ with respect to the observed value. This indicates that the positively charged arginine side-chain, located near the β-ionone ring of rPSBAT, leads to a too strong S_1_ destabilization (a positively charge β-ionone region is typical of the S_1_ state of the chromophore). In order to moderate such an effect, (i) the D116 was protonated (i.e., neutral) and the D251 was modeled as negatively charged, accordingly with the customization protocol reported in Supplementary Note 2, and (ii) the E160 residue in the R219 region, assigned to be neutral by step 4 of the default approach, was deprotonated assuming that the positive charge in position 219 increases the group pKa (see Supplementary Table 2). Such customization yields a model just 14 nm (0.07 eV) blue shifted. In the case of P219K, the lysine residue had to be assigned as neutral since the protonated lysine selected by the default protocol yielded a strongly blue-shifted ΔE_S1–S0_ value with respect to the experiment. Finally, for P219H, the histidine residue had to be modeled with the neutral tautomer having the ε nitrogen unprotonated (contrary to the default selection).

The final protonation states of the twenty QM/MM models are: neutral D251, E160 for WT-KR2 and P219X with X = A, C, F, G, I, L, M, N, Q, S, T, V, W, Y; neutral D116 for P219R; neutral D251, E160, H219 (with hydrogen in the ε nitrogen) for P219H; neutral D251, E160, K219 for P219K; neutral D251, E160, E219 for P219E; and neutral D251, E160, D219 for P219D. Notice that, when appropriate, the external counter-ions were automatically updated in step 5 (see Fig. 4b), where an additional Na^+^ was included in the OS. The other parameters for the customized inputs (i.e., chain, rotamers, cavity) remain the same as the described for the customized WT-KR2 model in Supplementary Table 1.

### Simulation of the λ_max_ variation

As mentioned above, the *a*-ARM_customized_ model of WT-KR2 has been selected as a suitable template for mutant modeling as it reproduces the observed λ_max_ (525 nm) equivalent to a vertical excitation energy $\varDelta {{{{{{\rm{E}}}}}}}_{{{{{{\rm{S1}}}}}}-{{{{{\rm{S0}}}}}}}^{{{{{\mathrm{Exp}}}}}}$ = 54.5 kcal mol^−1^^22^ (2.36 eV). The discrepancy falls within the 3.0 kcal mol^−1^ (0.13 eV) error bar established for the *a*-ARM protocol^9,34,35^. This result is reported in Table 1, together with both the computed $(\varDelta {{{{{{\rm{E}}}}}}}_{{{{{{\rm{S1}}}}}}-{{{{{\rm{S0}}}}}}}^{{{{{{\rm{a}}}}}}-{{{{{\rm{ARM}}}}}}})$ and observed $(\varDelta {{{{{{\rm{E}}}}}}}_{{{{{{\rm{S1}}}}}}-{{{{{\rm{S0}}}}}}}^{{{{{\mathrm{Exp}}}}}})$ vertical excitation energies, the corresponding oscillator strengths (f_Osc_), and their difference ${\Delta \Delta {{{{{\rm{E}}}}}}}_{{{{{{\rm{S1}}}}}}-{{{{{\rm{S0}}}}}}}^{{{{{\mathrm{Exp}}}}},{{{{{\rm{a}}}}}}-{{{{{\rm{ARM}}}}}}}$ for the representative model (i.e., replica with $\varDelta {{{{{{\rm{E}}}}}}}_{{{{{{\rm{S1}}}}}}-{{{{{\rm{S0}}}}}}}^{{{{{{\rm{a}}}}}}-{{{{{\rm{ARM}}}}}}}$ closest to the average). This is calculated as ${\Delta \Delta {{{{{\rm{E}}}}}}}_{{{{{{\rm{S1}}}}}}-{{{{{\rm{S0}}}}}}}^{{{{{\mathrm{Exp}}}}},{{{{{\rm{a}}}}}}-{{{{{\rm{ARM}}}}}}}=\varDelta {{{{{{\rm{E}}}}}}}_{{{{{{\rm{S1}}}}}}-{{{{{\rm{S0}}}}}}}^{{{{{{\rm{a}}}}}}-{{{{{\rm{ARM}}}}}}}-\varDelta {{{{{{\rm{E}}}}}}}_{{{{{{\rm{S1}}}}}}-{{{{{\rm{S0}}}}}}}^{{{{{\mathrm{Exp}}}}}}$. The WT-KR2 $\varDelta {{{{{{\rm{E}}}}}}}_{{{{{{\rm{S1}}}}}}-{{{{{\rm{S0}}}}}}}^{{{{{{\rm{a}}}}}}-{{{{{\rm{ARM}}}}}}}$ value is 56.0 ± 0.1 kcal mol^−1^ (2.42 eV, 511 nm) and differs from the experimental data of just 1.5 kcal mol^−1^ (0.07 eV, −14 nm). However, as discussed below, such accuracy limit does not necessarily impact λ_max_ trends that are not affected by systematic errors. Detailed information of the row data is provided in Supplementary Note 4 (see Supplementary Table 3).

**Table 1:** *a*-ARM QM/MM models for the wild-type KR2 (WT-KR2) rhodopsin and 19 of its mutants (P219X, with X = A, C, D, E, F, G, H, I, K, L, M, N, Q, R, S, T, V, W, Y). First vertical excitation energy (∆E_S1−S0_), maximum absorption wavelength (λ^a^_max_), transition oscillator strength (f_Osc_), and difference between calculated and experimental data $({\Delta \Delta {{{{{\rm{E}}}}}}}_{{{{{{\rm{S1}}}}}}-{{{{{\rm{S0}}}}}}}^{{{{{\mathrm{Exp}}}}},{{{{{\rm{a}}}}}}-{{{{{\rm{ARM}}}}}}})$, for the representative QM/MM model.

Variant	Experimental			*a*-ARM (*N* = 1)^a^				Experimental vs *a*-ARM		
	$\varDelta {{{{{{\rm{E}}}}}}}_{{{{{{\rm{S1}}}}}}-{{{{{\rm{S0}}}}}}}^{{{{{\mathrm{Exp}}}}}}$		${{{\lambda }}}_{{{{{{\rm{max }}}}}}}^{{{{{{\rm{a}}}}}},{{{{\mathrm{Exp}}}}}}$	$\varDelta {{{{{{\rm{E}}}}}}}_{{{{{{\rm{S1}}}}}}-{{{{{\rm{S0}}}}}}}^{{{{{{\rm{a}}}}}}-{{{{{\rm{ARM}}}}}}}$		${{{\lambda }}}_{{{{{{\rm{max }}}}}}}^{{{{{{\rm{a}}}}}},{{{{{\rm{a}}}}}}-{{{{{\rm{ARM}}}}}}}$	f_Osc_	${\Delta \Delta {{{{{\rm{E}}}}}}}_{{{{{{\rm{S1}}}}}}-{{{{{\rm{S0}}}}}}}^{{{{{\mathrm{Exp}}}}},{{{{{\rm{a}}}}}}-{{{{{\rm{ARM}}}}}}}$		
	(kcal mol^−1^)	(eV)	(nm)	(kcal mol^−1^)	(eV)	(nm)		(kcal mol^−1^)	(eV)	(nm)
WT	54.5	2.36	525	56.0	2.43	511	1.15	1.5	0.07	−14
P219A	53.4	2.31	536	55.8	2.42	512	1.16	2.4	0.11	−23
P219C	53.2	2.31	537	55.4	2.40	516	1.20	2.2	0.09	−21
P219D	53.1	2.30	539	55.0	2.39	520	1.22	2.0	0.09	−19
P219E	52.8	2.29	541	54.8	2.37	522	1.24	1.9	0.08	−19
P219F	53.1	2.30	538	54.9	2.38	521	1.22	1.8	0.08	−18
P219G	53.4	2.32	535	55.8	2.42	513	1.17	2.4	0.10	−23
P219H	52.5	2.28	545	54.5	2.36	525	1.22	2.0	0.09	−20
P219I	52.8	2.29	541	55.4	2.40	516	1.22	2.6	0.11	−25
P219K	53.3	2.31	536	54.8	2.38	521	1.22	1.5	0.07	−15
P219L	52.8	2.29	541	55.1	2.39	519	1.20	2.3	0.10	−22
P219M	53.1	2.30	538	54.8	2.37	522	1.21	1.7	0.07	−16
P219N	52.8	2.29	541	54.5	2.36	524	1.21	1.7	0.07	−17
P219Q	53.4	2.32	535	54.9	2.38	521	1.23	1.5	0.06	−14
P219R	55.5	2.41	515	57.0	2.47	501	1.12	1.5	0.06	−13
P219S	53.2	2.30	538	54.7	2.37	523	1.24	1.5	0.07	−15
P219T	52.8	2.29	541	55.5	2.40	516	1.19	2.6	0.11	−26
P219V	53.0	2.30	540	54.6	2.37	523	1.31	1.6	0.07	−16
P219W	53.0	2.30	539	55.1	2.39	519	1.22	2.1	0.09	−20
P219Y	53.3	2.31	537	55.2	2.39	518	1.19	1.9	0.08	−18
							MAE	1.9		
							AD_max_	2.6		
							MAD	0.3		

^a^Replica with $\varDelta {{{{{{\rm{E}}}}}}}_{{{{{{\rm{S1}}}}}}-{{{{{\rm{S0}}}}}}}^{{{{{{\rm{a}}}}}}-{{{{{\rm{ARM}}}}}}}$ closest to the average.

MAE: Mean absolute error, AD_max_: Maximum absolute error, MAD: Mean absolute deviation. See definitions in Supplementary Note 6.

**Fig. 5:**
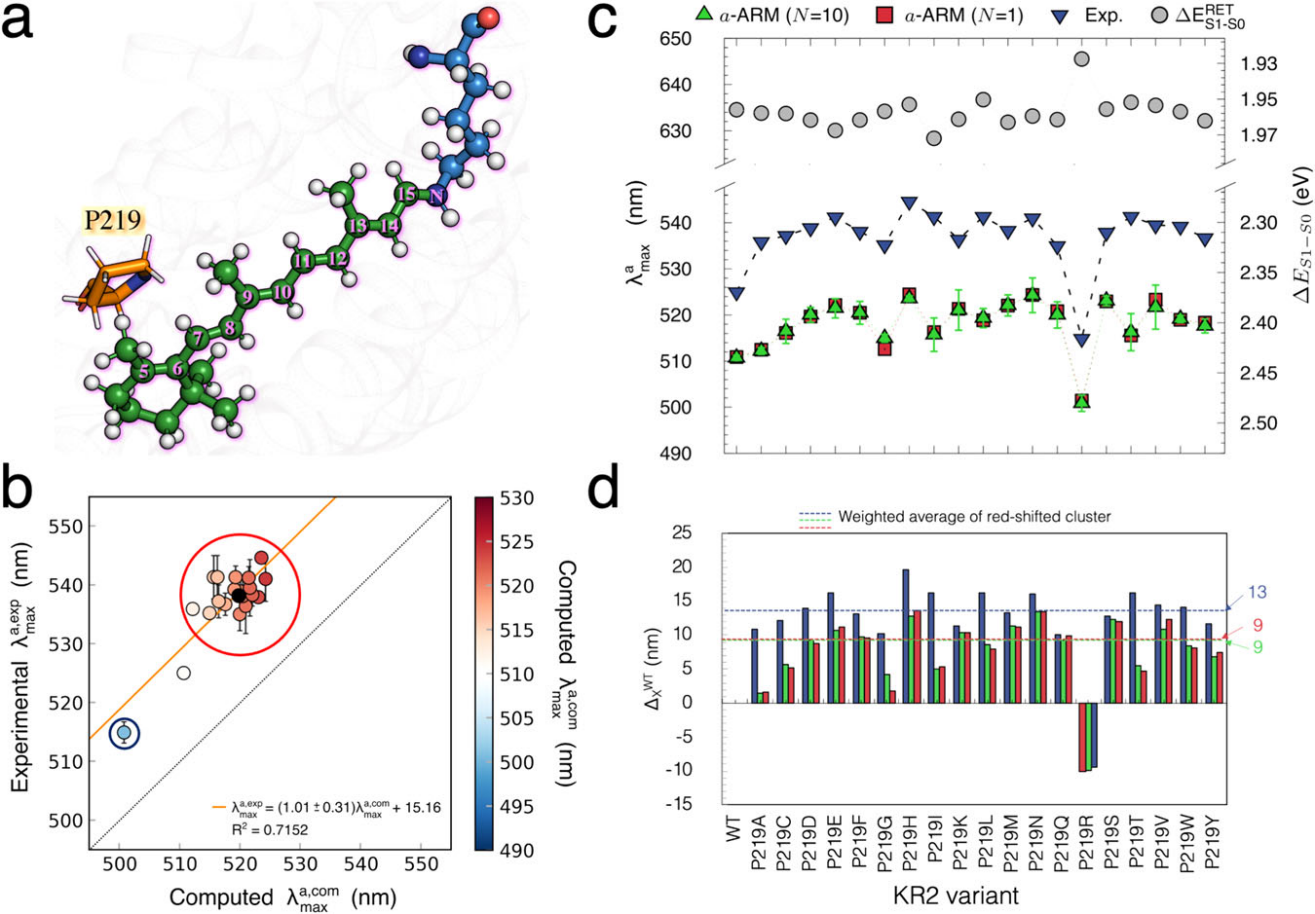
Computed vs experimental trend in maximum absorption wavelength. (**a**) Scheme of the retinal proton Schiff base of WT- KR2. The residue P219 is located near the β-ionone ring. (**b**) Correlation plot between computed $(\varDelta {{{{{{\rm{E}}}}}}}_{{{{{{\rm{S1}}}}}}-{{{{{\rm{S0}}}}}}}^{{{{{{\rm{a}}}}}}-{{{{{\rm{ARM}}}}}}})$ and measured $(\varDelta {{{{{{\rm{E}}}}}}}_{{{{{{\rm{S1}}}}}}-{{{{{\rm{S0}}}}}}}^{{{{{\mathrm{Exp}}}}}})$ vertical excitation energy for WT-KR2 rhodopsin and 19 of its mutants (P219X, with X = A, C, D, E, F, G, H, I, K, L, M, N, Q, R, S, T, V, W, Y). The red circle indicates the red-shifted mutant cluster whose weighted average value is marked with a black circle. The blue circle indicates the only blue-shifted mutant of the set. (**c**) Maximum absorption wavelength (λ^a^_max_, nm) and (ΔE^a−ARM^_S1-S0_, eV) for wild-type KR2 and 19 of its P219X variants. The experimental values (blue down triangles) are presented along with the *a*-ARM values predicted by using the average of *n* = 10 replicas (green triangles), and the representative replica with ΔE^a-ARM^_S1-S0_ closest to the average (red squares). The values for the retinal chromophore (gray circles) calculated in vacuum (ΔE^RET^_S1-S0_), outside the protein environment, are also presented. Row data is provided in Supplementary Table 2. (**d**) Relative observed and computed ΔE^a-ARM^_S1-S0_ changes (Δ,_X_^WT^) with respect to WT. Row data is provided in Supplementary Table 3. The dashed lines show the values corresponding the weighted averages of the red-shifted cluster. S_0_ and S_1_ energy calculations were performed at the CASPT2(12,12)//CASSCF(12,12)/AMBER level of theory using the 6-31G(d) basis set.

The achieved WT-KR2 model shows that P219 is a suitable position for color tuning. Indeed, as displayed in Fig. 5a, it is close to the β-ionone ring of rPSBAT, so it is expected that the replacement of Pro by residues with different steric hindrance and/or polarity can affect the vertical excitation energy by stabilizing or destabilizing S_1_ with respect to S_0,_ as has been previously observed by some of the authors when modeling the mutants P219G and P219T using the *original* version of the ARM protocol^22^. Although the conclusions derived from the study of Inoue et. al. can be qualitatively compared with the results presented in this work (see Supplementary Fig. 4 and Supplementary Table 4), notice that they cannot be quantitatively compared since (i) the QM/MM models were constructed from a different X-ray structure (i.e., 3X3C), using the *original* ARM^9^ that featured (ii) manual input file generation (i.e., handmade and not reproducible counterion placement, different chromophore cavity, etc.) and (iii) a different methodological approach for the generation of the mutant side-chain, as described in Supplementary Note 5.

We begin the analysis of the experimental data by comparing the $\varDelta {{{{{{\rm{E}}}}}}}_{{{{{{\rm{S1}}}}}}-{{{{{\rm{S0}}}}}}}^{{{{{\mathrm{Exp}}}}}}$ trend with the corresponding computed values (see Supplementary Table 4), in the hope to learn how the P219X mutants shift their colors with respect to WT-KR2 (see blue down-triangles in Fig. 5c). As shown in Fig. 5b and reported in Table 1, the observed values vary from 545 nm to 515 nm. Remarkably, as reported in Supplementary Table 4, the P219R is the only variant that exhibit a blue-shifted effect with respect to the WT-KR2 of ca. 1.1 kcal mol^−1^, whereas the other 18 mutants exhibit a red-shifting effect raging from −1.0 to −2.0 kcal mol^−1^ suggesting that an electrostatic destabilization of the chromophore positive charge near its β-ionone is a key factor in the observed color tuning.

It is apparent that the WT-KR2 and P219R define a correlation line which is parallel to the perfect correlation line between observed and computed values, thus indicating a general systematic blue shifted error of ca. 15 nm (see Fig. 5d). On the other hand, the <1.0 kcal mol^−1^ observed $\varDelta {{{{{{\rm{E}}}}}}}_{{{{{{\rm{S1}}}}}}-{{{{{\rm{S0}}}}}}}^{{{{{\mathrm{Exp}}}}}}$ variations among the 18 red-shifted mutants are, in most cases, far too small for their trend to be reproduced by a *a*-ARM QM/MM model. These mutants form a cluster whose weighted average (the black circle in Fig. 5b which represents the average of the measured and computed values weighted according to the more frequent deviation from the wild-type value, see Supplementary Note 6 and Supplementary Note 7) aligns with WT-KR2 and P219R supporting the general validity of the constructed models and confirming the presence of a systematic error (the row data reported in Table 1shows a systematic blue-shifted error of ca. 2.0 kcal mol^−1^). Notice that for these models the mean absolute error (MAE) and the mean absolute deviation (MAD), calculated as indicated in Supplementary Note 6, are 1.9 and 0.3 kcal mol^−1^ respectively, consistently with those established for the *a*-ARM protocol^9,30,35–37^. For this reason, in the present work we avoid a detailed analysis of the members of the red-shifted cluster and focus on their weighted average features. In other words, we primarily focus on the effects that make: i) the P219R and ii) the center of the red-shifted cluster different from WT-KR2 in terms of $\varDelta {{{{{{\rm{E}}}}}}}_{{{{{{\rm{S1}}}}}}-{{{{{\rm{S0}}}}}}}^{a-{{{{{\rm{ARM}}}}}}}$.

The observed $\varDelta {{{{{{\rm{E}}}}}}}_{{{{{{\rm{S1}}}}}}-{{{{{\rm{S0}}}}}}}^{{{{{\mathrm{Exp}}}}}}$ and calculated $\varDelta {{{{{{\rm{E}}}}}}}_{{{{{{\rm{S1}}}}}}-{{{{{\rm{S0}}}}}}}^{a-{{{{{\rm{ARM}}}}}}}$ values are reported in Fig. 5c, while their difference relative to WT-KR2 are given in Fig. 5d (see also Supplementary Table 5). These quantities were computed as the difference between observed $\varDelta {{{{{{\rm{E}}}}}}}_{{{{{{\rm{S1}}}}}}-{{{{{\rm{S0}}}}}}}^{{{{{\mathrm{Exp}}}}}}$for each P219X mutant with respect to the observed value of the WT-KR2 (Δ_max,X_^WT,Exp^), as well as the difference between computed $\varDelta {{{{{{\rm{E}}}}}}}_{{{{{{\rm{S1}}}}}}-{{{{{\rm{S0}}}}}}}^{a-{{{{{\rm{ARM}}}}}}}$ of each of the P219X mutants with respect to the corresponding WT-KR2 (Δ_max,X_^WT,a-ARM^) values. Notice that in this figure the average values (see dashed horizontal lines) for the red-shifted cluster are also provided. In all the cases, blue or red direction of the shift is reproduced. More interestingly, in Fig. 5d it is evident that, in line with the observations (blue bars), the computed data (green and red bars) show that P219R is the only mutant presenting a blue-shifted effect and P219H is the most red-shifted mutant.

In order to quantify the parallelism between the computed and experimental trends in excitation energy (see Supplementary Note 7), we have calculated the trend deviation $(\Vert {{{{{\rm{Trend}}}}}}\,{{{{{\rm{Dev}}}}}}.\Vert =|{{\varDelta }_{{{{{{\rm{max }}}}}},{{{{{\rm{X}}}}}}}}^{{{{{{\rm{WT}}}}}},{{{{\mathrm{Exp}}}}}}-{{\varDelta }_{{{{{{\rm{max }}}}}},{{{{{\rm{X}}}}}}}}^{{{{{{\rm{WT}}}}}},{{{{{\rm{a}}}}}}-{{{{{\rm{ARM}}}}}}}|)$ as 0.4 ± 0.3 kcal mol^−1^ (0.02 ± 0.01 eV), using the data reported in Supplementary Table 3. Such value is close to the value reported for the *a*-ARM protocol in ref. ^9^. (see “Materials and methods” section), further supporting the general validity of our QM/MM models.

The results above indicate that the 20 generated QM/MM models may be employed to investigate the color tuning mechanism operating in KR2. This is done by analyzing the differences between the P219R model and the center of the red-shifted cluster (18 red-shifted models) with respect to the WT-KR2 model. A first question to be answered is: why R is the only residue, out of the three canonical positively charged residue (K, H and R), causing a blue-shift in spite of its not dramatically larger volume?

### Color tuning analysis in terms of steric and electrostatic effects

**Fig. 6:**
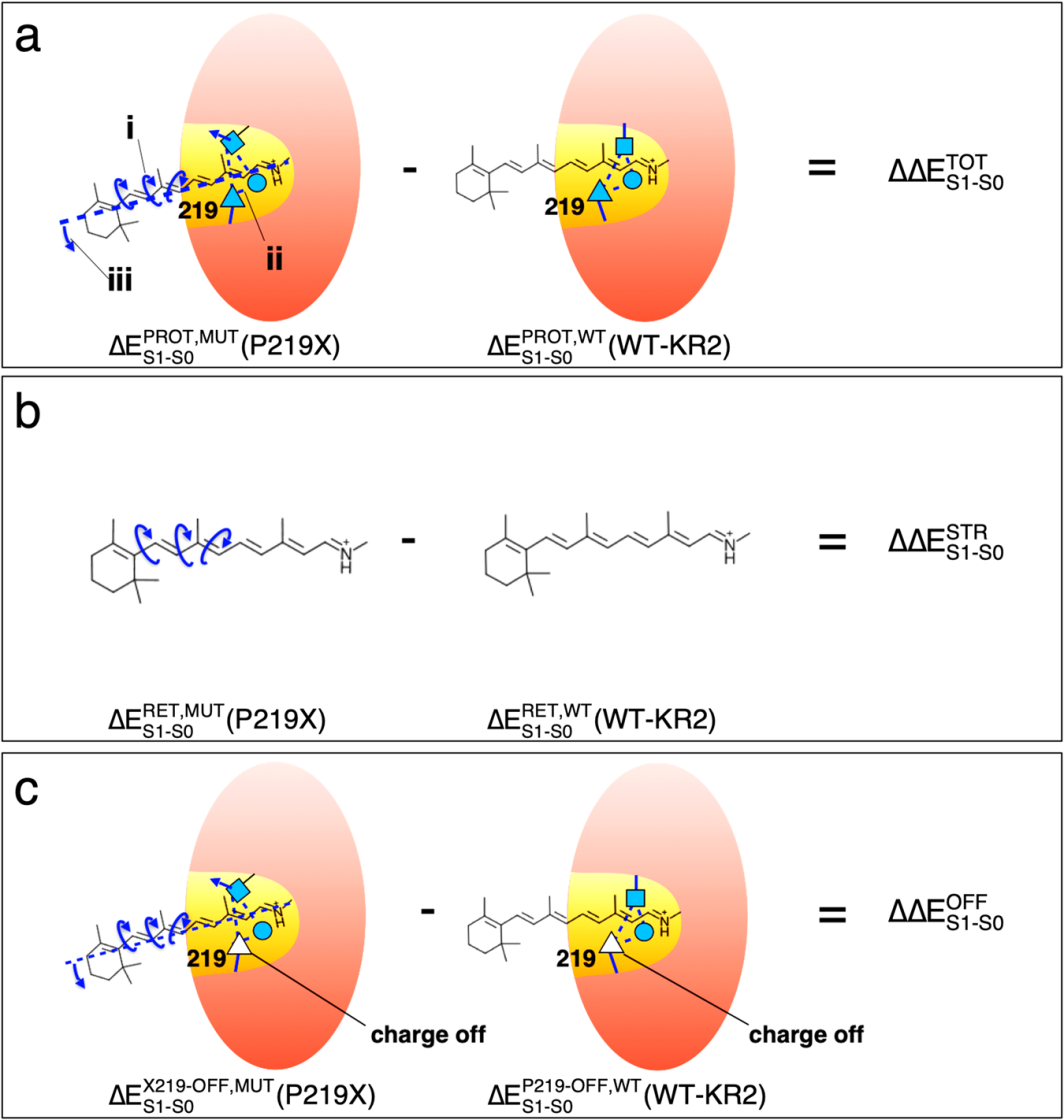
Pictorial illustration of the corresponding excitation energy analysis terms. (**a**) Difference between the *a*-ARM models excitation energies (in protein environment, ΔE^PROT^_S1-S0_ = $\varDelta {{{{{{\rm{E}}}}}}}_{{{{{{\rm{S1}}}}}}-{{{{{\rm{S0}}}}}}}^{{{{{{\rm{a-ARM}}}}}}}$) of a P219X mutant with respect to the wild-type due to (i) structural deformation (see curly arrows) and (ii) structural deformation of the cavity residues (see straight arrow and dotted lines indicating the hydrogen bond network) and (iii) rPSBAT reorientation (see dashed line along the chromophore axis). (**b**) Difference between the excitation energies of the chromophores (in vacuum, ΔΔE^STR^_S1-S0_) of P219X mutant and wild-type, isolated at their equilibrium structures from the protein environment. (**c**) Difference between the excitation energies (in protein environment) of a P219X mutant with respect to the wild-type (i.e., same as in part (**a**)) but calculated after setting to zero the charges of residue X in the mutant and the charges of residue P in the wild-type. Red ovals and yellow areas represent the protein environment and the chromophore cavity, respectively.

In order to gain insight into the color tuning mechanism inducing $\varDelta {{{{{{\rm{E}}}}}}}_{{{{{{\rm{S1}}}}}}-{{{{{\rm{S0}}}}}}}^{a-{{{{{\rm{ARM}}}}}}}$red- and blue-shifting, we looked at the steric and/or electrostatic effects that modulate the energy of either the S_0_ or the S_1_ states and, consequently, the excitation energy. As mentioned above, such analyses are primarily focused on the red-shifted (18 mutants) cluster center and P219R mutant (see Fig. 5b). In Fig. 6, we give a visual representation of three fundamental quantities (ΔΔE^TOT^_S1–S0_, ΔΔE^STR^_S1–S0_, and ΔΔE^X219-OFF^_S1–S0_) whose values are a function of either the structural (both at the chromophore and protein cavity levels) or electrostatic changes of each mutant with respect to WT-KR2. ΔΔE^TOT^_S1–S0_ is the “Total” excitation energy change (see Fig. 6a). It is directly computed as the difference between the QM/MM computed vertical excitation energies (see red squares in Fig. 5c) of the mutant and WT-KR2 protein models. ΔΔE^STR^_S1–S0_ is the “Steric component” of ΔΔE^TOT^_S1–S0_ (see Fig. 6b). It is directly computed as the difference between the QM/MM vertical excitation energies of the isolated retinal chromophores, taken with their protein environment geometries, (see gray circles in Fig. 5c) of the mutant and WT-KR2. As we will see in the following, using these quantities we can compute three additional components. ΔΔE^ELE(t)^_S1–S0_ is the “Total electrostatic component” that is indirectly computed as the difference between the Total and the Steric components above (ΔΔE^ELE(t)^_S1–S0_ = ΔΔE^TOT^_S1–S0_ − ΔΔE^STR^_S1–S0_) for each mutant. As specified below, ΔΔE^ELE(t)^_S1–S0_ can be decomposed into two parts. ΔΔE^ELE(i)^_S1–S0_ is the “Indirect electrostatic component” that is indirectly computed in two steps by first computing the differences between the vertical excitation energy of the mutant and WT-KR2 obtained after having switched off (turned to zero) the charges of residue 219 (see Fig. 6c), component ΔΔE^OFF^_S1–S0_) and then by subtracting from such difference the steric effect ${\Delta \Delta {{{{{\rm{E}}}}}}}_{{{{{{\rm{S1}}}}}}-{{{{{\rm{S0}}}}}}}^{{{{{{\rm{STR}}}}}}}$ defined above. Finally, ΔΔE^ELE(d)^_S1–S0_ is the “Direct electrostatic component” that is indirectly computed as ΔΔE^ELE(d)^_S1–S0_ = ΔΔE^ELE(t)^_S1–S0_ − ΔΔE^ELE(i)^_S1–S0_. Further details are provided in Supplementary Note 8 (see Supplementary Table 6 and Supplementary Table 7).

### Steric effects

**Fig. 7:**
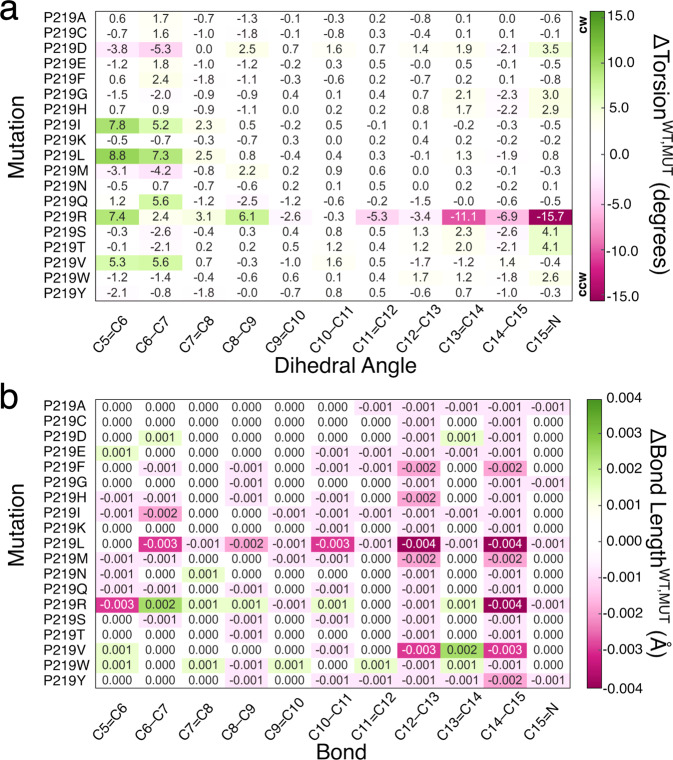
Heatmap representation of the variation of structural parameters of P219X (X= A, C, D, E, F, G, H, I, K, L, M, N, Q, R, S, T, V, W, Y) mutants with respect to the wild-type KR2 (WT-KR2). Difference between WT-KR2 and mutant (**a**) dihedral angles, $\varDelta {{{{{{\rm{Torsion}}}}}}}^{{{{{{\boldsymbol{WT}}}}}},{{{{{\boldsymbol{MUT}}}}}}}$ and (**b**) bond lengths, $\varDelta {{{{{{\rm{Bond}}}}}}{{{{{\rm{Length}}}}}}}^{{{{{{\boldsymbol{WT}}}}}},{{{{{\boldsymbol{MUT}}}}}}}$. Counterclockwise (CCW) and clockwise (CW) orientation of the rPSBAT.

We begin the discussion on steric effects (here by “steric effects” we mean “indirect” or “geometrical” effects, i.e., the change in excitation energy of a chromophore due to a change in the minimum geometry. The change in geometry could be induced by both steric and electrostatic factors) by investigating how the retinal chromophore is structurally modified by the mutations (i) near the β-ionone ring and (ii) near the Schiff base linkage (see Fig. 5a). As discussed below, such structural rearrangements of rPSBAT rather than being a simple effect induced by the side-chain replacement, could be also attributed to a different charge distribution due to changes in protonation states for ionizable residues as well as water addition/removal. Notice that we evaluated steric effect through an “atomistic” approach focused on the changes in rPSBAT geometrical and electronic structure and therefore not directly related to steric effects evaluated on the basis of the changes in residue volume addressed above. To do so, we select a representative QM/MM structure for each mutant model (i.e., the replica with $\varDelta {{{{{{\rm{E}}}}}}}_{{{{{{\rm{S1}}}}}}-{{{{{\rm{S0}}}}}}}^{{{{{{\rm{a}}}}}}-{{{{{\rm{ARM}}}}}}}$ closest to the average, see red squares in Fig. 5c) and compute the difference between the magnitude of its most relevant structural parameters (e.g., torsional dihedral angles and bond lengths) with respect to those corresponding to the representative structure of the WT-KR2 model. A visual structural comparation between each mutant and WT-KR2 is provided in Supplementary Figure 5. In Fig. 7 we report a heatmap visualization of such differences in terms of skeletal dihedral angles ($\varDelta {{{{{{\rm{Torsion}}}}}}}^{WT,MUT}$, Fig. 7a) and carbon-carbon bond lengths ($\varDelta {{{{{{\rm{Bond}}}}}}{{{{{\rm{Length}}}}}}}^{WT,MUT}$, Fig. 7b). Based on the range in which the latest quantities change, we establish an arbitrary threshold of 2.0 degrees and 0.01 Å, respectively, as meaningful variations. The dihedrals with the largest variation are C5 = C6 and C6-C7 that, as shown in Fig. 5a, belong to the rPSBAT framework geometrically closer to the 219 residue. In addition, for specific cases the “reactive” C13 = C14 dihedral as well as the C14-C15 and C15 = N dihedrals show variability. We start the analysis discussing the variants belonging to the red-shifted cluster. These are almost constantly accompanied by structural effect near the β-ionone ring (among them, P219I, P219L, P219M, P219V, P219F, P219Y featuring hydrophobic side-chain, and P219Q featuring large polar uncharged side-chain present the largest structural effects) making such a structural deformation a characteristic of the cluster center. On the other hand, P219G, P219H and P219W show a limited change in the C15 = N region and P219D (in its neutral form), P219S and P219T (i.e., polar uncharged side-chains) present changes in both regions. As expected, the blue-shifted P219R mutant featuring a positively charged side-chain, induces a significant, more than 2.0 degrees, change of its dihedral angles near the β-ionone ring. More interestingly, this mutant exhibits a particular large variation of the “reactive” C13 = C14 dihedral as well as the C14-C15 and C15 = N dihedrals (see Fig. 7a).

Although at a first glance none of the bond lengths are significantly altered with respect to the WT-KR2 reference (see Fig. 7b), it is well-known that the excitation energy of a conjugated chromophore in its protein environment is sensitive to the delocalization of the π-electron and, consequently, to bond length alternation (BLA)^38^. The latter is computed as the difference between the average single bond length and the average double bond length of the π-conjugated chromophore^39,40^. Therefore, we computed the BLA for each of the P219X variants as well as their difference with respect to the WT-KR2. The results, reported in Supplementary Table 9 and Supplementary Note 11, show that the 18 variants of the red-shifted cluster present values of BLA lower than the WT-KR2, indicating that this is a common feature shared by the red-shifted cluster and its center. In contrast, the only blue-shifted variant presents a larger BLA value. Such results are consistent with a previous study reported for some of the authors, where it is discussed that more conjugation of the double bonds implies less BLA and red-shift^22^.

**Fig. 8:**
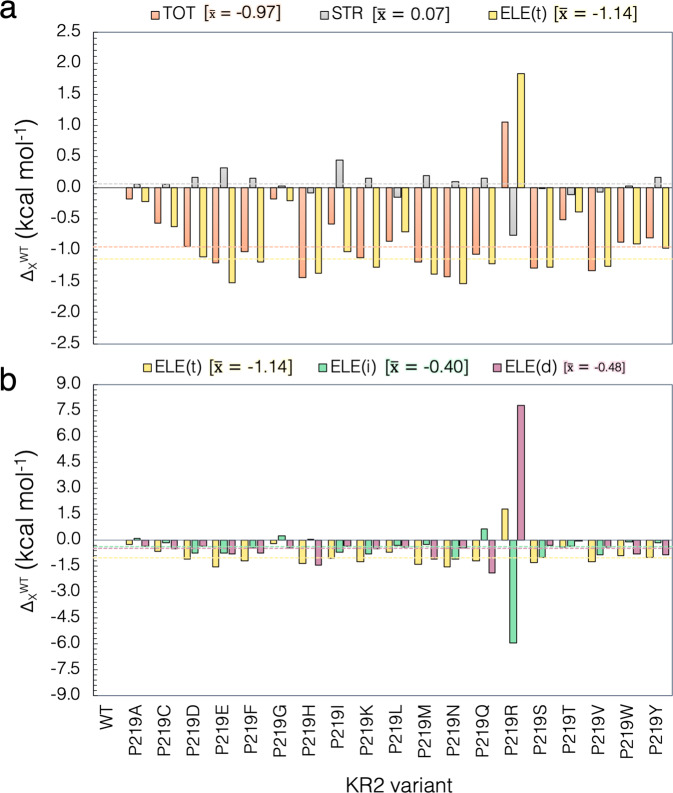
Steric and electrostatic contributions to the vertical excitation energy. (**a**) Total steric (STR) and electrostatic (ELE(t)) contributions of the interaction of the retinal with the protein environment for the P219X (X = A, C, D, E, F, G, H, I, K, L, M, N, Q, R, S, T, V, W, Y). (**b**) Decomposition of the total ELE(t) electrostatic effects on its indirect (ELE(i)) and direct (ELE(d)) components. Row data is provided in Supplementary Table 5. The dashed lines and corresponding numerical values refer to the weighted average values $(\bar{{{{{{\boldsymbol{x}}}}}}})$ of the 18 residues of the red-shifted cluster exclusively, presented in square parenthesis.

We now look at the impact of the described geometrical changes on the λ^a^_max_ value. To this aim, we compare the computed $\varDelta {{{{{{\rm{E}}}}}}}_{{{{{{\rm{S1}}}}}}-{{{{{\rm{S0}}}}}}}^{{{{{{\rm{RET}}}}}},{{{{{\rm{MUT}}}}}}}$ values with the corresponding wild-type $\varDelta {{{{{{\rm{E}}}}}}}_{{{{{{\rm{S1}}}}}}-{{{{{\rm{S0}}}}}}}^{{{{{{\rm{RET}}}}}},{{{{{\rm{WT}}}}}}}$ for the isolated chromophore (in kcal mol^−1^), as reported in Supplementary Table 7. This analysis consists in extracting, for WT-KR2 and for each variant, the rPSBAT structure from the protein and computing its vertical excitation energy without relaxing the chromophore structure (see Fig. 6b). A small ${\Delta \Delta {{{{{\rm{E}}}}}}}_{{{{{{\rm{S1}}}}}}-{{{{{\rm{S0}}}}}}}^{{{{{{\rm{STR}}}}}}}=\varDelta {{{{{{\rm{E}}}}}}}_{{{{{{\rm{S1}}}}}}-{{{{{\rm{S0}}}}}}}^{{{{{{\rm{RET}}}}}},{{{{{\rm{MUT}}}}}}}-\varDelta {{{{{{\rm{E}}}}}}}_{{{{{{\rm{S1}}}}}}-{{{{{\rm{S0}}}}}}}^{{{{{{\rm{RET}}}}}},{{{{{\rm{WT}}}}}}}$ value indicates that the geometrical distortion of the retinal due to the point mutation has only a limited effect on the excitation energy change. As mentioned above, we analyze the ${\Delta \Delta {{{{{\rm{E}}}}}}}_{{{{{{\rm{S1}}}}}}-{{{{{\rm{S0}}}}}}}^{{{{{{\rm{STR}}}}}}}$ contribution for the red-shifted cluster center and P219R. To this aim, we computed the weighted average of the ${\Delta \Delta {{{{{\rm{E}}}}}}}_{{{{{{\rm{S1}}}}}}-{{{{{\rm{S0}}}}}}}^{{{{{{\rm{STR}}}}}}}$ contribution for the members of the red-shifted cluster (see gray dashed line in Fig. 8a) and compare this value with the ${\Delta \Delta {{{{{\rm{E}}}}}}}_{{{{{{\rm{S1}}}}}}-{{{{{\rm{S0}}}}}}}^{{{{{{\rm{STR}}}}}}}$ contribution of the blue-shifted one. Consistently with the small variations in $\varDelta {{{{{{\rm{E}}}}}}}_{{{{{{\rm{S1}}}}}}-{{{{{\rm{S0}}}}}}}^{{{{{{\rm{RET}}}}}},{{{{{\rm{WT}}}}}}}$ reported in Fig. 5c (see gray circles) for the red-shifted cluster, the weighted ${\Delta \Delta {{{{{\rm{E}}}}}}}_{{{{{{\rm{S1}}}}}}-{{{{{\rm{S0}}}}}}}^{{{{{{\rm{STR}}}}}}}$ average of about 0.07 kcal mol^−1^ reported in Fig. 8a and Supplementary Table 6, show that the impact of the rPSBAT geometrical deformation on the change in their excitation energy is very limited. On the other hand, as also confirmed by the geometrical data discussed above, the blue-shifted P219R variant features a considerable ${\Delta \Delta {{{{{\rm{E}}}}}}}_{{{{{{\rm{S1}}}}}}-{{{{{\rm{S0}}}}}}}^{{{{{{\rm{STR}}}}}}}$ contribution of −0.8 kcal mol^−1^. These results, also reported in Supplementary Table 7, give a first indication about the different behavior on the color tuning mechanism exhibited for the red-shifted clusters and P219R mutant.

### Electrostatic effects

The QM/MM models also allow to investigate electrostatic effects. As anticipated above, the total electrostatic effect (ΔΔE^ELE(t)^_S1–S0_) can be decomposed in two parts: (i) the first can be considered as a *direct* component (ΔΔE^ELE(d)^_S1–S0_) due to the variation in number, magnitude, and position of the point charges of residue 219 caused by the P to X replacement and (ii) a more *indirect* component (ΔΔE^ELE(i)^_S1–S0_) produced from the reorganization of the local environment and hydrogen bond network induced by the same replacement and due to the fact that conserved residues and water molecules change in position or orientation. Moreover, as discussed below, possible changes in protonation states of conserved residues, induced by P to X replacement, have a major contribution to the *indirect* component.

Figure 8a presents the relative vertical excitation energy for each mutant with respect to WT-KR2 value $({\Delta \Delta {{{{{\rm{E}}}}}}}_{{{{{{\rm{S1}}}}}}-{{{{{\rm{S0}}}}}}}^{{{{{{\rm{TOT}}}}}}})$, and its steric $({\Delta \Delta {{{{{\rm{E}}}}}}}_{{{{{{\rm{S1}}}}}}-{{{{{\rm{S0}}}}}}}^{{{{{{\rm{STR}}}}}}})$ and total electrostatic $({\Delta \Delta {{{{{\rm{E}}}}}}}_{{{{{{\rm{S1}}}}}}-{{{{{\rm{S0}}}}}}}^{{{{{{\rm{ELE}}}}}}({{{{{\rm{t}}}}}})})$ contributions (see Supplementary Note 8). Again, it is apparent that steric effects due to the variation in geometry of the retinal chromophore (see gray bars in Fig. 8a), are limited and do not compete with the electrostatic effect, except for the case of the blue-shifted P219R. In general, it can be claimed that, on the basis of the *a*-ARM QM/MM models the electrostatic effect is the one driving the changes in the computed and, therefore, observed λ_max_ values. Accordingly, in each case ${\Delta \Delta {{{{{\rm{E}}}}}}}_{{{{{{\rm{S1}}}}}}-{{{{{\rm{S0}}}}}}}^{{{{{{\rm{ELE}}}}}}({{{{{\rm{t}}}}}})}$ (see gold bars in Fig. 8a) follows the trend of ${\Delta \Delta {{{{{\rm{E}}}}}}}_{{{{{{\rm{S1}}}}}}-{{{{{\rm{S0}}}}}}}^{{{{{{\rm{TOT}}}}}}}$, being a positive value for the cases that present a red-shifting effect, and a negative value in the only blue-shifted case.

**Fig. 9:**
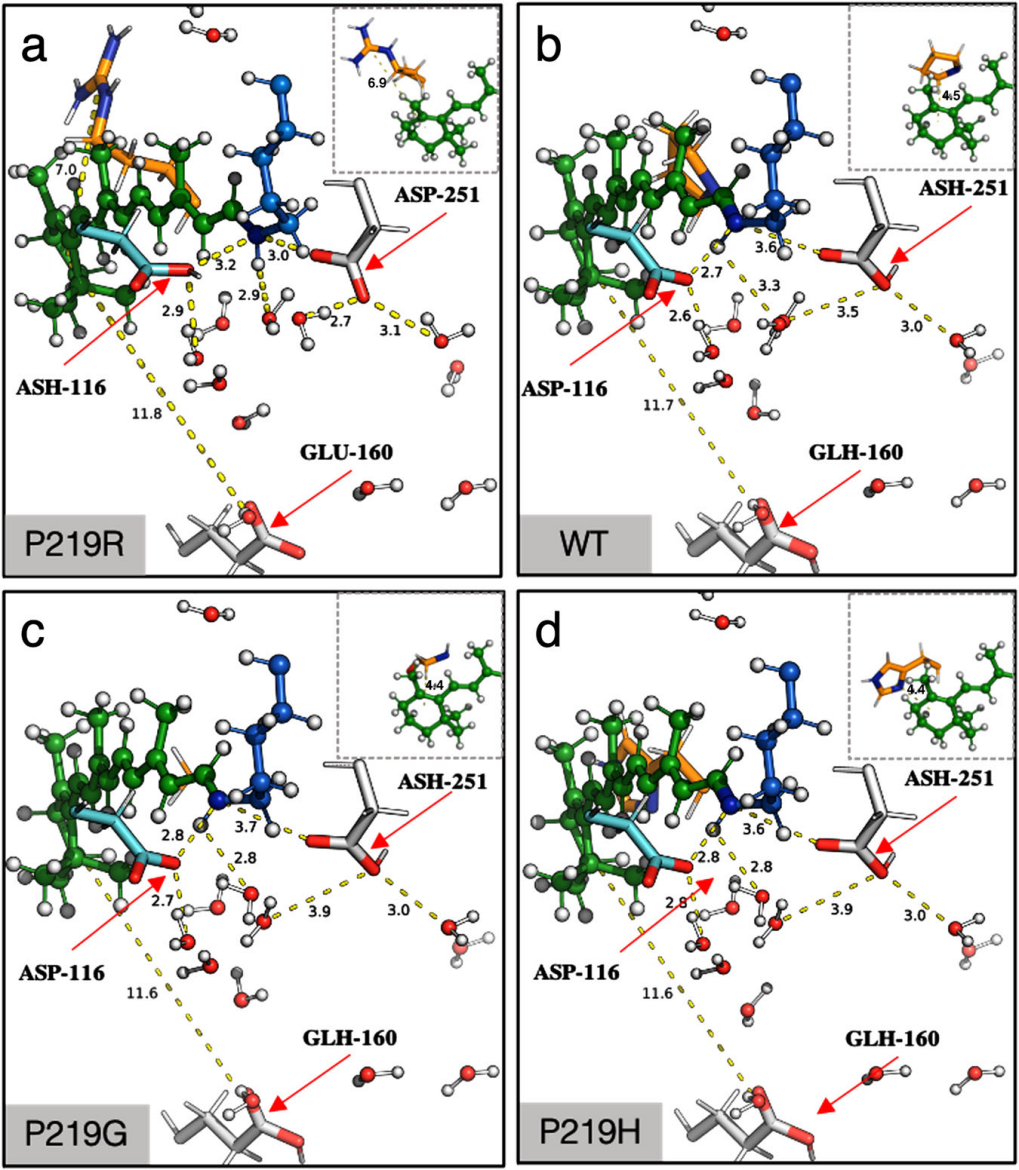
*a*-ARM QM/MM-optimized models for P219R, WT-KR2, P219G and P219H. Differences between Hydrogen Bond Network (HBN) presented for (**a**) the only blue-shifted variant (P219R), (**b**) the wild-type (WT-KR2), (**c**) the least red-shifted variant (P219G) and (**d**) the most red-shifted variant (P219H). Hydrogen bonds are represented as dashed lines. The red arrows indicate the protonation state of the relevant residues.

We now discuss the role of the two components of the electrostatic effects that are calculated using the definitions given above (see Fig. 8b and Supplementary Note 8). A comparison of the contribution of both direct and indirect electrostatic components is plotted in Fig. 8b and the values are reported in Supplementary Table 7. Again, we performed the analyses for both $\varDelta {{{{{{\rm{E}}}}}}}_{{{{{{\rm{S1}}}}}}-{{{{{\rm{S0}}}}}}}^{{{{{{\rm{ELE}}}}}}({{{{{\rm{d}}}}}})}$ and $\varDelta {{{{{{\rm{E}}}}}}}_{{{{{{\rm{S1}}}}}}-{{{{{\rm{S0}}}}}}}^{{{{{{\rm{ELE}}}}}}({{{{{\rm{i}}}}}})}$ components in terms of weighted average value for the red-shifted cluster and value of the blue-shifted P219R. As observed, the direct $\varDelta {{{{{{\rm{E}}}}}}}_{{{{{{\rm{S1}}}}}}-{{{{{\rm{S0}}}}}}}^{{{{{{\rm{ELE}}}}}}({{{{{\rm{d}}}}}})}$ component of the red-shifted cluster is of about −0.48 kcal mol^−1^, while the blue-shifted P219R mutant displays a large value of 7.8 kcal mol^−1^. The latter value is attributed to the disappearance of a S_1_ destabilizing Arg positive charge at position 219. The differences in both sign and magnitude of the $\varDelta {{{{{{\rm{E}}}}}}}_{{{{{{\rm{S1}}}}}}-{{{{{\rm{S0}}}}}}}^{{{{{{\rm{ELE}}}}}}({{{{{\rm{d}}}}}})}$ in both clusters, show how they operate in a different fashion. Furthermore, the analysis of the indirect $\varDelta {{{{{{\rm{E}}}}}}}_{{{{{{\rm{S1}}}}}}-{{{{{\rm{S0}}}}}}}^{{{{{{\rm{ELE}}}}}}({{{{{\rm{i}}}}}})}$ component shows that the weighted average of the red-shifted cluster of about −0.40 kcal mol^−1^ is only comparable in sign but not in magnitude with the value of the blue-shifted variant, computed as −6.0 kcal mol^−1^. Such effect is expected since the blue-shifted P219R is the only variant that exhibits a considerable change in the factors defined above as the cause of the indirect electrostatic component. More specifically, the QM/MM model of P219R suggests that when full positive charge is introduced, the general structure gets naturally counterbalance by the deprotonation of the Glu residue in position 160 even if this residue is located ca. 11 Å away from the β-ionone ring (see Fig. 9a). This change, that justifies the large value of the indirect electrostatic component, is also accompanied by a substantial rearrangement of the molecules of water near the chromophore protonated Schiff base group and therefore a variation of the hydrogen bond network. Furthermore, the same model indicates that, in contrast to WT-KR2 and the red-shifted cluster, the P219R model can only reproduce the observed λ_max_ value after changing the protonation states of the rPSBAT counterion complex making D116 neutral and D251 negatively charged (see Fig. 9a). We hypothesize that this reorganization of the protonation states is connected to the observed lack of sodium pumping activity in this mutant. This relatively important alterations appear reasonable when considering, as also stressed above, that a full localized charge is introduced in the KR2 cavity upon mutation. In fact, such large changes, including the changes in protonation states, are found to be not necessary in the mutant models P219K and P219H (see Fig. 9d) as the residues Lys and His are in their deprotonated (i.e., neutral) forms and, thus, the general arrangement of the charges in the cavity is not altered with respect to WT-KR2 (see Fig. 9b) or P219G (see Fig. 9c).

## Conclusions

Above we have presented a combined experimental and computational investigation of the P219X mutants of KR2 supporting the existence of a G/P switch in microbial rhodopsins. We have also reported on a computational analysis of the mutant vertical excitation energy indicating that G/P is, essentially, an electrostatic switch capable of a ca. 30 nm modulation (going from R to H). More specifically, the decomposition of the simulated excitation energy changes using multiconfigurational quantum chemistry-based QM/MM models, suggests that the switch operates by inducing variations in the electrostatic interaction of the chromophore with its environment while replacement-induced geometrical distortions only contribute to the P219R blue-shifted variant.

The reported QM/MM modeling studies provide a mechanistic interpretation of the G/P switch color tuning mechanism. In fact, it is found that P219R features an increased planarity of the β-ionone and Schiff base moieties leading to a red-shifting steric $(\varDelta {{{{{{\rm{E}}}}}}}_{{{{{{\rm{S1}}}}}}-{{{{{\rm{S0}}}}}}}^{{{{{{\rm{STR}}}}}}})$ contribution of ca. −0.8 kcal mol^−1^. Such steric red-shifting effect, which is the result not only of the P/R replacement side-chain but also of a change in the HBN pattern, is counterbalanced by a larger blue-shifting electrostatic contribution $(\varDelta {{{{{{\rm{E}}}}}}}_{{{{{{\rm{S1}}}}}}-{{{{{\rm{S0}}}}}}}^{{{{{{\rm{ELE}}}}}}({{{{{\rm{t}}}}}})})$. The decomposition of $\varDelta {{{{{{\rm{E}}}}}}}_{{{{{{\rm{S1}}}}}}-{{{{{\rm{S0}}}}}}}^{{{{{{\rm{ELE}}}}}}({{{{{\rm{t}}}}}})}$ into direct $(\varDelta {{{{{{\rm{E}}}}}}}_{{{{{{\rm{S1}}}}}}-{{{{{\rm{S0}}}}}}}^{{{{{{\rm{ELE}}}}}}({{{{{\rm{d}}}}}})})$ and indirect $(\varDelta {{{{{{\rm{E}}}}}}}_{{{{{{\rm{S1}}}}}}-{{{{{\rm{S0}}}}}}}^{{{{{{\rm{ELE}}}}}}({{{{{\rm{i}}}}}})})$ components in P219R, reveals that: (i) $\varDelta {{{{{{\rm{E}}}}}}}_{{{{{{\rm{S1}}}}}}-{{{{{\rm{S0}}}}}}}^{{{{{{\rm{ELE}}}}}}({{{{{\rm{d}}}}}})}$ is significantly larger than in the other variants, inducing a large blue-shifting effect (ca. 7.8 kcal mol^−1^); (ii) $\varDelta {{{{{{\rm{E}}}}}}}_{{{{{{\rm{S1}}}}}}-{{{{{\rm{S0}}}}}}}^{{{{{{\rm{ELE}}}}}}({{{{{\rm{i}}}}}})}$ is almost of the same magnitude of $\varDelta {{{{{{\rm{E}}}}}}}_{{{{{{\rm{S1}}}}}}-{{{{{\rm{S0}}}}}}}^{{{{{{\rm{ELE}}}}}}({{{{{\rm{d}}}}}})}$ (ca. −6.0 kcal mol^−1^) but induces a red-shifting effect. These large and contrasting effects including structure, counterion and HBN pattern reorganization, are justified by the introduction of a positively charged arginine side-chain necessary to reproduce the observed P219R blue-shifting. No change in structure and counterion and only limited changes in HBN are instead seen in the members of the red-shifted cluster (i.e., when we represent it with a weighted average) or in the specific P219G and P219H variants representing the cluster minimum and maximum values. Indeed, all red-shifted variants conserve the main features of WT-KR2 and display a color tuning mechanism mainly controlled by the direct electrostatic changes associated with the replaced side-chain. These conclusions agree with those reported in Inoue et. al. the red-shifting mechanism of P219G and P219T was investigated.

The fact that in the investigated set of KR2 mutants both direct (the change in the electrostatic field due to the residue replacement) and indirect (the changes due to all other cavity reorganization induced by the replacement and including chromophore reorientation, side-chain and water relocations and the modification of the hydrogen bond network) electrostatic effects and specific changes in the chromophore structure contribute to determine the color variability, cast doubts on the possibility to extract simple rules for predicting how a switch operates without understanding the molecular-level details of the side chain replacement. In other words, the analysis indicates that the color tuning mechanism seen in KR2 has a complex origin.

The possibility to carry out, for the first-time, a systematic modeling of mutants (i.e., comprising all 19 possible replacements) underscores the importance of automated (or semi-automated) computational tools for the fast building of congruous (i.e. comparable) QM/MM models thus allowing comparative (i.e. trend) studies. More specifically, the employed *a*-ARM building protocol avoid errors and biases likely to impact the congruity of a generated QM/MM model set and, therefore, allows well defined comparative studies and mechanistic interpretations within the limits imposed by the model structure. Future work will be devoted to the study of other potential color switches present in the microbial rhodopsins with the hope to support the conclusions of the present work or document distinct types of color tuning mechanisms.

## Materials and methods

### Mutagenesis and protein expression

The synthesized genes of KR2 (GenBank Accession number: BAN14808.1) codon-optimized for *E. coli* were incorporated into the pET21a(+) vector (Novagen, Merck KGaA, Germany). The site-directed mutation was conducted using a QuikChange site-directed mutagenesis kit (Agilent, CA). The plasmids carrying the genes of the wild type of KR2 (WT-KR2), and mutants were transformed into the *E. coli* C43(DE3) strain (Lucigen, WI). The protein expression was induced with 1 mM isopropyl β-D-1-thiogalactopyranoside (IPTG) in the presence of 10 μM all-*trans* retinal for 4 h. See Supplementary Note 9 and Supplementary Table 8.

### Measurement of *λ*_max_ by hydroxylamine bleach

The *λ*_max_ of WT-KR2 and mutants was determined by bleaching the protein with hydroxylamine according to the previously reported method^22^. The *E. coli* cells expressing the rhodopsins were washed with a solution containing 100 mM NaCl, 50 mM Na_2_HPO_4_ (pH 7) for three times. The washed cells were treated with 1 mM lysozyme for 1 h at room temperature, and then disrupted by sonication. To solubilize the rhodopsins, 3% DDM was added, and the samples were stirred for overnight at 4 °C. The rhodopsins were bleached with 500 mM hydroxylamine and subjected to illumination of yellow light (λ > 500 nm) from the output of 1 kW tungsten−halogen projector lamp (Master HILUX-HR, Rikagaku) through a glass filter (Y-52, AGC Techno Glass, Japan). The absorption change upon the bleaching was measured by UV-visible spectrometer (V-730, JASCO, Japan).

### Ion-transport assay

The ion transport activity assay in *E. coli* cells was conducted according to the previously reported method^41^. The *E. coli* cells carrying the expressed rhodopsin were washed for three times and resuspended in unbuffered 100 mM NaCl or CsCl to assay the Na^+^ or H^+^ pump activity, respectively. The sample was illuminated after adjusting the pH to ~7 by the addition of a small amount of HCl or NaOH. The pH change upon light illumination was monitored with a pH electrode (9618S-10D, HORIBA, Japan). The wavelength of the illuminating light was changed by placing different color filters (Y-52, Y-54, O-55 and O-56, AGC Techno Glass, Japan) with a heat absorbing filter (HAF-50S-50H, SIGMAKOKI, Japan) in front of the light source (1 kW tungsten−halogen projector lamp, Master HILUX-HR, Rikagaku) for KR2 WT (λ > 500 nm), and P219X (λ > 520 nm) to correct for the change in the degrees of light absorption by their spectral shift.

### QM/MM modeling

Congruous and reproducible computational models for WT-KR2 and its P219X (X = A, C, D, E, F, G, H, I, K, L, M, N, Q, R, S, T, V, W, Y) mutants were generated using the *a*-ARM version^9^ of the Automatic Rhodopsin Modeling protocol^34^, a specialized user-friendly command-line oriented computational tool (i.e., Python3-based software package) for either the fully-automated (default) or semi-automated (i.e., customized) construction of basic hybrid QM/MM models of rhodopsins^30,35–37,42^, called *a*-ARM models. The produced *a*-ARM models illustrated in Fig. 4a are monomeric, “gas-phase”, and globally uncharged, based on electrostatic embedding and the hydrogen link-atom frontier between the QM and MM subsystems. The term “gas-phase” refers to the fact that the protein membrane and solvation effects are not explicitly modeled during the calculations. Nevertheless, the electrostatic effect of the protein external environment is effectively accounted for by an asymmetric distribution of external ions (i.e., Cl^−^ or Na^+^) whose charge and position are determined, automatically, by a specific algorithm, near the most positively and/or negatively charged surface amino acids in both the intracellular (IS) and extracellular (OS) protein surfaces. Moreover, to account the water-mediated hydrogen-bond network (HBN) in the protein cavity, the internal crystallographic waters are retained in the model, while the waters that are not experimentally detected are assumed to be extremely mobile or just absent in the chromophore hydrophobic protein cavity. Considering these approximations, during the construction of the *a*-ARM model the rhodopsin structure is subdivided into three sub-systems requiring layers of increasing level of accuracy for their description. The first MM-based sub-system, called protein environment, features residues (backbone and side-chain atoms) fixed at the crystallographic or comparative (homology) structure and incorporates external Cl^−^ and/or Na^+^ counterions fixed at preoptimized positions as well as some crystallographic/comparative waters (see below). The second sub-system, namely the chromophore cavity, constitutes the second MM layer of the model and contains amino acid residues with fixed backbone and relaxed side-chains, including the covalently linked lysine residue atoms (excluding the terminal NH_2_-C_ε_ moiety), as well as flexible crystallographic/comparative waters in the vicinity (<4 Å) of the rPSB. The third sub-system, called Lys-QM, contains the MM atoms of the covalently linked lysine side-chain in contact (through C_ε_) with the QM/MM frontier (i.e., 9 atoms) and the entire QM sub-system which corresponds to a N-methylated retinal chromophore (i.e., 53 atoms). As illustrated in Fig. 4b, c, to obtain the QM/MM model automatically the only required input is either the PDB ID or a template PDB file (X-ray structure or comparative model) of the rhodopsin, that is processed by two subsequent phases called input file generator (Phase I, Fig. 4b) and QM/MM model generator (Phase II, Fig. 4c) correspondingly, previously reported in refs. ^9,30,34,35^. and summarized in Supplementary Note 2 and Supplementary Note 3, respectively. Briefly, Phase II produces 10 model replicas of the *a*-ARM model via S_0_ geometry optimization of the input generated through Phase I starting from distinct molecular dynamics (MD) runs (see Supplementary Note 3). Such optimization relies on the use of the multi-configurational complete active space self-consistent field (CASSCF) at the 2-roots single-state, for modeling the QM sub-system while the MM subsystem is treated using the AMBER force field (CASSCF(12,12)/6-31 G(d)/AMBER level). The AMBER force field is not polarizable and only includes polarizability effect in a mean-field fashion possibly contributing the method systematic error.

The CASSCF(12,12) level of theory is followed by an energy correction at the multi-configurational second-order perturbation level (CASPT2) to recover the missing dynamical electron correlation. Thus, a 3-roots state-average CASPT2 that uses the three-root stage-average CASSCF(12,12)/6-31 G(d)/AMBER as the zero-order reference wavefunction, is computed (CASTP2(12,12)/6-31 G(d)/AMBER). Ultimately, each of the 10 model replicas correspond to an equilibrated gas-phase and globally uncharged monomer QM/MM model and it is associated with a ΔE_S1–S0_ calculated between S_0_ → S_1_. Finally, the average ΔE_S1–S0_ is reported along with the corresponding standard deviation (see Section Supplementary Note 3).

### Validation, capabilities, and potential applications of *a*-ARM

The *a*-ARM protocol, in its current version, does not represent a predictive tool but rather is designed to produce models useful for investigating the origin of trends in spectroscopic/photochemical properties (e.g., between sequence variability and function) emerging from different sets of experimental data. Furthermore, the models are used assuming that single amino acid replacements will change the protein conformation at the level of the chromophore cavity. Accordingly, after demonstrating that the models reproduce, within the protocol error bar (see below), the trend in observed vertical excitation energy ΔE_S1–S0_ (equivalent to the λ^a^_max_ value), the QM/MM models are employed to study the factors (i.e., steric and electrostatic contributions to ΔE_S1–S0_) determining the ΔE_S1–S0_ change of each mutant with respect to the WT-KR2. This is only possible because the level of protocol automation guarantees, regardless of the user ability or computational facility, reproducible *a*-ARM QM/MM models when the same parameters are employed in Phases I and II of Fig. 4b, c. Indeed, the production of the default *a*-ARM model (see *a*-ARM_default_ approach in Supplementary Note 2) is fully automated and does not require human intervention thus preventing the generation of biased models. Furthermore, the absence of parameters required at the input stage, prevents overfitting. On the other hand, in specific cases, mostly related to the difficulties in predicting the correct ionization state of ionizable groups surrounding the chromophore it is necessary to customize the default model by following a well-defined protocol (see *a*-ARM_customized_ approach in Supplementary Note 2). Such a systematic (non-arbitrary) protocol, documented in refs. ^9,30^. and summarized in Supplementary Note 2, involves three steps which only concern the ionization states of the ionizable residues that, as reported in refs. ^9,30^. and anticipated above, is the most frequent cause of inconsistencies between the computed and observed excitation energies. The customization procedure does not represent an overfitting of the model but a systematic ionization state scan also used by other groups working on rhodopsin simulations (see for instance refs. ^13,32,33^).

The *a*-ARM models described above (see Supplementary Note 10 and Supplementary Fig. 6) have been successfully benchmarked by reproducing the trends in ΔE_S1–S0_ of a set of 44 rhodopsins (25 of wild type and 19 mutants)^9,30,35–37,42^. More specifically, the *a*-ARM_default_ approach proved to be capable of reproducing the observed ΔE_S1−S0_ values for 79% (35/44) of the models, whereas the other 21% were successfully obtained with the *a*-ARM_customized_ approach (i.e., customizing the protonation states pattern). The estimated trend deviation, relative to the probed set using as a reference bovine rhodopsin (Rh), has been reported as 0.7 ± 0.5 kcal mol^−1^ (0.03 ± 0.02 eV) and the mean absolute error (MAE) (see definition in Supplementary Note 6) as 1.0 kcal mol^−1^ (0.04 eV)^9^. Both computed and observed trends in ΔE_S1–S0_ for the benchmark set, that comprises microbial and animal rhodopsins whose structures were obtained from either X-ray crystallography or comparative modeling, is presented in Supplementary Figure 7.

Considering that in this study we are interested in trends in vertical excitation energies, and more specifically red- or blue-shifts of these values, the results are presented and discussed in terms of mutation-induced shifts with respect to WT-KR2 rather than differences between observed and calculated values. On the other hand, the observed ΔE_S1–S0_ displays a remarkable experimental “asymmetry” with 18 over 19 single site variants featuring a red-shifted ΔE_S1–S0_ values compressed in a 1.0 kcal mol^−1^ (0.04 eV or ca. 10 nm) wide range. Such relatively small difference has prevented a robust individual quantitative analysis of the color variation of these mutants. For these reasons the red-shifted mutants have been collected in a single cluster and the analysis is mainly performed by focusing on the origin of the ΔE_S1–S0_ change when going from WT-KR2 to the center of the cluster. Still, the less and most red-shifted mutants falling parallel to the correlation line (P219G and P219H respectively) are individually discussed. The limitations and pitfalls of the protocol are summarized in Supplementary Note 12.

### Automation of the mutant rotamer selection

The QM/MM modeling of the mutants required a second step of experimentally-driven customization dealing with the residue side-chain conformation (i.e., rotamer selection). That procedure consisted in evaluating the model performance for a set of automatically chosen rotamers and select the one that better reproduces the observed ΔE_S1–S0_ value (we assume $\varDelta {{{{{{\rm{E}}}}}}}_{{{{{{\rm{S1}}}}}}-{{{{{\rm{S0}}}}}}}^{{{{{\mathrm{Exp}}}}}}={{{{{\rm{hc}}}}}}/{{{\lambda }}}_{{{{{{\rm{max }}}}}}}^{{{{{{\rm{a}}}}}},{{{{\mathrm{Exp}}}}}}$ and use $\varDelta {{{{{{\rm{E}}}}}}}_{{{{{{\rm{S1}}}}}}-{{{{{\rm{S0}}}}}}}^{{{{{\mathrm{Exp}}}}}}$ and ${{{\lambda }}}_{{{{{{\rm{max }}}}}}}^{{{{{{\rm{a}}}}}},{{{{\mathrm{Exp}}}}}}$ interchangeably in the following). To this aim, an update version of the mutation routine of the input generator (see step 3 in Fig. 4b), that uses Modeller^43^ instead of the default rotamer library SCWRL4^44^, is proposed as part of the present work. A detailed description of the new approach is provided in Supplementary Note 13. Briefly, in order to explore the performance of different rotamers of the mutated side-chain, three *a*-ARM QM/MM models featuring the three highest scored mutated side-chain rotamers selected by Modeller^43^, are produced and their ΔE_S1–S0_ evaluated (see Supplementary Fig. 7). Then, the model that better reproduces the observed ΔE_S1–S0_ is selected (see Supplementary Fig. 8). To perform such selection, we use, as a baseline, the difference between the computed and observed ΔE_S1–S0_ of the WT-KR2, hereafter referred to as ΔΔE^Exp,WT^_S1–S0_. The equivalent quantity calculated for each rotamer (ΔΔE^Exp,rotX^_S1–S0_, with X=1, 2, 3) is then contrasted with the ΔΔE^Exp,WT^_S1–S0_ via the equation rotX = (ΔΔE^Exp,rotX^_S1–S0_ − ΔΔE^Exp,WT^_S1–S0_) (see Supplementary Fig. 9). The rotamer that features the lower rotX value (preferring blue-shifted values) is chosen as the representative *a*-ARM model (see Supplementary Fig. 10). Although this approach relies on experimental information and does not represent a predictive tool, it automates the side-chain conformation selection during the construction of mutant models aimed at the reproduction of experimental trends in properties. Some limitations and pitfalls of the proposed approach are provided in Supplementary Note 13.

### Statistics and reproducibility

As further described in “QM/MM Modeling” section and Supplementary Note 3, each *a*-ARM QM/MM model is composed of n = 10 independent replicas that correspond to an equilibrated gas-phase and globally uncharged monomer, and it is associated with a ΔE_S1–S0_ calculated between S_0_ → S_1_. As shown in Fig. 5c, for each model the average ΔE_S1–S0_ is reported (see green triangles) along with the corresponding standard deviation (see green error bars). Finally, the replica with ΔE_S1–S0_ closest to the average (see red squares) is selected as a representative model for the color tuning analyses. The production of the models, as well as the computation of the average and standard deviation, and the posterior selection of the representative replica is performed automatically by the *a*-ARM protocol illustrated in Fig. 4. Therefore, the reproducibility of the data is guaranteed when selecting the same initial seeds for the n = 10 MD runs. On the other hand, the statistical parameters employed for the different computational analyses, *i.e*., mean absolute error (MAE), mean absolute deviation (MAD), and weighted average $(\bar{x})$ are described in Supplementary Note 6 and the equations are provided. Furthermore, the methodology employed for the calculation of the weighted average is provided in Supplementary Note 6, along with an illustrative example.

### Reporting summary

Further information on research design is available in the Nature Research Reporting Summary linked to this article.

## Supplementary information

Supplementary Information

Supplementary Data 1

Supplementary Data 2

Supplementary Data 3

Supplementary Data 4

Supplementary Data 5

Supplementary Data 6

Supplementary Data 7

Supplementary Data 8

Supplementary Data 9

Supplementary Data 10

Supplementary Data 11

Supplementary Data 12

Supplementary Data 13

Supplementary Data 14

Supplementary Data 15

Supplementary Data 16

Supplementary Data 17

Supplementary Data 18

Supplementary Data 19

Supplementary Data 20

Supplementary Data 21

Reporting Summary

## Data Availability

All experimental data shown in main figures were deposited in Supplementary Data 1. XYZ Coordinates for the optimized S_0_ *a*-ARM QM/MM models are provided as Supplementary Data 2-21 as follows: P219A (Supplementary Data 2.xyz), P219C (Supplementary Data 3.xyz), P219D (Supplementary Data 4.xyz), P219E (Supplementary Data 5.xyz), P219F (Supplementary Data 6.xyz), P219G (Supplementary Data 7.xyz), P219H (Supplementary Data 8.xyz), P219I (Supplementary Data 9.xyz), P219K (Supplementary Data 10.xyz), P219L (Supplementary Data 11.xyz), P219M (Supplementary Data 12.xyz), P219N (Supplementary Data 13.xyz), P219Q (Supplementary Data 14.xyz), P219R (Supplementary Data 15.xyz), P219S (Supplementary Data 16.xyz), P219T (Supplementary Data 17.xyz), P219V (Supplementary Data 18.xyz), P219W (Supplementary Data 19.xyz), P219Y (Supplementary Data 20.xyz), and WT-KR2 (Supplementary Data 21.xyz). Any remaining information can be obtained from the corresponding author upon reasonable request.

## Code availability

Code for the *a*-ARM protocol used in the computational experiments for the QM/MM modeling and color tuning analysis of P219X mutants of KR2 is currently only installed in our cluster facilities but can be distributed via GitLab repository under user request. The manuscript concerning the publication of the computational package that implements all the *a*-ARM protocol/features is work in progress.
