# Supplementary material for: Pro219 is an electrostatic color determinant in the light-driven sodium pump KR2
Source: Commun Biol. 2021 Oct 13;4:1185. doi: 10.1038/s42003-021-02684-z (PMC8514524; doi:10.1038/s42003-021-02684-z)
Supplement: Supplementary file 1 — Supplementary Information [file 42003_2021_2684_MOESM1_ESM.pdf]

Supplementary Information:

Role of Pro219 as an Electrostatic Color Determinant in the Light-driven Sodium Pump KR2: Combined spectroscopic and QM/MM modelling studies

Yuta Nakajima[$1], Laura Pedraza-González[$2], Leonardo Barneschi[2], Keiichi Inoue[3], Massimo Olivucci*[2,4], and Hideki Kandori*[1,5]

[1]Department of Life Science and Applied Chemistry, Nagoya Institute of Technology, Showa-ku, Nagoya 466-8555, Japan
[2]Dipartimento di Biotecnologie, Chimica e Farmacia, Università di Siena, Via A. Moro 2, I-53100 Siena, Italy
[3]The Institute for Solid State Physics, The University of Tokyo, 5-1-5 Kashiwanoha, Kashiwa, Chiba 277-8581, Japan
[4]Department of Chemistry, Bowling Green State University, Bowling Green, Ohio 43403, United States
[5]OptoBioTechnology Research Center, Nagoya Institute of Technology, Showa-ku, Nagoya 466-8555, Japan
[$]Equally contributed.

*Correspondence and requests for materials should be addressed to M.O. (email: massimo.olivucci@unisi.it) or H.K. (email: kandori@nitech.ac.jp)

Supplementary Note 1. Experimental Supplementary data

- **Colors of the variants**

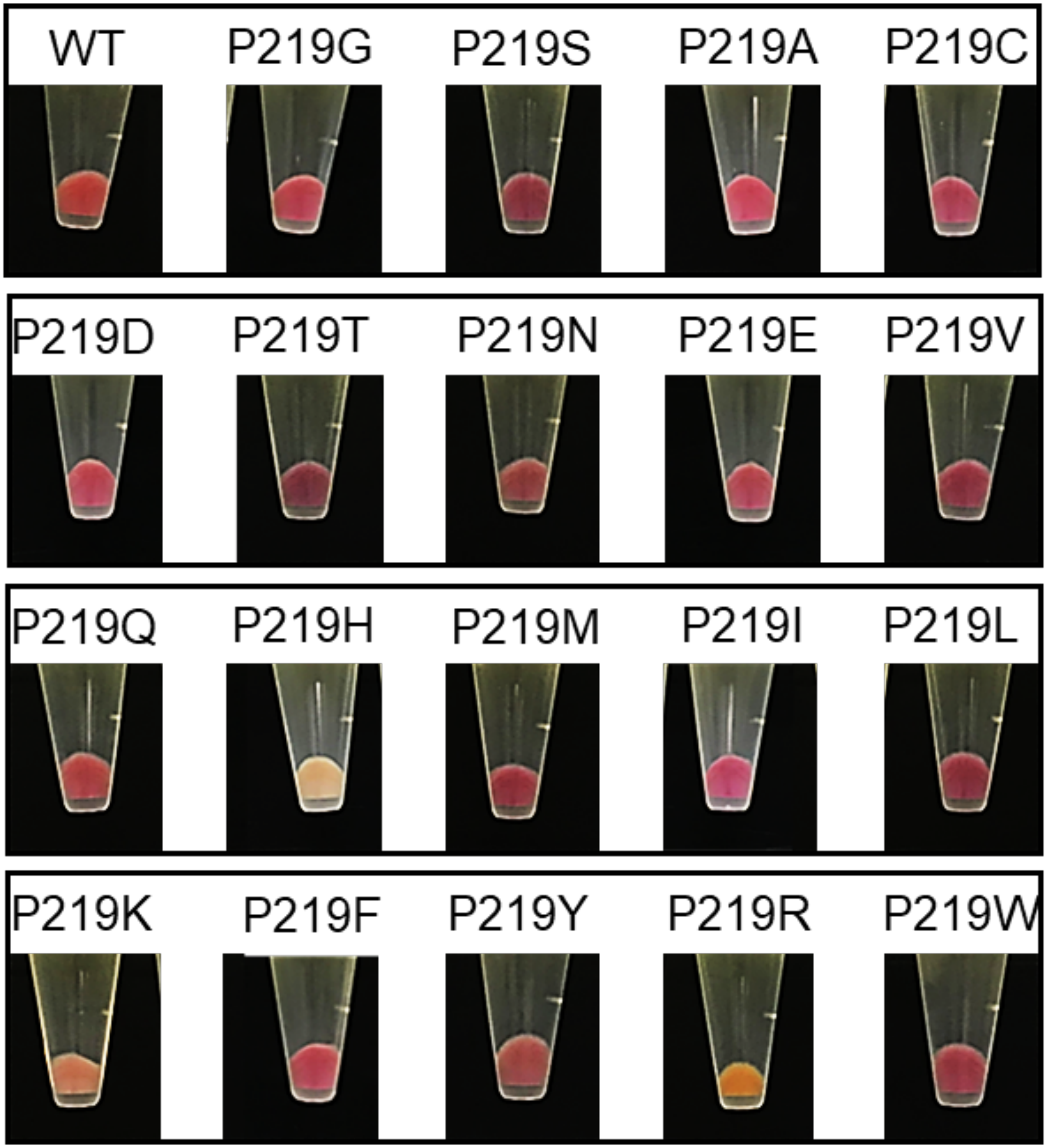

Supplementary Figure 1 Colors of the samples for wild-type KR2 and 19 of its P219X (X = A, C, D, E, F, G, H, I, K, L, M, N, Q, R, S, T, V, W, Y) variants.

- **Expression levels**

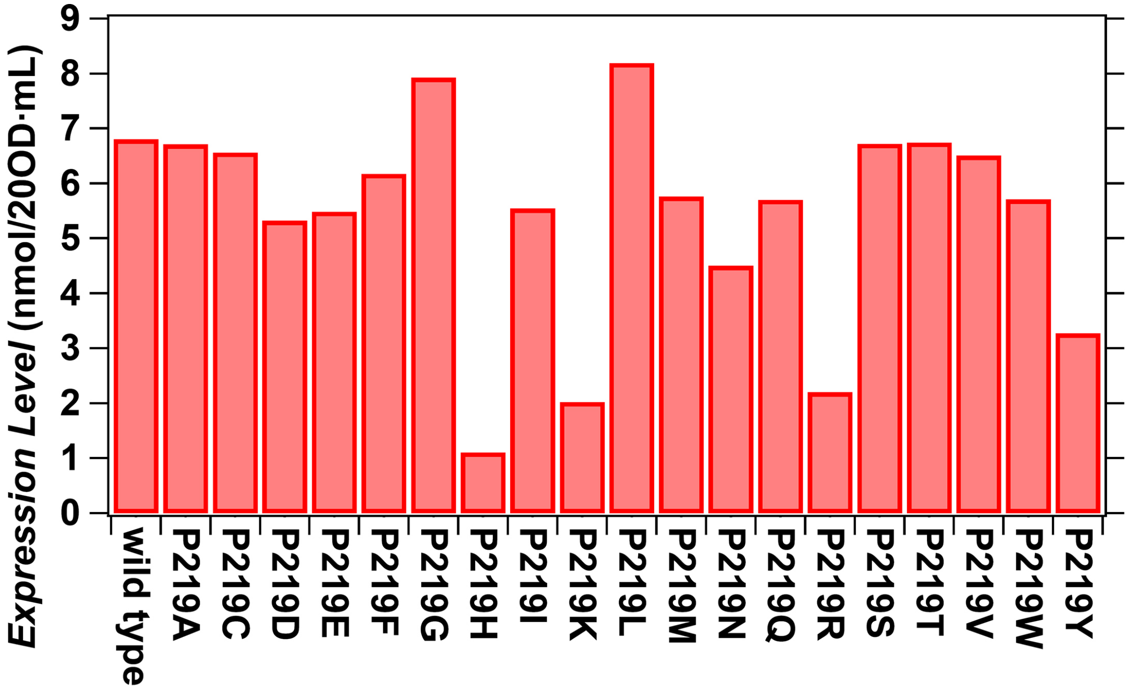

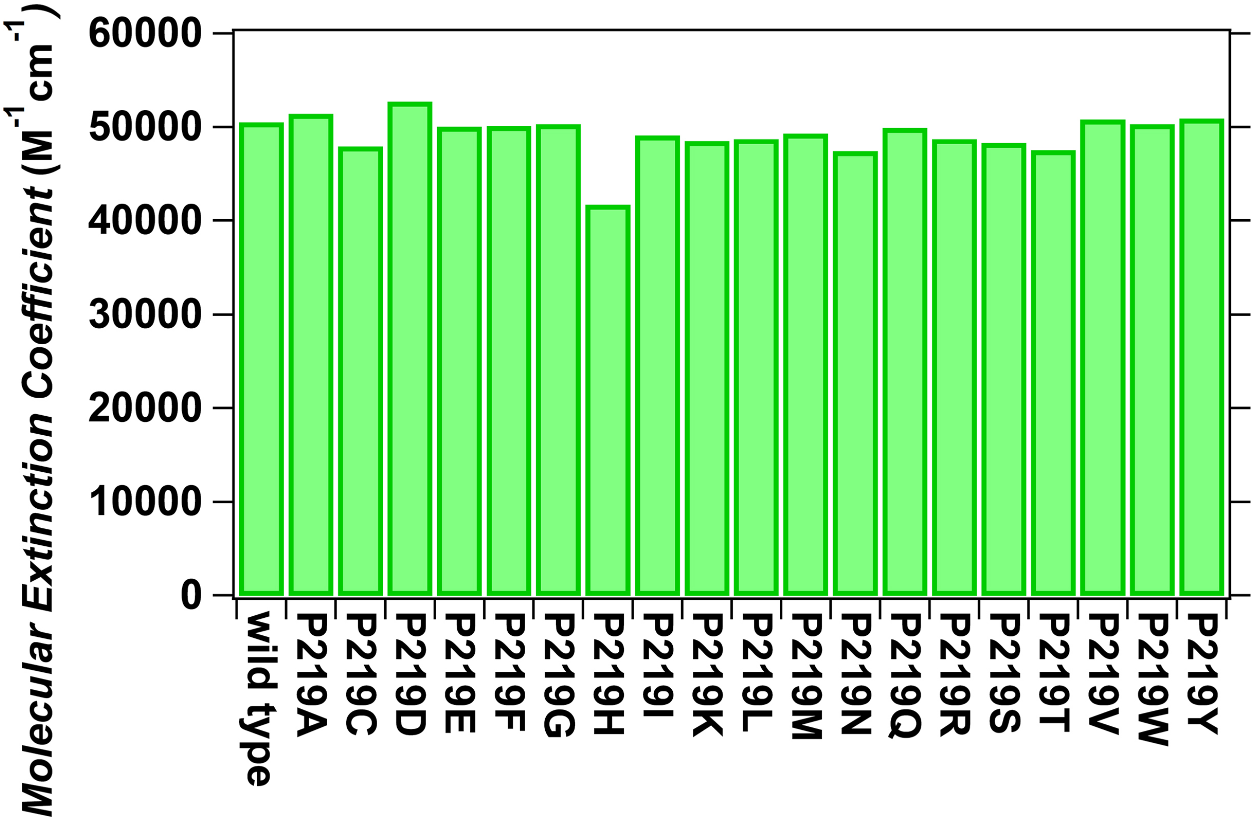

Supplementary Figure 2 Expression levels (a) and molecular extinction coefficients (b) of each protein.

Supplementary Note 2. Phase I: Input File Generator of *a*-ARM

The first phase of *a*-ARM (Figure 4b) allows either the automatic or semi-automatic computer-aided preparation (*i.e.*, via a five-step command-line procedure) of a 3D structure in PDB format (without hydrogens), which contains information on: the monomeric protein structure, *automatically* selected as Chain A but *user-customizable*, including the retinal proton Schiff base (rPSB) chromophore and excluding membrane lipids and non-functional ions (step 1, Section 2.2.1 of Ref. [1]); the mutant(s) *automatically* produced via side-chain replacement using MODELLER [2] (step 3); the protonation states for all the ionizable residues based on an algorithm that analyze pK_a and partial charges using PROPKA [3], *automatically* assigned but *user-customizable* (step 4, Section 2.2.3 of Ref. [1]); the positions of Cl^-/Na^+ external counterions needed to neutralize both IS and OS, based on an energy minimization procedure using PUTION [4], optimized *automatically* and *not user-customizable* (step 5, Section 2.2.4 of Ref. [1]); and an independent file containing the list of amino acid residues forming the cavity hosting the rPSB, determined *automatically* with Fpocket [5] but *user-customizable* (step 2, Section 2.2.2 of Ref. [1]). The resulting PDB structure (PDB^{ARM}) plus cavity file constitute the so-called *a*-ARM input, for the *QM/MM model generator* phase (see below), that according to the parameters selected (via command-line) in steps 1-5 can be considered as *a*-$ARM_{default}$ or *a*-$ARM_{customized}$. While the former refers to a fully automatic input generation, which uses default parameters as suggested by the code (see above), the latter allows the command-line assisted user-customization of some of them.

- Model customization

The ***a*-$ARM_{customized}$** customized approach, that is specifically used in cases where the default choices produce QM/MM models that are not suitable for the reproduction of trends in absorption properties, can be performed by adopting well-defined guided-procedure easily replicable. This protocol, documented in Refs. [1] and [6], involves three phases which only concern the ionization states of the ionizable residues: i) when the pH is > 6 we modify the ionization states by setting the pH to 5.2 in step 4 and ii) we check the protonation state of the main and secondary conterions of the rPSB, and if our analysis give them both ionized we neutralize the secondary conterion and iii) in case the model generated in step ii does not reproduce the experimental absorption maxima, then we exchange the secondary and main counterion ionization states. Supplementary Figure 3 illustrates the customization of the WT-KR2 model, described in the main manuscript, in which steps i) and ii) were employed.

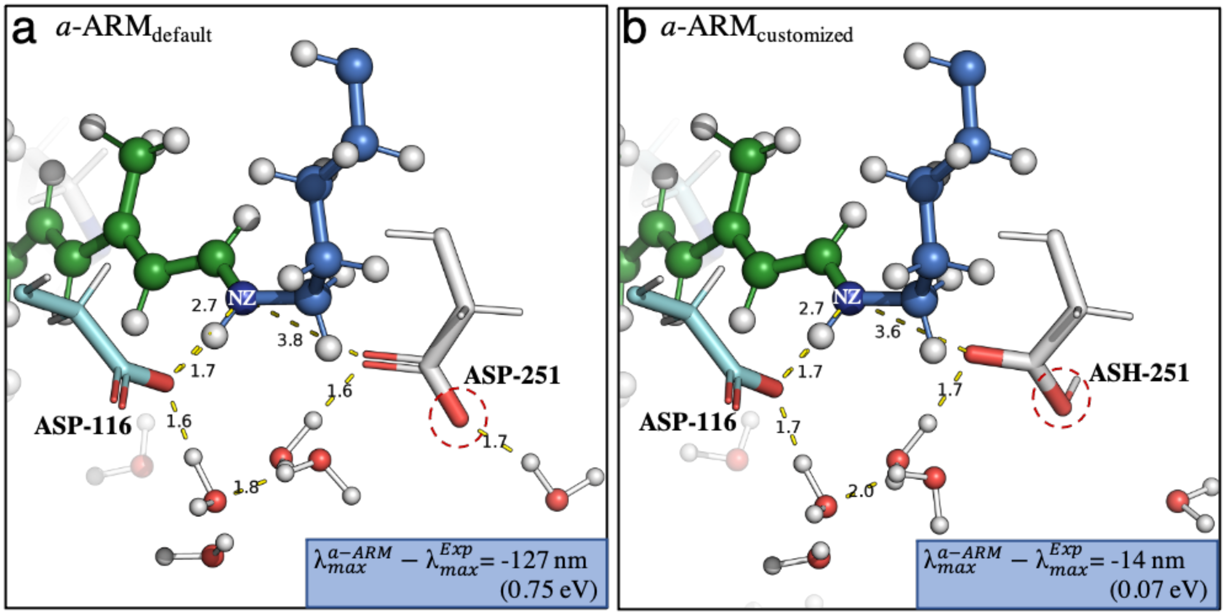

Supplementary Figure 3 Model customization. (a) Default model generated with the *a*-$ARM_{default}$ approach and (b) customized model produced with the *a*-$ARM_{customized}$ approach. The customization was performed by i) predicting the protonation states for ionizable residues at pH 5.2, and ii) neutralizing the secondary counterion of the rPSBAT (ASP-251 → ASH-251). QM/MM-optimized models, with hydrogen bonds represented as dashed lines.

Supplementary Table 1 Features of the customized WT-KR2 QM/MM model. Overview of structural features and both experimental and computational data for customized ARM QM/MM model of wild-type *Krokinobacter eikastus* rhodopsin (KR2).

***Krokinobacter eikastus* rhodopsin (KR2)**			
General Information			
PDB ID:	6REW[1]	Chromophore:	Retinal (RET)
RET Conformation:	all-*trans* (AT)	Lysine Linker:	Lysine 255 (K255)
Proton acceptor:	Aspartic 116 (D116)	Proton Donor:	Aspartic 251 (D251)
Spectroscopic Information			
Absorption:	ΔE_{S1-S0}: 54.5 kcal mol^{-1} (2.36 eV)	λ^{a}_{max}: 525 nm	
pKa Analysis at crystallographic pH 5.2			
Protonated residues:	ASH 251 GLH 160		
Counterion Distribution:	Inner surface: 8 Cl^-	Outer surface: 6 Na^+ (6 Na^+ crystallographic)	
Cavity Residues:	TYR-110, TRP-113, ASP-116, VAL-117, LEU-120, MET-149, ILE-150, GLY-153, PHE-167, LEU-168, GLY-171, ALA-172, SER-174, SER-175, PHE-178, TRP-215, TYR-218, PRO-219, TYR-222, LEU-223, TYR-247, ASP-251, SER-254, LYS-255		
Computational Results			
Absorption^a:	ΔE_{S1-S0}: 56.0 kcal mol^{-1} (2.43 eV)	λ^{a}_{max}: 511 nm	f_{Osc}: 1.15
Error:	+1.5 kcal mol^{-1}		
Standard Deviation (DEV.ST):	0.1 kcal mol^{-1}		

Supplementary Table 2 Calculated pKa computed with PROPKA3.1. pKa computed at pH 5.2 and 7.0 and partial charges obtained in "step 4" of the input file generator of *a*-ARM. The only residue sensitive to pH change is Glu160. In the PROPKA calculations, the retinal chromophore is not included in the PQR file.

		**pH 5.2**		**pH 7.0**	
**KR2 variant**	**Residue**	**pKa^{calc}**	**Charge**	**pKa^{calc}**	**Charge**
WT	Asp116	4.770	-1	4.770	-1
	Asp251	3.120	-1	3.120	-1
	Glu160	6.300	0	6.300	-1
P219R	Asp116	4.770	-1	4.770	-1
	Asp251	3.120	-1	3.120	-1
	Glu160	6.300	0	6.300	-1
P219H	Asp116	4.770	-1	4.770	-1
	Asp251	3.120	-1	3.120	-1
	Glu160	6.300	0	6.300	-1

Supplementary Note 3. Phase II: QM/MM model generator of *a*-ARM

The second phase (Figure 4c) allows the automatic generation of ground-state (S_0) QM/MM models for rhodopsins (Figure 4a) and the subsequent computation of the maximum absorption wavelength (λ^{a}_{max}) via vertical excitation energy ($\Delta E_{S1\text{-}S0}$) calculations. The procedure is described as follows:

- Classical molecular dynamics simulations

a-ARM input is pre-processed using classical Molecular Dynamics (MD) simulations. First, the positions of crystallographic/comparative waters are optimized and the hydrogens for waters and polar residues are added by using DOWSER [7]. Then, the hydrogens for the rest of the protein and chromophore are added and their positions are optimized by a Molecular Mechanics (MM) energy minimization using GROMACS [8]. A second MM energy minimization is performed, this time on the side-chains (backbone atoms are fixed at the crystallographic/comparative positions) of the residues belonging to the chromophore cavity sub-system. The resulting structure is employed as an input to generate *N*=10 independent simulated annealing/MD relaxations at 298 K, each starting with a different randomly chosen seed to warrant independent initial conditions that allow to explore the possible relative conformational phase space of the cavity residue side-chains and chromophore. In the ARM MD approach, that uses GROMACS [8] and AMBER [9] force field, all side-chains of the Lys-QM and chromophore cavity (including cavity waters) subsystems are relaxed, while the backbone is fixed at the crystallographic/comparative structure. The Lys-QM subsystem is described by using a MM parametrization and partial charges computed as AMBER-like Restrained Electrostatic Potential (RESP) charges, which are specifically parametrized for each employed isomer of the chromophore (e.g., 11-*cis*, all-*trans* and 13-*cis* rPSB). [4] Such parameters are reported in the Supporting Information of Ref. [1]. Moreover, the default heating, equilibration, and production times for the MD (selected via benchmark calculations in Ref. [4]) are 50, 150, and 800 fs, respectively, for a total length of 1 ns. For each of the *N*=10 replicas, the *frame closest to the average structure* of the 1 ns simulation is selected as the starting geometry (i.e., guess structure) for constructing the corresponding QM/MM model.

- QM/MM calculations

Each of the 10 replicas (*i.e.*, frame extracted from the N=10 independent MDs) is processed by a particular QM/MM approach implemented into the [Open]Molcas/TINKER [10, 11]] interface [12], where the electrostatic embedding scheme used to describe the interaction between the QM and MM parts of the Lys-QM sub-system (see Figure 4a), involves an unconventional treatment called Electrostatic Potential Fitted (ESPF) [13]. In the ESPF method, the QM part of the chromophore directly interacts with the MM electrostatic potential through one-electron operators whose expectation values represent the QM charge distribution of the chromophore. Notice that

mutual polarization effects between the QM and MM sub-systems are not considered. Although this issue can, in principle, be solved by employing a polarizable embedding method, we have not adopted/benchmarked such technologies in this first version of our specialized QM/MM models since they are still under development in the QM/MM area (see for instance Ref. [14]). In addition, the QM/MM frontier is treated within a link atom approach whose position is restrained according to the Morokuma scheme, and it is placed across the covalently bonded lysine Cε-Cδ bond (where Cε is a QM atom). The charges of the covalently linked lysine are modified by setting the Cδ charge to zero to avoid hyperpolarization and redistribute the residual fractional charge on the most electronegative atoms of the lysine, thus ensuring a +1 integer charge of the Lys-QM layer. All the 63 Lys-QM atoms (i.e., 62 atoms + linker atom) are free to relax during the QM/MM calculation. By employing such an approach, the procedure to obtain an ARM QM/MM model consisting of N=10 replicas, can be described as follows. First, to complete the pre-processing step, a geometry optimization at the Hartree-Fock (HF) level is performed (HF/3-21G/AMBER). Then, another geometry optimization is carried out this time modeling the QM sub-system with the multi-configurational complete active space self-consistent field (CASSCF) at the 2-roots single-state, (CASSCF(12,12)/6-31G(d)/AMBER level). This follows an energy correction at the multi-configurational second-order perturbation theory (CASPT2) to recover the missing dynamical electron correlation associated with the CASSCF description. Thus, a 3-roots state-average CASPT2 that uses the three-root stage-average CASSCF(12,12)/6-31G(d)/AMBER as the zero-order reference wavefunction, is computed (CASTP2(12,12)/6-31G(d)/AMBER). Ultimately, each model replica corresponds to an equilibrated gas-phase and globally uncharged monomer QM/MM model and it is associated with a calculated between $S_0 \rightarrow S_1$ $\Delta E_{S1\text{-}S0}$. The final *a*-ARM result is the average of the 10 ΔE_{S1-S0} values. A detailed explanation of the *a*-ARM protocol workflow is provided in Refs. [1] and [6].

Supplementary Note 4. Computed vs experimental vertical excitation energies

Supplementary Table 3 *a*-ARM QM/MM calculations for the wild-type KR2 (WT-KR2) rhodopsin and 19 of its mutants (P219*X*, with *X*= A, C, D, E, F, G, H, I, K, L, M, N, [illegible] R, S, T, V, W, Y). Total Energies calculated at the CASSCF/AMBER//CASPT2/6-21G(d) level. First vertical excitation energy (ΔE_{S1-S0}), maximum absorption wavelength (λ^{a}_{max}), transitio[illegible] oscillator strength (f_{Osc}), and difference between calculated and experimental data ($\Delta\Delta E^{Exp,a\text{-}ARM}_{S1\text{-}S0}$). Standard deviation for the *N*=10 replicas (σ_N) is presented as subindex

	**Experimental**		***a*-ARM (*N*=10)**						***a*-ARM (*N*=1) replica with $\Delta E^{a\text{-}ARM}_{S1\text{-}S0}$ closest to the average**					
Variant	$\Delta E^{Exp}_{S1\text{-}S0}$ **(kcal mol⁻¹)**	$\lambda^{a,Exp}_{max}$ **(nm)**	**CASPT2 S_0 (a.u.)**	**CASPT2 S_1 (a.u.)**	$\Delta E^{a\text{-}ARM}_{S1\text{-}S0}$ **(kcal mol⁻¹)**	$\lambda^{a,a\text{-}ARM}_{max}$ **(nm)**	**f_{Osc}**	$\Delta\Delta E^{Exp,a\text{-}ARM}_{S1\text{-}S0}$ **(kcal mol⁻¹)**	**CASPT2 S_0 (a.u.)**	**CASPT2 S_1 (a.u.)**	$\Delta E^{a\text{-}ARM}_{S1\text{-}S0}$ **(kcal mol⁻¹)**	$\lambda^{a,a\text{-}ARM}_{max}$ **(nm)**	**f_{Osc}**	$\Delta\Delta E^{Exp,a\text{-}ARM}_{S1\text{-}S0}$ **(kcal mol⁻¹)**
WT	54.5	525	-871.958317	-871.869104	$56.0_{0.1}$	511	1.15	1.5	-871.958527	-871.869347	56.0	511	1.15	1.5
P219A	53.4	536	-871.950645	-871.861691	$55.8_{0.1}$	512	1.16	2.5	-871.951699	-871.862795	55.8	513	1.16	2.4
P219C	53.2	537	-871.941631	-871.853410	$55.4_{0.3}$	516	1.20	2.1	-871.941957	-871.853675	55.4	516	1.20	2.2
P219D	53.1	539	-872.016177	-871.928564	$55.0_{0.2}$	520	1.21	1.9	-872.016302	-871.928624	55.0	520	1.22	2.0
P219E	52.8	541	-872.036157	-871.948780	$54.8_{0.2}$	521	1.22	2.0	-872.037094	-871.949834	54.8	522	1.24	1.9
P219F	53.1	538	-871.958854	-871.871312	$54.9_{0.3}$	520	1.23	1.8	-871.959496	-871.871956	54.9	520	1.22	1.8
P219G	53.4	535	-871.991864	-871.903387	$55.5_{0.1}$	515	1.17	2.1	-871.948714	-871.859831	55.8	513	1.17	2.4
P219H	52.5	545	-871.958008	-871.870980	$54.6_{0.1}$	524	1.22	2.1	-871.958554	-871.871679	54.5	524	1.22	2.0
P219I	52.8	541	-871.966959	-871.878610	$55.4_{0.2}$	516	1.17	2.6	-871.967532	-871.879282	55.4	516	1.22	2.6
P219K	53.3	536	-871.986167	-871.898719	$54.9_{0.5}$	521	1.25	1.6	-871.986337	-871.898941	54.8	521	1.22	1.5
P219L	52.8	541	-871.993038	-871.905303	$55.1_{0.2}$	519	1.22	2.2	-871.993437	-871.905624	55.1	519	1.20	2.3
P219M	53.1	538	-871.967225	-871.879950	$54.8_{0.2}$	522	1.21	1.7	-871.967229	-871.879951	54.8	522	1.21	1.7
P219N	52.8	541	-872.075645	-871.988734	$54.5_{0.4}$	524	1.20	1.7	-872.075929	-871.989032	54.5	524	1.21	1.7
P219Q	53.4	535	-872.050851	-871.963231	$55.0_{0.3}$	520	1.24	1.5	-872.051077	-871.963597	54.9	521	1.23	1.5
P219R	55.5	515	-872.239434	-872.148460	$57.1_{0.2}$	501	0.98	1.6	-872.240147	-872.149282	57.0	501	0.96	1.5
P219S	53.2	538	-871.951792	-871.864686	$54.7_{0.1}$	523	1.24	1.5	-871.952480	-871.865352	54.7	523	1.24	1.5
P219T	52.8	541	-871.999472	-871.911216	$55.4_{0.4}$	516	1.20	2.6	-871.999588	-871.911214	55.5	516	1.19	2.6
P219V	53.0	540	-871.989047	-871.901705	$54.8_{0.5}$	522	1.24	1.8	-871.988240	-871.901176	54.6	523	1.31	1.6
P219W	53.0	539	-871.950915	-871.863147	$55.1_{0.1}$	519	1.22	2.1	-871.951024	-871.863228	55.1	519	1.22	2.1
P219Y	53.3	537	-871.996567	-871.908542	$55.2_{0.2}$	518	1.19	2.0	-871.997169	-871.909270	55.2	518	1.19	1.9
							MAE	1.9					MAE	1.9
							AD_{max}	2.6					AD_{max}	2.6
							MAE	0.3					MAE	0.3

Supplementary Note 5. Comparison of WT-KR2 models built with original ARM

Although the conclusions derived from the study of Inoue et. al.[33] can be qualitatively compared with the results presented in this work, notice that they cannot be quantitatively compared since i) the QM/MM models were constructed from a different X-ray structure (i.e., 3X3C), using the original ARM [15] that featured ii) manual input file generation (i.e., handmade and not reproducible counterion placement, different chromophore cavity, etc.) and iii) a different methodological approach for the generation of the mutant side-chain. Remarkably, we have verified that the side-chain conformation of the P219T mutant selected automatically in *a*-ARM (rotamer 3) is equivalent to the side-chain used by Inoue et al. in the original ARM (see Supplementary Figure 4).

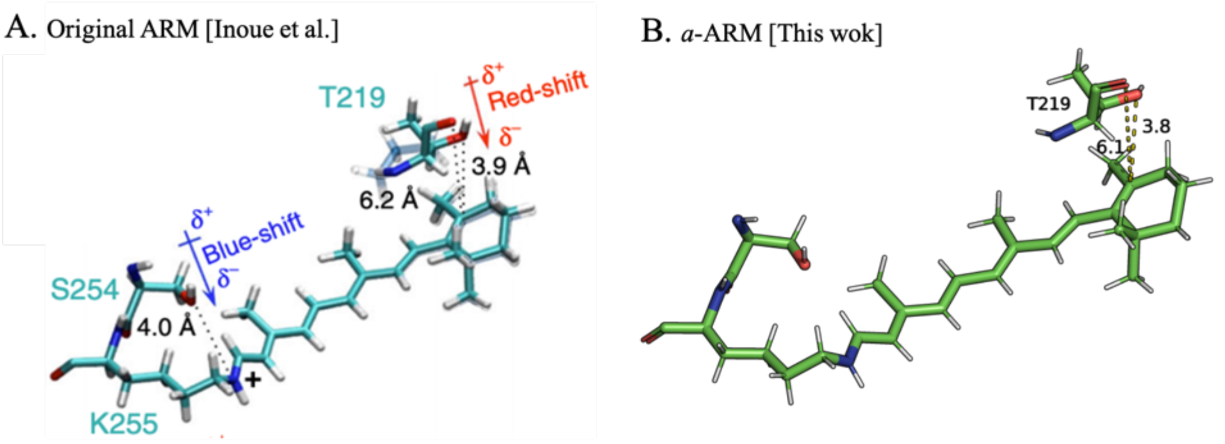

Supplementary Figure 4 Comparison between side-chain conformation of P219T mutant reported by Inoue et al. (a) and used in this work (b). The same rotamer is selected.

Then, consistently with what reported by Inoue et al., we have analyzed only the "steric" and "total electrostatic" components of the vertical excitation energy. Such results, reported in Supplementary Table 4 demonstrates that although the computed magnitudes are different, the signs are the same, suggesting that both versions of the protocol produce consistent results.

Supplementary Table 4 Vertical excitation energies of the retinal chromophore incorporated in the protein and in vacuum, using both the original and the *a*-ARM versions of ARM.

KR2 variant	ARM version	ΔE^a_{S1-S0} (protein) (kcal mol^{-1})	ΔE^a_{S1-S0} (vacuum) (kcal mol^{-1})	$\Delta\Delta E^a_{S1-S0}$ (protein-vacuum) (kcal mol^{-1})
WT-KR2	Original	55.2	43.1	+12.1
	a-ARM	56.0	45.1	+10.9
P219G	Original	54.3 (-0.9)	43.8 (+0.7)	+10.5 (-1.6)
	a-ARM	55.8 (-0.2)	45.1 (0.0)	+10.7 (-0.2)
P219T [R3]	Original	53.5 (-1.7)	44.5 (+1.3)	+9.0 (-3.1)

Supplementary Note 6. Statistics

- Mean absolute error

It is a measure of errors (e_i) between paired data that can be, *e.g.*, predicted (y_i) versus observed (x_i).

$$MAE = \frac{\sum_{i=1}^{n} |y_i - x_i|}{n} = \frac{\sum_{i=1}^{n} |e_i|}{n}$$

As has been established in Ref. [1], the MAE is computed inside the *a*-ARM framework to estimate the ability of the protocol to predict photophysical properties and then be able to compare the results in trends for heterogeneous sets of rhodopsin variants. For instance, in the main text we have compared the results in vertical excitation energy obtained for the benchmark set in Ref. [1], with the results obtained in this work for the KR2 set.

- Mean absolute deviation

As has been established in Ref. [1], the MAD is computed inside the *a*-ARM framework as a measure of dispersion, that represents how much the absolute errors between computed and experimental values in photophysical properties in the data set (e_i, see definition above) are likely to differ from their MAE. The absolute value is used to avoid deviations with opposite signs cancelling each other. The MAE is calculated by using the following formula:

$$MAD = \frac{1}{n} \sum_{i=1}^{n} |e_i - MAE|,$$

Where n is the number of rhodopsin variants. See Section 6.2.1.

- Weighted average

The weighted average ($\bar{x}$) takes into account the varying degrees of importance of the numbers in a data set. It is equal to the sum of the product of the weight (w_i) times the data number (x_i) divided by the sum of the weights:

$$\bar{x} = \frac{\sum_{i=1}^{n} w_i \cdot x_i}{\sum_{i=1}^{n} w_i} = \frac{w_1 x_1 + w_2 x_2 + \ldots + w_n x_n}{w_1 + w_2 + \ldots + w_n}$$

Supplementary Note 7. Parallelism between computed and experimental values: weighted average

In order to evaluate the parallelism between calculated and experimental data, we have computed the trend deviation defined in the previous section as

$$||\text{Trend Dev.}|| = |\Delta_{max,X}^{WT,Exp} - \Delta_{max,X}^{WT,a\text{-}ARM}|,$$

where $\Delta_{max,X}^{WT,Exp}$ is the difference between experimental λ_{max} of each of the P219X mutants with respect to the experimental value of the WT, while $\Delta_{max,X}^{WT,a\text{-}ARM}$ is the difference between *a*-ARM computed λ_{max} of each of the P219X mutants with respect to the *a*-ARM computed value of the WT.

According with both $\Delta_{max,X}^{WT,Exp}$ and $\Delta_{max,X}^{WT,a\text{-}ARM}$ values, reported in Supplementary Table 5 and Figure 5d, we have classified the 19 P219X KR2 variants into two clusters, namely "blue-shifting" and "red-shifting" clusters. The former is composed of the only blue-shifted variant, P219R, while the latter is composed of the other 18 variants. The main purpose of such cluster definition is to threat the data of variants of a same cluster in a weighted average fashion, instead of discussing individual values. We felt that such a treatment is necessary since, as explained in the main manuscript, the <2.0 kcal mol^{-1} observed $\Delta_{max,X}^{WT,Exp}$ variations among members of the red-shifting cluster includes, in most cases, too small (we set a threshold at ≤1 kcal mol^{-1}) for the $\Delta_{max,X}^{WT,Exp}$ trend to be safely reproduced by a *a*-ARM QM/MM model. In order to locate the cluster center, we selected a weighted average to give more importance to the most frequent deviations seen in our small data sample. Accordingly, the weighted average of the red-shifting cluster, for both computed and experimental data, was computed following the next procedure (notice that the results are not substantially different from the results obtained with a standard average. See Supplementary Table 5.)

- Computation of the individual weights for the red-shifting cluster:

In order to compute the individual weights for each of the members of the red-shifting cluster, a histogram for both $\Delta_{max,X}^{WT,Exp}$ and $\Delta_{max,X}^{WT,a\text{-}ARM}$ was generated, using the data (in kcal mol^{-1}) presented in Supplementary Table 5. Then, the entire range of values was divided into a series of intervals, using a bin width of 0.2 kcal mol^{-1}, and the weights were calculated as the frequency of the data in the corresponding interval.

Compute the weighted average value for the red-shifted cluster. By using the data of the histograms of Supplementary Figure 5, we assigned a weight for both the $\Delta_{max,X}^{WT,Exp}$ and $\Delta_{max,X}^{WT,a\text{-}ARM}$ values for each of the members of the red-shifting cluster, as reported in Supplementary Table 5. Finally, we computed the weighted average of the red-shifting cluster, for both experimental and computed data, by using the equation shown in Supplementary Note 6, as -1.38 and -0.97 kcal mol^{-1}, respectively. Since the definition of such weighted average values mean the average difference in vertical excitation energy between the center of the red-shifting cluster and the wild type (WT-KR2), we add such values to the experimental and computed data of the WT-KR2. In this way, we obtained the coordinate [538.3,519.8] that we plot as black point in Figure 5b.

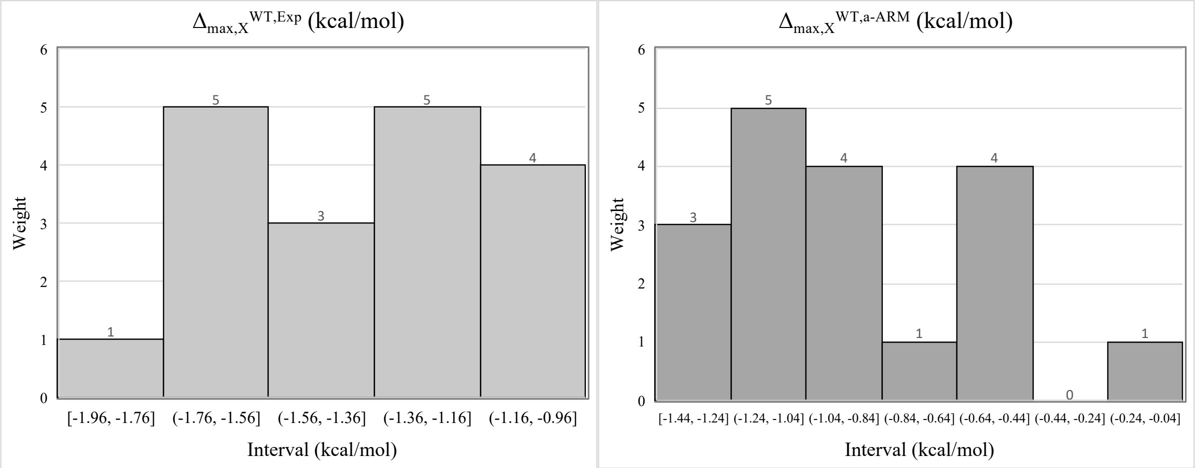

Supplementary Figure 5 Histograms of frequency for $\Delta_{max,X}^{WT,Exp}$ and $\Delta_{max,X}^{WT,a\text{-}ARM}$.

- Trend deviation

Supplementary Table 5 Trend deviation factor (||Trend Dev.||) for the *a*-ARM models. Values are expressed as mean absolute error (MAE) and mean absolute deviation (MAD) of the x=19 mutants of KR2.

	Experimental						*a*-ARM (*N*=10)						‖Trend Dev.‖[(c)]		
	λ_{max}^{Exp}			$\Delta_{max,X}^{WT,Exp\ (a)}$			$\lambda_{max}^{a\text{-}ARM}$			$\Delta_{max,X}^{WT,a\text{-}ARM\ (b)}$					
**P219X mutations**	**(nm)**	**(kcal mol^{-1})**	**(eV)**	**(nm)**	**(kcal mol^{-1})**	**(eV)**	**(nm)^c**	**(kcal mol^{-1})**	**(eV)**	**(nm)**	**(kcal mol^{-1})**	**(eV)**	**(nm)**	**(kcal mol^{-1})**	**(eV)**
WT	525	54.5	2.36	0	0.0	0.00	510.7	56.0	2.43	0	0.0	0.00	0	0.0	0.00
P219A	536	53.4	2.31	11	-1.1	-0.05	512.2	55.8	2.42	1	-0.2	-0.01	1	0.9	0.04
P219C	537	53.2	2.31	12	-1.2	-0.05	516.5	55.4	2.40	6	-0.6	-0.03	6	0.6	0.03
P219D	539	53.1	2.30	14	-1.4	-0.06	520.1	55.0	2.38	9	-1.0	-0.04	9	0.4	0.02
P219E	541	52.8	2.29	16	-1.6	-0.07	521.5	54.8	2.38	11	-1.2	-0.05	11	0.5	0.02
P219F	538	53.1	2.30	13	-1.3	-0.06	520.5	54.9	2.38	10	-1.0	-0.05	10	0.3	0.01
P219G	535	53.4	2.32	10	-1.0	-0.05	515.0	55.5	2.41	4	-0.5	-0.02	4	0.6	0.02
P219H	545	52.5	2.28	20	-2.0	-0.08	523.6	54.6	2.37	13	-1.4	-0.06	13	0.6	0.03
P219I	541	52.8	2.29	16	-1.6	-0.07	515.7	55.4	2.40	5	-0.5	-0.02	5	1.1	0.05
P219K	536	53.3	2.31	11	-1.2	-0.05	521.0	54.9	2.38	10	-1.1	-0.05	10	0.0	0.00
P219L	541	52.8	2.29	16	-1.6	-0.07	519.3	55.1	2.39	9	-0.9	-0.04	9	0.7	0.03
P219M	538	53.1	2.30	13	-1.3	-0.06	522.1	54.8	2.37	11	-1.2	-0.05	11	0.1	0.01
P219N	541	52.8	2.29	16	-1.6	-0.07	524.3	54.5	2.36	14	-1.4	-0.06	14	0.2	0.01
P219Q	535	53.4	2.32	10	-1.0	-0.04	520.0	55.0	2.38	9	-1.0	-0.04	9	0.0	0.00
P219R	515	55.5	2.41	-10	1.1	0.05	500.8	57.1	2.48	-10	1.1	0.05	-10	0.0	0.00
P219S	538	53.2	2.30	13	-1.3	-0.06	523.1	54.7	2.37	12	-1.3	-0.06	12	0.0	0.00
P219T	541	52.8	2.29	16	-1.6	-0.07	516.3	55.4	2.40	6	-0.6	-0.03	6	1.0	0.05
P219V	540	53.0	2.30	15	-1.5	-0.06	521.7	54.8	2.38	11	-1.2	-0.05	11	0.3	0.01
P219W	539	53.0	2.30	14	-1.4	-0.06	519.1	55.1	2.39	8	-0.9	-0.04	8	0.5	0.02
P219Y	537	53.3	2.31	12	-1.2	-0.05	517.6	55.2	2.40	7	-0.7	-0.03	7	0.4	0.02
MAE of ‖Trend Dev.‖														0.4	0.02
MAD of ‖Trend Dev.‖														0.3	0.01

[(a)] Difference between experimental λ_{max} of each of the P219X mutants with respect to the experimental value of the WT ($\Delta_{max,X}^{WT,Exp}$); [(b)] Difference between calculated *a*-ARM λ_{max} of each of the P219X mutants with respect to the calculated *a*-ARM value of the WT ($\Delta_{max,X}^{WT,a\text{-}ARM}$); [c)] ‖Trend Dev.‖ = $|\Delta_{max,X}^{WT,Exp} - \Delta_{max,X}^{WT,a\text{-}ARM}|$

Supplementary Table 6 Weighted average values for both experimental and computed vertical excitation energy, as well as for the different components, for the red-shifted cluster. The standard average is also reported. Values are presented in kcal mol^{-1}.

Red-shifted P219X mutations	$\Delta\Delta E^{Exp}_{S1-S0}$			$\Delta\Delta E^{a-ARM}_{S1-S0}$			$\Delta\Delta E^{STR}_{S1-S0}$			$\Delta\Delta E^{ELE(t)}_{S1-S0}$			$\Delta\Delta E^{ELE(i)}_{S1-S0}$			$\Delta\Delta E^{ELE(d)}_{S1-S0}$		
	x_i	ω_i	$x_i*\omega i$	x_i	ω_i	$x_i*\omega i$	x_i	ω_i	$x_i*\omega i$	x_i	ω_i	$x_i*\omega i$	x_i	ω_i	$x_i*\omega i$	x_i	ω_i	$x_i*\omega i$
P219A	-1.11	4	-4.43	-0.2	1	-0.16	0.1	9	0.49	-0.2	2	-0.45	0.1	2	0.23	-0.3	9	-3.10
P219C [R1]	-1.24	5	-6.18	-0.6	4	-2.49	0.1	9	0.52	-0.6	2	-1.24	-0.1	5	-0.68	-0.5	1	-0.49
P219D [R1]	-1.40	3	-4.21	-1.0	4	-4.02	0.2	9	1.45	-1.1	3	-3.31	-0.7	4	-2.96	-0.4	9	-3.26
P219E [R3]	-1.63	5	-8.15	-1.2	5	-5.76	0.3	2	0.64	-1.5	4	-6.10	-0.7	4	-2.98	-0.8	4	-3.12
P219F [R3]	-1.33	5	-6.63	-1.0	5	-5.24	0.2	9	1.43	-1.2	5	-5.94	-0.5	2	-0.91	-0.7	4	-2.93
P219G	-1.04	4	-4.15	-0.5	4	-1.85	0.0	7	0.17	-0.2	2	-0.42	0.3	2	0.51	-0.5	9	-4.21
P219H(E)[R3]	-1.96	1	-1.96	-1.4	3	-4.11	-0.1	7	-0.55	-1.4	4	-5.47	0.1	1	0.06	-1.4	1	-1.43
P219I [R1]	-1.64	5	-8.20	-0.5	4	-2.17	0.4	2	0.89	-1.0	3	-3.08	-0.7	1	-0.68	-0.3	9	-3.09
P219K [R3]	-1.16	4	-4.63	-1.1	5	-5.54	0.1	9	1.33	-1.3	5	-6.34	-0.8	4	-3.18	-0.5	9	-4.26
P219L [R1]	-1.64	5	-8.20	-0.9	4	-3.71	-0.2	7	-1.07	-0.7	2	-1.41	-0.3	5	-1.51	-0.4	9	-3.63
P219M [R2]	-1.35	5	-6.73	-1.2	5	-6.08	0.2	9	1.74	-1.4	4	-5.55	-0.3	5	-1.34	-1.1	1	-1.12
P219N [R2]	-1.61	5	-8.05	-1.4	3	-4.33	0.1	9	0.88	-1.5	4	-6.12	-1.1	2	-2.22	-0.4	9	-3.79
P219Q [R1]	-1.02	4	-4.07	-1.0	4	-4.00	0.2	9	1.37	-1.2	5	-6.09	0.7	1	0.66	-1.9	1	-1.88
P219S [R3]	-1.31	5	-6.53	-1.3	3	-3.97	0.0	7	-0.05	-1.3	5	-6.40	-1.0	2	-1.96	-0.3	9	-2.72
P219T [R1]	-1.64	5	-8.20	-0.6	4	-2.40	-0.1	7	-0.79	-0.4	1	-0.39	-0.4	2	-0.72	0.0	1	-0.03
P219V [R1]	-1.46	3	-4.39	-1.2	5	-5.87	-0.1	7	-0.47	-1.3	5	-6.30	-0.8	4	-3.40	-0.4	9	-3.70
P219W [R1]	-1.43	3	-4.30	-0.9	4	-3.63	0.0	7	0.21	-0.9	1	-0.90	-0.1	5	-0.55	-0.8	4	-3.16
P219Y [R2]	-1.19	5	-5.94	-0.7	1	-0.75	0.2	9	1.54	-1.0	3	-2.93	-0.1	5	-0.63	-0.8	4	-3.39
Weighted average	**-1.38**			**-0.97**			**0.07**			**-1.14**			**-0.40**			**-0.48**		
Average	**-1.40**			**-0.93**			**0.09**			**-1.01**			**-0.36**			**-0.65**		

Supplementary Note 8. Steric and Electrostatic contributions

Supplementary Table 7 Steric ($\Delta\Delta E^{STR}_{S1\text{-}S0}$) and electrostatic ($\Delta\Delta E^{ELE}_{S1\text{-}S0}$) contributions of the interaction of the retinal with the protein environment for the P219*X* (*X*= A, C, D, E, F, G, H, I, K, L, M, N, Q, R, S, T, V, W, Y). The decomposition of the total ($\Delta\Delta E^{ELE(t)}_{S1\text{-}S0}$) electrostatic effects on its indirect ($\Delta\Delta E^{ELE(i)}_{S1\text{-}S0}$) and direct ($\Delta\Delta E^{ELE(d)}_{S1\text{-}S0}$) components is also shown. All the values are presented in kcal/mol.

KR2 Variant	$\Delta\Delta E^{TOT}_{S1\text{-}S0}$ (kcal/mol)	$\Delta\Delta E^{STR}_{S1\text{-}S0}$ (kcal/mol)	$\Delta\Delta E^{ELE(t)}_{S1\text{-}S0}$ (kcal/mol)	$\Delta\Delta E^{OFF}_{S1\text{-}S0}$ (kcal/mol)	$\Delta\Delta E^{ELE(i)}_{S1\text{-}S0}$ (kcal/mol)	$\Delta\Delta E^{ELE(d)}_{S1\text{-}S0}$ (kcal/mol)
WT	0.0	0.0	0.0	0.0	0.0	0.0
P219A	-0.2	0.1	-0.2	0.2	0.1	-0.3
P219C	-0.6	0.1	-0.6	-0.1	-0.1	-0.5
P219D	-0.9	0.2	-1.1	-0.6	-0.7	-0.4
P219E	-1.2	0.3	-1.5	-0.4	-0.7	-0.8
P219F	-1.0	0.2	-1.2	-0.3	-0.5	-0.7
P219G	-0.2	0.0	-0.2	0.3	0.3	-0.5
P219H	-1.4	-0.1	-1.4	0.0	0.1	-1.4
P219I	-0.6	0.4	-1.0	-0.2	-0.7	-0.3
P219K	-1.1	0.1	-1.3	-0.6	-0.8	-0.5
P219L	-0.9	-0.2	-0.7	-0.5	-0.3	-0.4
P219M	-1.2	0.2	-1.4	-0.1	-0.3	-1.1
P219N	-1.4	0.1	-1.5	-1.0	-1.1	-0.4
P219Q	-1.1	0.2	-1.2	0.8	0.7	-1.9
P219R	1.1	-0.8	1.8	-6.7	-6.0	7.8
P219S	-1.3	0.0	-1.3	-1.0	-1.0	-0.3
P219T	-0.5	-0.1	-0.4	-0.5	-0.4	0.0
P219V	-1.3	-0.1	-1.3	-0.9	-0.8	-0.4
P219W	-0.9	0.0	-0.9	-0.1	-0.1	-0.8
P219Y	-0.8	0.2	-1.0	0.0	-0.1	-0.8

- $$\Delta\Delta E^{TOT}_{S1\text{-}S0} = \Delta E^{MUT}_{S1\text{-}S0} - \Delta E^{WT}_{S1\text{-}S0}$$
- $$\Delta\Delta E^{STR}_{S1\text{-}S0} = \Delta E^{RET,MUT}_{S1\text{-}S0} - \Delta E^{RET,WT}_{S1\text{-}S0}$$
- $$\Delta\Delta E^{ELE(t)}_{S1\text{-}S0} = \Delta\Delta E^{TOT}_{S1\text{-}S0} - \Delta\Delta E^{STR}_{S1\text{-}S0}$$
- $$\Delta\Delta E^{OFF}_{S1\text{-}S0} = \Delta E^{OFF,MUT}_{S1\text{-}S0} - \Delta E^{OFF,WT}_{S1\text{-}S0}$$
- $$\Delta\Delta E^{ELE(i)}_{S1\text{-}S0} = \Delta\Delta E^{OFF}_{S1\text{-}S0} - \Delta\Delta E^{STR}_{S1\text{-}S0}$$
- $$\Delta\Delta E^{ELE(d)}_{S1\text{-}S0} = \Delta\Delta E^{ELE(t)}_{S1\text{-}S0} - \Delta\Delta E^{ELE(i)}_{S1\text{-}S0}$$

Supplementary Note 9. Sequence of KR2 and primers for mutagenesis

Amino acid sequence of KR2
MTQELGNANFENFIGATEGFSEIAYQFTSHILTLGYAVMLAGLLYFILTIKNVDKKFQMS
NILSAVVMVSAFLLLYAQAQNWTSSFTFNEEVGRYFLDPSGDLFNNGYRYLNWLIDVP
MLLFQILFVVSLTTSKFSSVRNQFWFSGAMMIITGYIGQFYEVSNLTAFLVWGAISSAFFF
HILWVMKKVINEGKEGISPAGQKILSNIWILFLISWTLYPGAYLMPYLTGVDGFLYSEDG
VMARQLVYTIADVSSKVIYGVLLGNLAITLSKNKELVEANSLE

DNA sequence of synthetic KR2 gene
ATGACCCAGGAATTAGGTAATGCCAACTTTGAGAACTTCATTGGTGCGACTGAAGG
GTTCTCGGAAATCGCGTATCAGTTTACCTCGCATATTCTGACCTTAGGCTATGCGGTG
ATGCTGGCTGGCCTTCTGTACTTTATCCTTACGATTAAAAATGTCGACAAGAAATTC
CAGATGAGCAACATTCTGAGTGCAGTGGTTATGGTAAGCGCTTTTCTGCTCTTGTAT
GCACAAGCGCAAAATTGGACGTCATCTTTCACCTTCAATGAAGAAGTGGGGCGTTAC
TTTCTGGATCCTAGTGGTGACCTGTTCAACAACGGCTATCGCTACCTGAATTGGCTG
ATTGACGTTCCGATGCTTTTGTTTCAGATCCTGTTTGTGGTTAGTCTGACCACCTCCA
AATTTAGCTCTGTCCGCAATCAGTTTTGGTTCTCAGGTGCCATGATGATCATTACAGG
CTATATCGGACAGTTTTACGAAGTGTCCAACCTGACTGCGTTTCTGGTCTGGGGAGC
CATTAGCAGTGCGTTCTTCTTTCACATTCTCTGGGTTATGAAGAAAGTGATCAATGA
GGGCAAAGAGGGCATTTCACCGGCTGGTCAGAAAATCCTGAGCAACATCTGGATTC
TGTTTCTGATCTCTTGGACGTTGTACCCAGGTGCGTATTTAATGCCGTATTTAACAGG
CGTAGATGGGTTCCTGTACAGCGAAGATGGCGTTATGGCACGTCAACTGGTGTATAC
GATTGCAGATGTGTCGTCGAAAGTCATTTATGGCGTTCTCCTTGGTAATCTGGCCATT
ACCTTGTCCAAGAACAAAGAGCTCGTAGAAGCCAACAGCCTCGAG

Supplementary Table 8 Sequences of primers used for mutagenesis of KR2 P219. The sense and anti-sense primers used for the mutagenesis of KR2 P219.

Mutant	Primer type	Primer sequence
KR2 P219A	Sense	CTCTTGGACGTTGTACGCGGGTGCGTATTTAATG
	Anti-sense	CATTAAATACGCACCCGCGTACAACGTCCAAGAG
KR2 P219C	Sense	CTCTTGGACGTTGTACTGCGGTGCGTATTTAATG
	Anti-sense	CATTAAATACGCACCGCAGTACAACGTCCAAGAG
KR2 P219D	Sense	CTCTTGGACGTTGTACGATGGTGCGTATTTAATG
	Anti-sense	CATTAAATACGCACCATCGTACAACGTCCAAGAG
KR2 P219E	Sense	CTCTTGGACGTTGTACGAAGGTGCGTATTTAATG
	Anti-sense	CATTAAATACGCACCTTCGTACAACGTCCAAGAG
KR2 P219F	Sense	CTCTTGGACGTTGTACTTTGGTGCGTATTTAATG

	Anti-sense	CATTAAATACGCACCAAAGTACAACGTCCAAGAG
KR2 P219G	Sense	CTCTTGGACGTTGTACGGCGGTGCGTATTTAATG
	Anti-sense	CATTAAATACGCACCGCCGTACAACGTCCAAGAG
KR2 P219H	Sense	CTCTTGGACGTTGTACCATGGTGCGTATTTAATG
	Anti-sense	CATTAAATACGCACCATGGTACAACGTCCAAGAG
KR2 P219I	Sense	CTCTTGGACGTTGTACATTGGTGCGTATTTAATG
	Anti-sense	CATTAAATACGCACCAATGTACAACGTCCAAGAG
KR2 P219K	Sense	CTCTTGGACGTTGTACAAAGGTGCGTATTTAATG
	Anti-sense	CATTAAATACGCACCTTTGTACAACGTCCAAGAG
KR2 P219L	Sense	CTCTTGGACGTTGTACCTGGGTGCGTATTTAATG
	Anti-sense	CATTAAATACGCACCCAGGTACAACGTCCAAGAG
KR2 P219M	Sense	CTCTTGGACGTTGTACATGGGTGCGTATTTAATG
	Anti-sense	CATTAAATACGCACCCATGTACAACGTCCAAGAG
KR2 P219N	Sense	CTCTTGGACGTTGTACAACGGTGCGTATTTAATG
	Anti-sense	CATTAAATACGCACCGTTGTACAACGTCCAAGAG
KR2 P219Q	Sense	CTCTTGGACGTTGTACCAGGGTGCGTATTTAATG
	Anti-sense	CATTAAATACGCACCCTGGTACAACGTCCAAGAG
KR2 P219R	Sense	CTCTTGGACGTTGTACCGTGGTGCGTATTTAATG
	Anti-sense	CATTAAATACGCACCACGGTACAACGTCCAAGAG
KR2 P219S	Sense	CTCTTGGACGTTGTACAGCGGTGCGTATTTAATG
	Anti-sense	CATTAAATACGCACCGCTGTACAACGTCCAAGAG
KR2 P219T	Sense	CTCTTGGACGTTGTACACCGGTGCGTATTTAATG
	Anti-sense	CATTAAATACGCACCGGTGTACAACGTCCAAGAG
KR2 P219V	Sense	CTCTTGGACGTTGTACGTGGGTGCGTATTTAATG
	Anti-sense	CATTAAATACGCACCCACGTACAACGTCCAAGAG
KR2 P219W	Sense	CTCTTGGACGTTGTACTGGGGTGCGTATTTAATG
	Anti-sense	CATTAAATACGCACCCCAGTACAACGTCCAAGAG
KR2 P219Y	Sense	CTCTTGGACGTTGTACTATGGTGCGTATTTAATG
	Anti-sense	CATTAAATACGCACCATAGTACAACGTCCAAGAG

Supplementary Note 10. Structural comparison between P219X mutants and WT-KR2

Supplementary Figure 6 3D structures for the WT-KR2 and its P219*X* (*X*= A, C, D, E, F, G, H, I, K, L, M, N, Q, R, S, T, V, W, Y) variants. The chromophore and the covalently linked Lys are presented as green and blue balls, respectively, whereas each X mutant is presented as orange sticks. Purple shadow illustrates the reference WT structure.

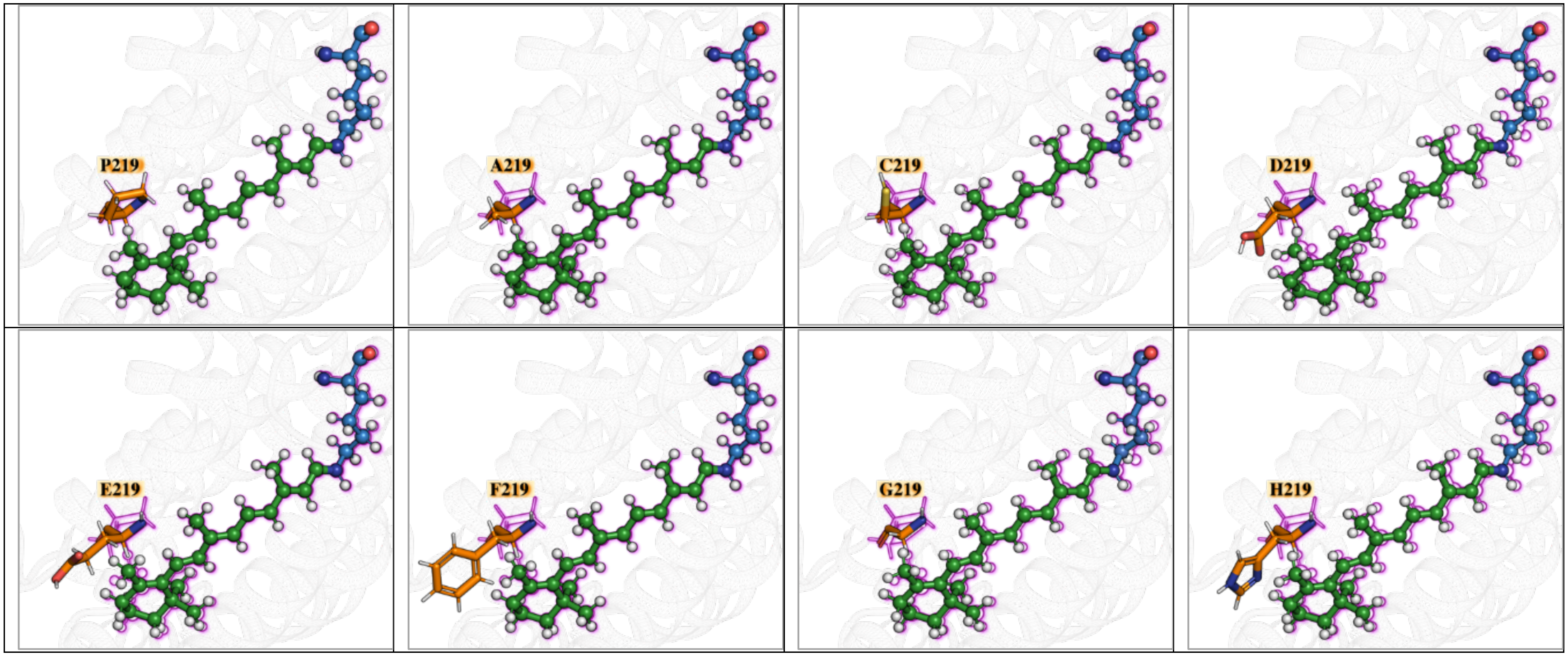

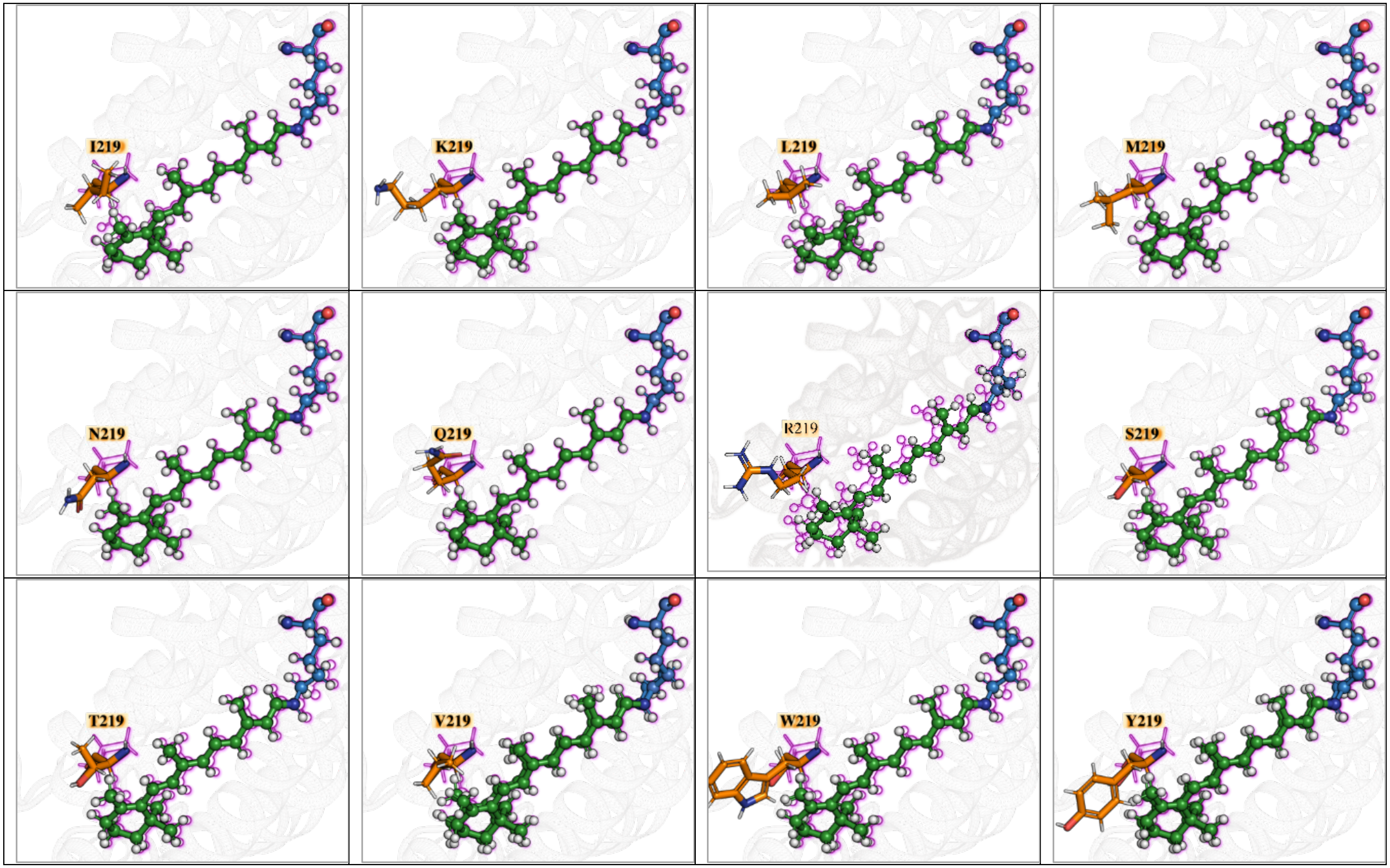
I219
K219
L219
M219
N219
Q219
R219
S219
T219
V219
W219
Y219

Supplementary Note 11. Bond Length Alternation (BLA) for P219X mutants and WT-KR2

Supplementary Table 9 Bond length alternation for WT-KR2 and the P219X mutants. Values are reported in Å.

Variant	C5=C6	C6-C7	C7=C8	C8-C9	C9=C10	C10-C11	C11=C12	C12-C13	C13=C14	C14-C15	C15=N	BLA	BLA^{WT} - BLA^{MUT}
WT	1.364	1.483	1.355	1.472	1.363	1.452	1.358	1.458	1.368	1.432	1.294	0.10923	0.000E+00
P219A	1.364	1.483	1.355	1.472	1.363	1.452	1.357	1.457	1.367	1.431	1.293	0.10898	**2.500E-04**
P219C	1.364	1.483	1.355	1.472	1.363	1.452	1.358	1.457	1.368	1.431	1.294	0.10855	**6.800E-04**
P219D	1.364	1.484	1.355	1.472	1.363	1.452	1.358	1.457	1.369	1.431	1.294	0.10853	**7.000E-04**
P219E	1.364	1.483	1.355	1.472	1.363	1.452	1.358	1.457	1.368	1.431	1.294	0.10827	**9.600E-04**
P219F	1.364	1.482	1.355	1.471	1.363	1.451	1.357	1.456	1.368	1.430	1.294	0.10825	**9.800E-04**
P219G	1.364	1.483	1.355	1.471	1.363	1.452	1.358	1.457	1.368	1.431	1.293	0.10843	**8.000E-04**
P219H	1.361	1.480	1.355	1.470	1.362	1.451	1.357	1.456	1.366	1.430	1.293	0.10840	**8.300E-04**
P219I	1.363	1.481	1.354	1.470	1.361	1.449	1.356	1.455	1.366	1.429	1.293	0.10768	**1.550E-03**
P219K	1.364	1.483	1.355	1.472	1.363	1.451	1.358	1.457	1.368	1.431	1.294	0.10846	**7.700E-04**
P219L	1.364	1.480	1.354	1.470	1.362	1.449	1.357	1.454	1.367	1.428	1.293	0.10734	**1.890E-03**
P219M	1.363	1.482	1.355	1.472	1.362	1.451	1.358	1.456	1.368	1.430	1.294	0.10844	**7.900E-04**
P219N	1.363	1.483	1.356	1.472	1.363	1.452	1.358	1.457	1.368	1.431	1.294	0.10851	**7.200E-04**
P219Q	1.363	1.482	1.355	1.471	1.363	1.451	1.358	1.457	1.368	1.431	1.294	0.10824	**9.900E-04**
P219R	1.361	1.485	1.356	1.473	1.362	1.453	1.358	1.457	1.369	1.428	1.293	0.10936	**-1.300E-04**
P219S	1.364	1.482	1.355	1.471	1.363	1.451	1.358	1.457	1.368	1.431	1.294	0.10784	**1.390E-03**
P219T	1.364	1.483	1.355	1.471	1.363	1.452	1.358	1.457	1.368	1.431	1.294	0.10843	**8.000E-04**
P219V	1.363	1.482	1.355	1.472	1.363	1.451	1.358	1.455	1.370	1.430	1.294	0.10749	**1.740E-03**
P219W	1.365	1.483	1.356	1.471	1.364	1.452	1.359	1.457	1.369	1.431	1.294	0.10808	**1.150E-03**
P219Y	1.364	1.483	1.355	1.471	1.363	1.451	1.357	1.457	1.367	1.430	1.293	0.10847	**7.600E-04**

Supplementary Note 12. Limitations and pitfalls of *a*-ARM

In spite of the encouraging outcome of the photochemical studies based on *a*-ARM, additional work is necessary to generate a tool that can be systematically applied to larger arrays of rhodopsins. In this section, we describe the current methodological limitations of *a*-ARM in terms of both input file generation and QM/MM model building.

Regarding the input generation, three main issues have to be tackled:

- *Assignment of the protonation states*: There are two aspects which limit the confidence in the automation of the ionizable state assignment described above. The first is that, due to the fact that the information provided by PROPKA [3] is approximated (*i.e.,* the retinal chromophore is not included in the PQR file), the computed pK_a^{Calc} value may, in certain cases, be not sufficiently realistic. The second aspect regards the assignment of the correct tautomer of histidine. This amino acid has +1 charge when both the - nitrogen and -nitrogen of the imidazole ring are protonated (HIP), while it is neutral when either the δ-nitrogen (HID) or the ε-nitrogen (HIE) are deprotonated. *a*-ARM uses as default the HID tautomer for the automatic assignment or allow the user to choose between the three tautomers for a not automated selection. Therefore, when possible, the user should collect the available experimental data and/or inspect the chemical environment of the ionizable residues including the histidines and propose the appropriate tautomer. [1, 6] Alternatively, it is necessary to systematically examine all sensible choices which may not always be possible.

- *Automatic construction of comparative models:* since rhodopsin structural data are rarely available, it would be important to investigate the possibility of building, automatically, the corresponding comparative models. With such an additional tool one could achieve a protocol capable of producing QM/MM models starting directly from the constantly growing repositories of rhodopsin amino acid sequences. This target is currently pursued in our lab.

- *Automatic prediction of side-chain conformation for mutants:* In order to achieve a successful technology for systematically predicting mutant structures, a level of accuracy of the *a*-ARM models superior to the one currently available is needed. To deal with that, in this work we attempt at the improvement of the mutations routine by replacing SCWRL4 (i.e., a backbone-dependent rotamer library) with MODELLER a software based on comparative modeling.

Accordingly, their simplified definition makes the ARM models more exposed to potential pitfalls with respect to more complex QM/MM models. Such possible pitfalls can be summarized as: 1) lack of a proper description of the protein environment (membrane + explicit solvent), 2) rigid protein backbone and non-cavity side-chains, 3) approximated protonation states for ionizable residues, 4) missing description of any mutual polarization effects between the QM and MM sub-systems, that can be accounted for by polarizable embedding using a polarizable force field. Since polarizable force fields are technologies still under development in the QM/MM area (see for instance Ref. [14]), we have not adopted/benchmarked them in this first version of our specialized QM/MM models.

Considering points 1-4 above, the different properties computed by ARM are expected to be affected by a systematic error. The current research of our research group is aimed, also, to overcome these points, while maintaining reasonable computational costs, or estimating the errors due to them. Nevertheless, according to the philosophy of the ARM protocol, the main focus of ARM is the ability to reproduce observed trends in vertical excitation energies, as specified in section "Validation, capabilities, and potential applications of *a*-ARM" in the manuscript main text and illustrated in Supplementary Figure 7.

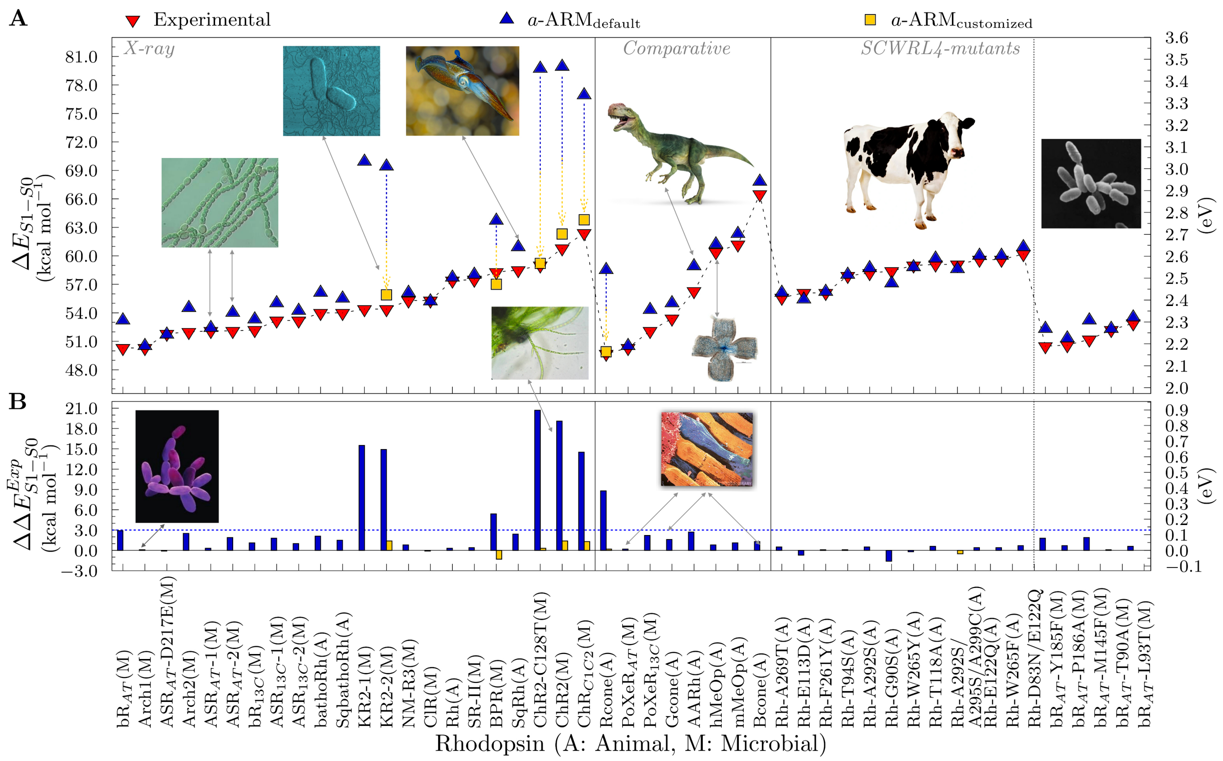

Supplementary Figure 7 ***a*****-ARM protocol validation.** (A) Computed excitation energies $\Delta E_{S1\text{-}S0}$ in both kcal mol^{-1} (left axis) and eV (right axis) for a set of 44 rhodopsin variants. The employed protein structures were obtained from X-ray crystallography (left panel) or through comparative (homology) modeling (center panel). Two sets of variants for bovine rhodopsin (Rh) and bacteriorhodopsin (bR) are also reported (right panel). All computed data were obtained using the *a*-ARM$_{default}$ approach (blue up-turned triangles), and specific models were refined with the *a*-ARM$_{customized}$ (gold squares) approach. Experimental data, as energy difference corresponding to the wavelength of the absorption maxima, are also reported (red down-turned triangles). (B) Differences between computed and experimental excitation energies $\Delta\Delta E_{Exp}^{S1\text{-}S0}$ in both kcal mol^{-1} (left axis) and eV (right axis). The trend deviation of the set, obtained after customization, is 0.7 ± 0.5 kcal mol^{-1} (0.03 ± 0.02 eV) and the mean absolute error (MAE) is 1.0 kcal mol^{-1} (0.04 eV). Reproduced with permissions from **[16]**. Copyright 2020 Wiley Online Library.

Supplementary Note 13. Modification of the mutation routine (step 3) of *a*-ARM

It is well-known that the success of *in silico* modeling of point mutations in proteins, relies on the selection of a robust methodology for the prediction of the side-chain conformation of the replaced amino acid. [17, 18, 19, 20, 21, 22, 23, 24, 25, 26] [27, 28, 29] Both *original* [4] and updated [1, 12, 6] versions of the ARM protocol employ the software SCWRL4 [30] to predict the side-chain conformation of the mutated residues. Such a prediction is based on backbone-dependent rotamer libraries from public databases of experimentally-resolved protein structures. This approach has demonstrated to be effective for the production of single, double and triple point mutants in different rhodopsins that are phylogenetically diverse. More specifically, previous studies carried out by some of the authors were focused on modeling mutants for bovine rhodopsin (Rh), [4] [1] *Anabaena Sensory* rhodopsin (ASR), [4] [31] bacteriorhodopsin (bR) [32] and KR2 rhodopsins. [33] The current research work is, however, the first attempt to use the *a*-ARM rhodopsin model building protocol to systematically mutate a single residue with each of the remaining 19 essential amino acids. More specifically, we performed single point mutations of the residue P219, in the KR2 rhodopsin, that is located near the β-ionone ring of the rPSBAT (see Figure 5a), by replacing the side-chain of the proline (P) with an *in silico* modeled side-chain of each of the other 19 essential amino acids (*i.e.,* P219*X*, with *X*= A, C, D, E, F, G, H, I, K, L, M, N, Q, R, S, T, V, W, Y) via SCWRL4 modelling. In spite of the encouraging results reported for some of the authors in Ref. [33] for the case of P219A, P219G and P219T, we found that the replacement of P219 for larger side-chains performed by SCWRL4 may generate mutated side-chain structures sterically clashing with either the rPSBAT or neighboring amino acids.

In order to overcome the above drawbacks and, thus, achieve a successful technology in terms of predicting mutant spectral properties, it is needed a level of accuracy of the corresponding *a*-ARM models which is superior to the one currently available. Considering the different tools available for side-chain predictions (see for instance Ref. [17]) and evaluating their advantages and pitfalls in terms of i) performance and ii) accessibility as command-line tool, in this work we have modified the routine for mutations in the *a*-ARM rhodopsin model building protocol (see Section 2.2.5. in Ref. [1]) by substituting the SCWRL4 software with Modeller. This alternative approach allows the production of mutants with side-chains suitable for the

prediction of absorption wavelengths in either an automatic or a computer-aided semi-automatic fashion. The general workflow of the proposed subroutine, that constitutes the Step 3 of the *Input file generator* phase of *a*-ARM (see Figure 4b), is illustrated in Supplementary Figure 8. As observed, at the input level of the protocol, each point mutation is generated with a customized version of the `mutate_model.py` routine implemented by Modeller, where the conformation of the modeled side-chain is optimized by conjugate gradient and refined using a short Molecular Dynamics (MD^{mod}). To start the procedure the user should provide a file with extension "`.seqmut`", that contains the list of residues to be mutated. Then, the structure (non-hydrogen atoms protein representation) of the wild-type is used as an input to execute the mutant generator subroutine with the customized setup shown in Supplementary Figure 8. Briefly, as reported in Ref. [2], the `mutate_model.py` script has been designed to model point mutations via side-chain replacement in a fixed environment, assuming that single mutations do not generally determine deep conformational changes of the protein backbone. Accordingly, and also consistently with the structurally "conservative" approach of the *a*-ARM protocol where our models are designed to retain information from the X-ray crystallographic or comparative structures, our methodology replaces only the side-chains of the mutated residues keeping the backbone atoms at fixed positions. To this aim, the optimization of the mutated side-chains is obtained using a combined approach which alternates conjugate gradient minimizations and short MD^{mod} simulations with simulated annealing (for further details see Ref. [2]). As described by Feyfant et al. [34], this intends to minimize a scoring function including homology-derived restraints, force field energy terms (CHARMM22), and a statistical potential for non-bonded interactions. Notice that the MD^{mod} used in this step differs from the MD employed in the QM/MM model generator phase that is described in Supplementary Note 3.

In the above procedure, the script `mutate_model.py` uses a default initial condition or "seed" (variable rand_seed= -49837) for the MD^{mod} simulation. Therefore, since Modeller is deterministic, if such seed value is not modified the MD^{mod} run will always produce the same side-chain conformation when a certain template is used as input. In order to sample more extensively the conformational space of a mutated residue and evaluate its effect on the vertical excitation energy (ΔE^{a}_{S1-S0}), our customized setup produces multiple rotamers of the same mutated side-chain by providing the script with different initial seeds (*i.e.*, initial velocities) for the MD^{mod} run. Thus, our customized approach uses 30 different seeds to potentially generate 30 representative side-chain conformations of a single mutant. Considering that

probabilistically different initial conditions (seeds) may yield the same side-chain conformation, a strategy to discard the duplicates is required. To this aim, the subroutine compares recursively every rotamer with one another and establishes a Root Mean Square Deviation (RMSD) threshold of 0.025 Å, below which we decide that two conformers are identical and one of the two needs to be discarded. Although not particularly efficient from a computational standpoint, given the low number of conformations to evaluate, this procedure allows for the quick selection of a set of non-redundant rotamers for a single mutant. Subsequently, the remaining structures are evaluated by using the scoring function Discrete Optimized Protein Energy (DOPE) [35], implemented by Modeller and ranked from lowest to highest DOPE. The DOPE score is a statistical potential developed by Shen et al. [35] which can be used for external assessment of model accuracy (i.e., it is not involved in the building routine).

The ARM input (see Supplementary Note 2) for the three highest scored mutated side-chain rotamers is completed by phase I of the *a*-ARM protocol, and their ARM QM/MM models are produced using phase II. The corresponding computed ΔE^{a}_{S1-S0} is then used to evaluate the performance of different rotamers of the mutated side-chain, in terms of reproduction of the experimental trend in line with the WT. Subsequently, the model that better reproduces the observed **trend in** ΔE^{Exp}_{S1-S0} is selected. To perform such selection, the difference between the computed ΔE^{a-ARM}_{S1-S0} and observed ΔE^{Exp}_{S1-S0} of the WT, hereafter referred to as $\Delta^{Exp}_{WT} E^{a}_{S1-S0}$, is used as a baseline. The equivalent quantity calculated for each rotamer ($\Delta^{Exp}_{rotX} E^{a}_{S1-S0}$, with X=1,2,3) is then contrasted with the $\Delta^{Exp}_{WT} E^{a}_{S1-S0}$ via the equation:

$$\text{rotX}= \left(\Delta^{\text{Exp}}_{\text{rotX}} E^{a}_{S1\text{-}S0} - \Delta^{\text{Exp}}_{\text{WT}} E^{a}_{S1\text{-}S0}\right) \quad (1)$$

The rotamer that features the lowest rotX value (preferring, blue-shifted values) is chosen as the representative ARM QM/MM model. Although this approach relies on experimental information and does not represent a predictive tool, it automates the side-chain conformation selection during the construction of mutant QM/MM models.

Supplementary Figure 9 illustrates the procedure for selecting the rotamer from the three evaluated models. Furthermore, Supplementary Figure 10 shows the performance of all the possible models generated for the P219X set, while Supplementary Figure 11 reports the 20 models selected for the analyses on color tuning presented in the manuscript.

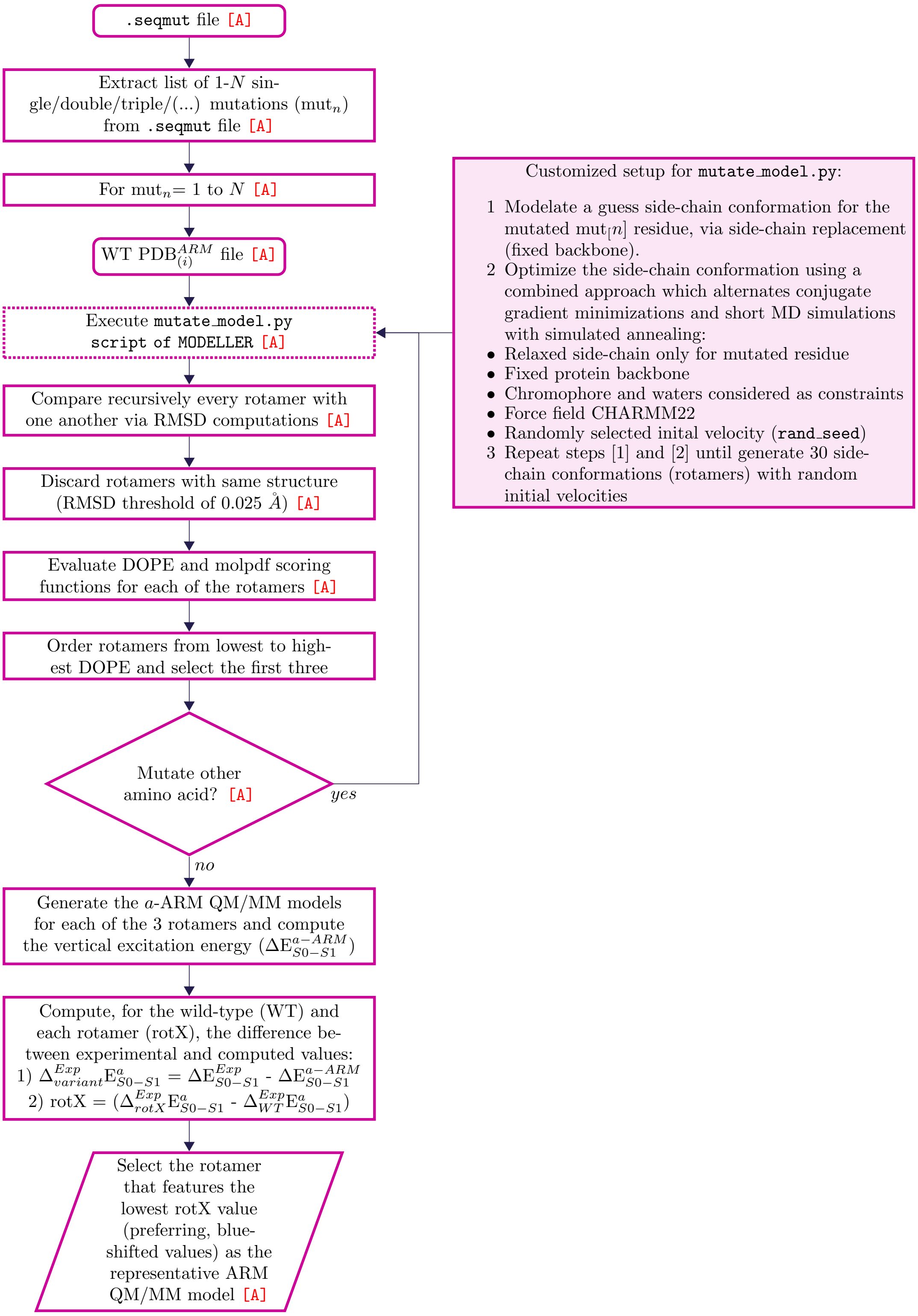

Supplementary Figure 8. General workflow of the side-chain generator. Modified routine for the mutant's generator of a-ARM, using Modeller.

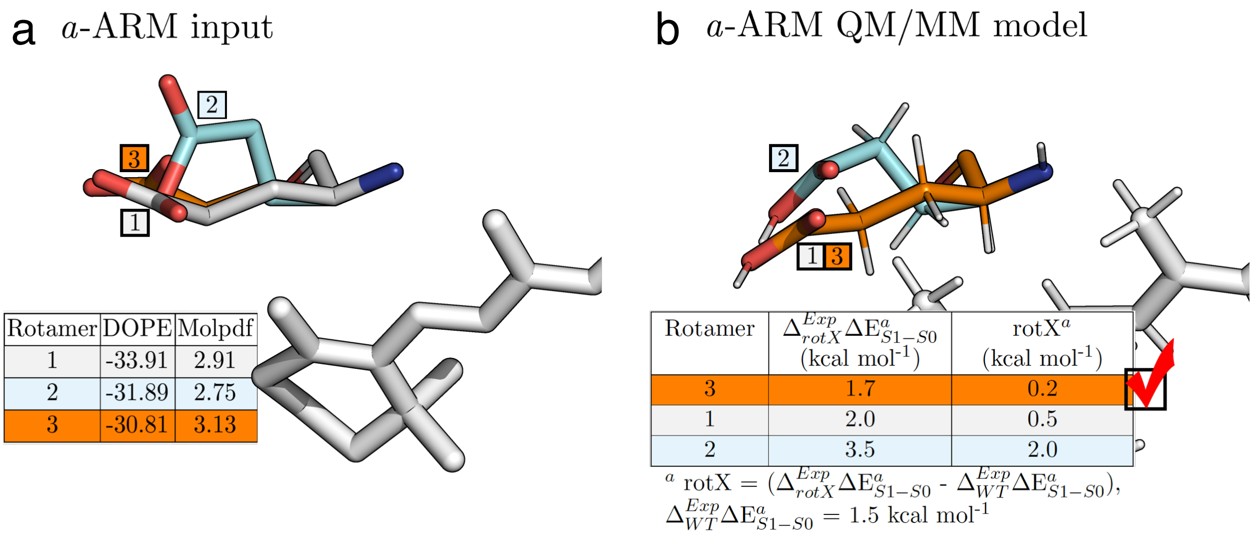

Rotamer	DOPE	Molpdf
1	-33.91	2.91
2	-31.89	2.75
3	-30.81	3.13

Rotamer	$\Delta^{Exp}_{rotX}\Delta E^{a}_{S1-S0}$ (kcal mol^{-1})	rotX^{a} (kcal mol^{-1})
3	1.7	0.2
1	2.0	0.5
2	3.5	2.0

^{a} rotX = ($\Delta^{Exp}_{rotX}\Delta E^{a}_{S1-S0}$ - $\Delta^{Exp}_{WT}\Delta E^{a}_{S1-S0}$), $\Delta^{Exp}_{WT}\Delta E^{a}_{S1-S0}$ = 1.5 kcal mol^{-1}

Supplementary Figure 9 Schematic representation of the procedure employed for the selection of the side-chain conformation in mutants' generation. The side-chain of the E219 residue of the KR2 rhodopsin, is modeled by using the procedure specified in **Supplementary Figure 9**. (a) First, the DOPE and molpdf scoring functions for all the possible rotamers are evaluated and the three best values are ranked. (b) Then, the *a*-ARM QM/MM model for each rotamer is generated and the rotamer model featuring the lower difference in vertical excitation energy with respect to experimental data (rotamer 3) is selected.

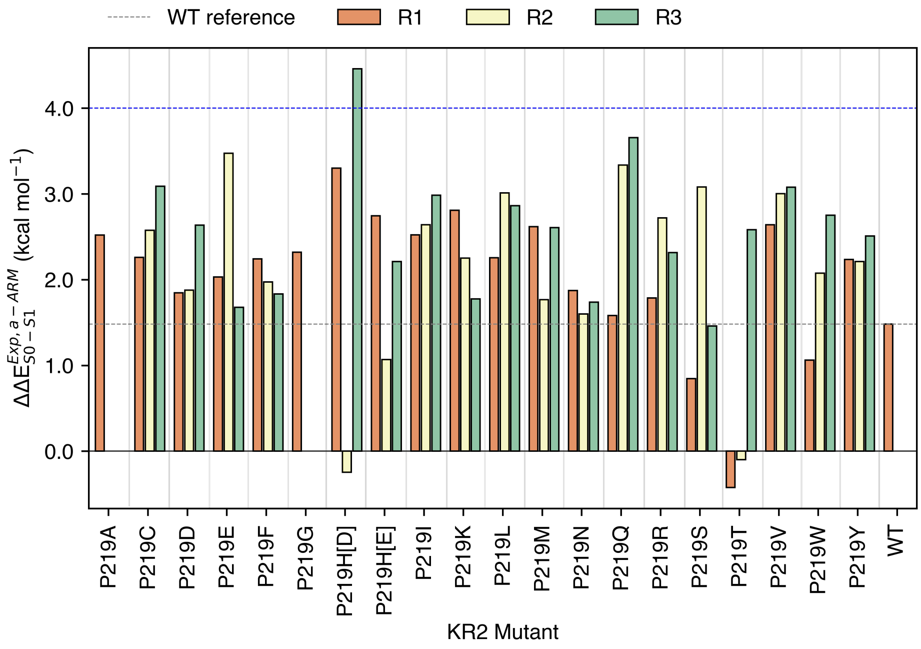

Supplementary Figure 10 Rotamers selection. Difference between calculated and experimental vertical excitation energy for each of the three default rotamers.

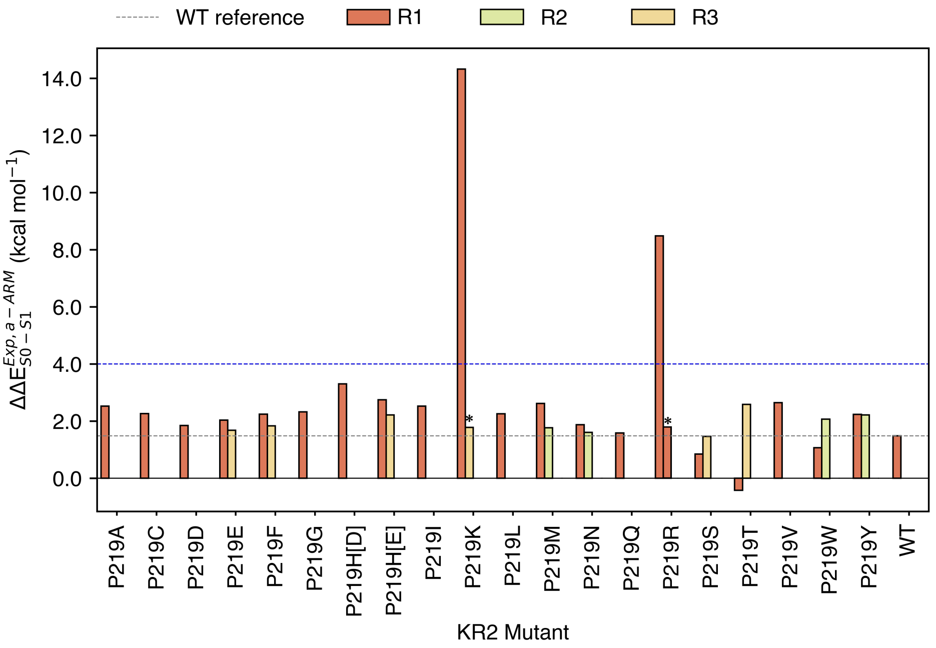

Supplementary Figure 11 Difference between calculated and observed vertical excitation energies for the default (first bar) and customized models (second bar). The default models have the same protonation states than the WT template and their side-chain is modelled with the rotamer 1 (dark orange bars). The side-chain of the customized models could be modelled with the rotamer 2 (green bars) or the rotamer 3 (light orange bars). Most of the customized models exhibit the same protonation states than the WT, with exception of P219K and P219R marked with a star.

- Limitations and pitfalls of side-chain predictor

- *Insufficient description of possible cavity rearrangements after mutation*: The procedure for modeling the side-chain conformation comprises a short MD^{mod}, where the produced side-chain is allowed to relax, whereas the rest of the cavity residues, waters, chromophore and protein environment remain fixed at crystallographic/comparative structure. Therefore, possible local steric/electronic rearrangements of the residues of the chromophore cavity surrounding the mutated residue are not correctly described. Although during the QM/MM generation phase of *a*-ARM the geometry of this side-chain along with the side-chain of the residues in the chromophore cavity are refined via a more sophisticated Molecular Dynamics (MD) (see Section S1.2.1), in some cases this step would not be sufficient to achieve a proper description of the impact of the new side-chain on the protein environment.

- *Mutations only allowed in the chromophore cavity:* Currently, *a*-ARM only allows mutations of residues that belong to the chromophore cavity sub-system, as well as backbone relaxation is not allowed. The latter is to ensure that, during the QM/MM model generator phase the geometry of the new modeled side-chain as well as the side-chain of its neighbors (belonging to the chromophore cavity) can be re-adjusted during the 1ns GROMACS MD step, while assuming that the general structure of the protein is conserved.

- *Lack of a predictive tool:* The fact that the mutant's generator relies on the use of experimental data to select the correct rotamer, limits the usability of the protocol that cannot be considered as a predictor tool.

Supplementary References

[1] Laura Pedraza-González, Luca De Vico, Marla Del Carmen Marln, Francesca Fanelli, and Massimo Olivucci. *a*-ARM: Automatic Rhodopsin Modeling with Chromophore Cavity Generation, Ionization State Selection, and External Counterion Placement. *J. Chem. Theory Comput.*, 15(5):3134–3152, 5 **2019**.

[2] Benjamin Webb and Andrej Sali. Comparative protein structure modeling using MODELLER. *Curr Protoc Bioinformatics*, 54(1):5–6, **2016**.

[3] Mats H.M. Olsson, Chresten R. Søndergaard, Michal Rostkowski, and Jan H. Jensen. PROPKA3: Consistent Treatment of Internal and Surface Residues in Empirical pKa Predictions. *J. Chem. Theory Comput.,* 7(2):525–537, **2011**.

[4] Federico Melaccio, María Del Carmen Marín, Alessio Valentini, Fabio Montisci, Silvia Rinaldi, Marco Cherubini, Xuchun Yang, Yoshitaka Kato, Michael Stenrup, Yoelvis Orozco-Gonzalez, Nicolas Ferré, Hoi Ling Luk, Hideki Kandori, and Massimo Olivucci. Toward Automatic Rhodopsin Modeling as a Tool for High-Throughput Computational Photobiology. *J. Chem. Theory Comput.*, 12(12):6020–6034, 12 **2016**.

[5] Vincent Le Guilloux, Peter Schmidtke, and Pierre Tuffery. Fpocket: An Open Source Platform for Ligand Pocket Detection. *Bioinformatics*, 10(1):168–179, **2009**.

[6] Laura Pedraza-González, María del Carmen Marín, Luca De Vico, Xuchun Yang, and Massimo Olivucci. On the Automatic Construction of QM/MM Models for Biological Photoreceptors: Rhodopsins as Model Systems. In *QM/MM Studies of Light responsive Biological Systems*, pages 1–75. Springer, **2020**.

[7] Li Zhang and Jan Hermans. Hydrophilicity of cavities in proteins. *Proteins: Struct., Funct., Bioinf.*, 24(4):433–438, **1996**.

[8] Sander Pronk, Szilárd Páll, Roland Schulz, Per Larsson, Pär Bjelkmar, Rossen Apostolov, Michael R Shirts, Jeremy C Smith, Peter M Kasson, David Van Der Spoel, Berk Hess, and Erik Lindahl. GROMACS 4.5: A High-Throughput and Highly Parallel Open Source Molecular Simulation Toolkit. *Bioinformatics*, 29(7):845–854, **2013**.

[9] Wendy D. Cornell, Piotr Cieplak, Christopher I. Bayly, Ian R. Gould, Kenneth M. Merz, David M. Ferguson, David C. Spellmeyer, Thomas Fox, James W. Caldwell, and Peter A. Kollman, A Second Generation Force Field for the Simulation of Proteins, Nucleic Acids, and Organic Molecules. *J. Am. Chem. Soc.,* 117(19): 5179–5197, **1995**.

[10] Joshua A. Rackers, Zhi Wang, Chao Lu, Marie L. Laury, Louis Lagardére, Michael J. Schnieders, Jean-Philip Piquemal, Pengyu Ren, and Jay W. Ponder. Tinker 8: Software tools for molecular design. *J. Chem. Theory Comput.*, 14(10):5273–5289, **2018**.

[11] Francesco Aquilante, Jochen Autschbach, Alberto Baiardi, Stefano Battaglia, Veniamin A Borin, Liviu F Chibotaru, Irene Conti, Luca De Vico, Mickaël Delcey, Ignacio Fdez. Galván, Nicolas Ferré, Leon Freitag, Marco Garavelli, Xuejun Gong, Stefan Knecht, Ernst Larsson, Roland Lindh, Marcus Lundberg, Per-Ake Malmqvist, Artur Nenov, Jesper Norell, Michael Odelius, Massimo Olivucci, Thomas Pedersen, Laura Pedraza-González, Quan Phung, Kristine Pierloot, Markus Reiher, Igor Schapiro, Javier Segarra-Martí, Francesco Segatta, Luis Seijo, Saumik Sen, Dumitru-Claudiu Sergentu, Christopher Stein, Liviu Ungur, Morgane Vacher, Alessio Valentini, and Valera Veryazov. Modern quantum chemistry with [Open] Molcas. *J. Chem. Phys.*, 152(21):214117, **2020**.

[12] Francesco Aquilante, Jochen Autschbach, Rebecca K. Carlson, Liviu F. Chibotaru, Mickaël G. Delcey, Luca De Vico, Ignacio Fdez. Galván, Nicolas Ferré, Luis Manuel Frutos, Laura Gagliardi, Marco Garavelli, Angelo Giussani, Chad E. Hoyer, Giovanni Li Manni, Hans Lischka, Dongxia Ma, Per Åke Malmqvist, Thomas Müller, Artur Nenov, Massimo Olivucci, Thomas Bondo Pedersen, Daoling Peng, Felix Plasser, Ben Pritchard, Markus Reiher, Ivan Rivalta, Igor Schapiro, Javier Segarra-Martí, Michael Stenrup, Donald G. Truhlar, Liviu Ungur, Alessio Valentini, Steven Vancoillie, Valera Veryazov, Victor P. Vysotskiy, Oliver Weingart, Felipe Zapata, and Roland Lindh. Molcas8: New capabilities for multiconfigurational quantum chemical calculations across the periodic table. *J. Comput. Chem.*, 37(5):506–541, 11 **2016**.

[13] Nicolas Ferré, János G. Ángyán. Approximate electrostatic interaction operator for QM/MM calculations, *Chem. Phys. Lett,* 356(3-4): 331-339, **2002**.

[14] Daniele Loco, Louis Lagardère, Stefano Caprasecca, Filippo Lipparini, Benedetta Mennucci, and Jean-Philip Piquemal. Hybrid QM/MM molecular dynamics with AMOEBA polarizable embedding, *J. Chem. Theory Comput.,* 13:4025-4033, **2017**.

[15] N. F. a. M. O. Tadeusz Andruniów, "Structure, initial excitedstate relaxation, and energy storage of rhodopsin resolved at the multiconfigurational perturbation theory level.," *Proceedings of the National Academy of Sciences of the United States of America,* vol. 12, no. 101(52), p. 17908–1791, **2004**.

[16] Maria-Andrea Mroginski, Suliman Adam, Gil S. Amoyal, Avishai Barnoy, AnaNicoleta Bondar, Veniamin Borin, Jonathan R. Church, Tatiana Domratcheva, Bernd Ensing, Francesca Fanelli, Nicolas Ferré, Ofer Filiba, Laura Pedraza-González, Ronald González, Cristina E. González-Espinoza, Rajiv K. Kar, Lukas Kemmler, Seung Soo Kim, Jacob Kongsted, Anna I. Krylov, Yigal Lahav, Michalis Lazaratos, Qays NasserEddin, Isabelle Navizet, Alexander Nemukhin, Massimo Olivucci, Jógvan Magnus Haugaard Olsen, Alberto Pérez de Alba Ortíz, Elisa Pieri, Aditya G. Rao, Young Min Rhee, Niccolò Ricardi, Saumik Sen, Ilia A. Solov'yov, Luca De Vico, Tomasz A. Wesolowski, Christian

Wiebeler, Xuchun Yang, Igor Schapiro, "Frontiers in Multiscale Modeling of Photoreceptor Proteins, *Photochem. Photobiol,* 97(2):243-269, **2021**.

[17] Rodrigo Ochoa, Miguel A Soler, Alessandro Laio, and Pilar Cossio. Assessing the capability of in silico mutation protocols for predicting the finite temperature conformation of amino acids. *Phys. Chem. Chem. Phys.*, 20(40):25901–25909, **2018**.

[18] Andrei Ignatov, Statistical Analysis of Protein Side-chain Conformations, *J. Phys. Conf. Ser.,* vol. 1740, p. 012013, **2021**.

[19] Zhexin Xiang and Barry Honig. Extending the accuracy limits of prediction for sidechain conformations. *J. Mol. Biol.*, 311(2):421–430, **2001**.

[20] Charles Wilson, Lydia M Gregoret, and David A Agard. Modeling side-chain conformation for homologous proteins using an energy-based rotamer search. *J. Mol. Biol.*, 229(4):996–1006, **1993**.

[21] Roland L Dunbrack Jr and Martin Karplus. Backbone-dependent proteins library for proteins application to side-chain prediction. *J. Mol. Biol.*, 230(2):543–574, **1993**.

[22] Maximiliano Vasquez. Modeling side-chain conformation. *Curr. Opin. Struct. Biol.*, 6(2):217–221, **1996**.

[23] Hidetoshi Kono and Junta Doi. A new method for side-chain conformation prediction using a hopfield network and reproduced rotamers. *J. Comput. Chem*, 17(14):1667-1683, **1996**.

[24] Adrian A Canutescu, Andrew A Shelenkov, and Roland L Dunbrack Jr. A graph theory algorithm for rapid protein side-chain prediction. *Protein Sci.*, 12(9):2001-2014, **2003**.

[25] Lenna X Peterson, Xuejiao Kang, and Daisuke Kihara. Assessment of protein sidechain conformation prediction methods in different residue environments. *Proteins: Struct., Funct., Bioinf.*, 82(9):1971–1984, **2014**.

[26] Zhichao Miao, Yang Cao, and Taijiao Jiang. Rasp: rapid modeling of protein side chain conformations. *Bioinformatics*, 27(22):3117–3122, **2011**.

[27] Shide Liang, Dandan Zheng, Chi Zhang, and Daron M Standley. Fast and accurate prediction of protein side-chain conformations. *Bioinformatics*, 27(20):2913–2914, **2011**.

[28] Roland L Dunbrack Jr. Rotamer libraries in the 21st century. Curr. Opin. *Struct. Biol.,* 12(4):431–440, **2002**.

[29] Georgii G Krivov, Maxim V Shapovalov, and Roland L Dunbrack. Improved Prediction of Protein Side-Chain Conformations with SCWRL4. *Proteins: Struct., Funct., Bioinf.*, 77(4):778-795, 2009.

[30] Georgii G Krivov, Maxim V Shapovalov, and Roland L Dunbrack. Improved Prediction of Protein Side-Chain Conformations with SCWRL4. *Proteins: Struct., Funct., Bioinf.*, 77(4):778-795, **2009**.

[31] M. D. C. Marín, D. Agathangelou, Y. Orozco-Gonzalez, A. Valentini, Y. Kato, R. Abe-Yoshizumi, H. Kandori, A. Choi, K.-H. Jung and S. e. a. Haacke, "Fluorescence enhancement of a microbial rhodopsin via electronic reprogramming.," *J. Am. Chem. Soc.,* vol. 141, p. 262– 271, **2019**.

[32] María Del Carmen Marín, Damianos Agathangelou, Yoelvis Orozco-Gonzalez, Alessio Valentini, Yoshitaka Kato, Rei Abe-Yoshizumi, Hideki Kandori, Ahreum Choi, Kwang Hwan Jung, Stefan Haacke, and Massimo Olivucci. Fluorescence Enhancement of a Microbial Rhodopsin via Electronic Reprogramming. *J. Am. Chem. Soc.,* 141(1):262–271, 1 **2019**.

[33] Keiichi Inoue, María del Carmen Marín, Sahoko Tomida, Ryoko Nakamura, Yuta Nakajima, Massimo Olivucci, and Hideki Kandori. Red-shifting Mutation of Light driven Sodium-pump Rhodopsin. *Nat. Commun.*, 10(1):1993, **2019**.

[34] Feyfant, Eric, Andrej Sali, and András Fiser. Modeling mutations in protein structures. *Protein Sci*. 16(9): 2030-2041, **2009**.

[35] Min-yi Shen and Andrej Sali. Statistical potential for assessment and prediction of protein structures. *Protein Sci.,* 15(11):2507–2524, **2006**.

[36] Ken Nagata, Arlo Randall, and Pierre Baldi. Sidepro: A novel machine learning approach for the fast and accurate prediction of side-chain conformations. *Proteins: Struct., Funct., Bioinf.*, 80(1):142–153, **2012**.
